# On the Mechanism of Random Handedness Generation in the Reactions of Heterocyclic Aldehydes with Diallylboronates

**DOI:** 10.3390/molecules31010128

**Published:** 2025-12-30

**Authors:** Oleg Mikhailov, Ilya D. Gridnev

**Affiliations:** N. D. Zelinsky Institute of Organic Chemistry, Russian Academy of Sciences, Leninsky Prosp. 47, 119911 Moscow, Russia

**Keywords:** chirality generation, asymmetric allylation, DFT computations

## Abstract

The mechanism of generation of products with opposite handedness in the reactions of heterocyclic aldehydes with diallylboronates was studied by NMR experiments and DFT computations. The origin of this unusual phenomenon is a competition between monomeric and dimeric autoinductors that promote the formation of opposite enantiomers. Thus, NMR data suggest that racemic alcohol **3a**, upon dimerization, provides almost exclusively the heterochiral dimeric boronate **5a**(*RS*). This corresponds to the computed results predicting strongly exergonic dimerization with ΔΔG^298^ −6.5 kcal/mol. Dimerization of the chiral boronate **3a** (*R*) with 82% ee yields **5a** (*RS*) in which all available **3a**(*S*) is bound. As a result, 3 species remain in the solution: (1) **5a**(*RS*), producing a newly formed racemic product in the reaction with **1a**, (2) **3a**(*R*), reacting with 1a and yielding an *R*-configured newly formed product, and (3) **5a**(*RR*), yielding selectively S-configured newly formed product according to computations. Taking into account the equilibria existing between monomers and dimers, the system is capable of demonstrating the experimentally observed random handedness of the newly formed product.

## 1. Introduction

Enantioselective synthesis is an important area of contemporary organic chemistry due to the need for producing chiral drugs [1], catalysts, polymers, and other materials requiring enantiomeric purity [2]. This explains the interest of researchers in the intrinsic mechanisms of the generation of chiral centers [3].

The Soai reaction, viz. alkylation of specifically substituted pyrimidinic aldehydes with diisopropylzinc [4,5,6,7,8], is especially interesting for this field, since it can lead to the formation of chiral products from non-chiral precursors [9,10,11,12]. Proof of authentic spontaneous chirality generation is a stochastic (or close to stochastic) distribution of handedness in the reaction product [12,13,14,15,16].

Recently, we published investigation of the boundary conditions essential for the possibility of spontaneous chirality generation in the course of allylboration of triazolic aldehydes with triallylborane [17,18]. We concluded that this is theoretically possible, but for its actual realization, an accurate balance of the activation barriers for each stage of the catalytic cycle is required [18].

**Scheme 1:**
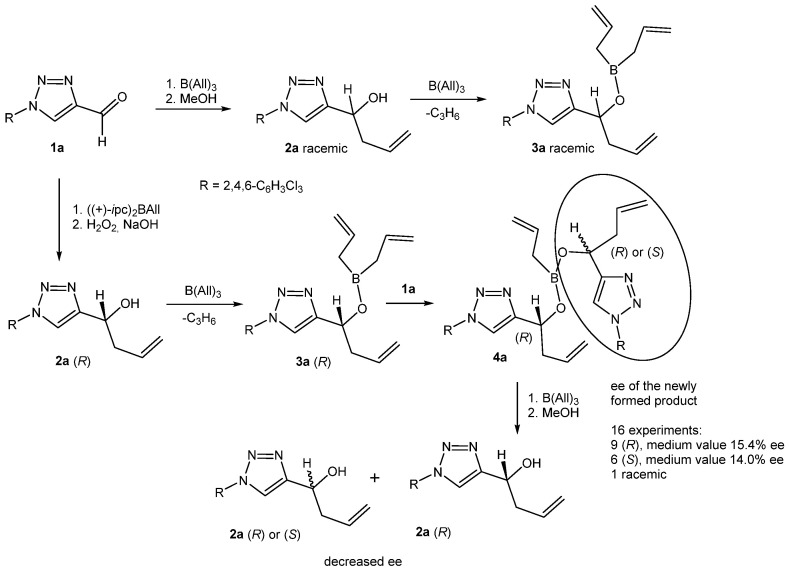
Close-to-stochastic distribution of handedness in the newly formed product **4a**.

During this investigation, we unexpectedly found that in the reaction of chiral boronate **3a** with triazole aldehyde **1a**, the handedness of the newly formed product varied from one experiment to another. Thus, in 16 experiments, the newly formed product had an *R* configuration (the same as **2a**) 9 times and an *S* configuration 6 times, while in one experiment, the newly formed product was racemic (Scheme 1) [18]. In this paper, we reveal a possible mechanism for this phenomenon.

## 2. Results

**Scheme 2:**
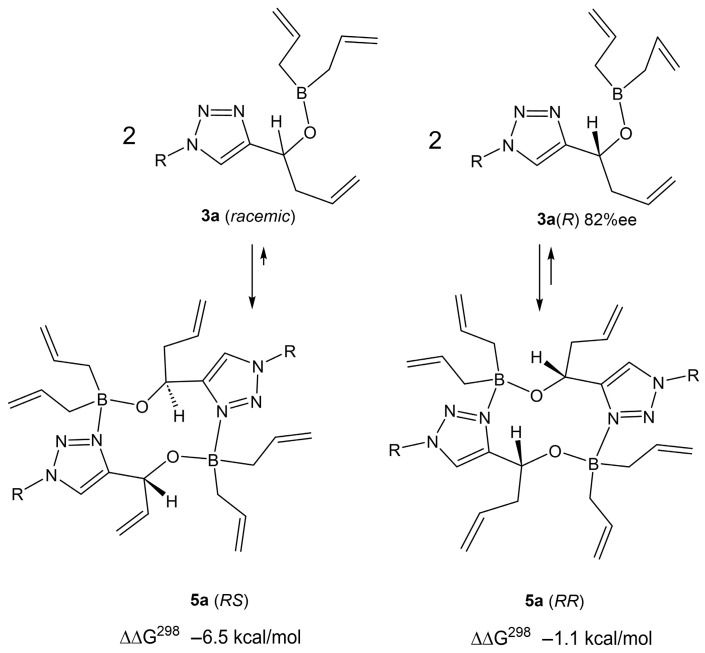
Computed Gibbs free energies for the formation of the heterochiral dimer **5a**(*RS*) and the homochiral dimer **5a**(*RR*) [18].

**Figure 1:**
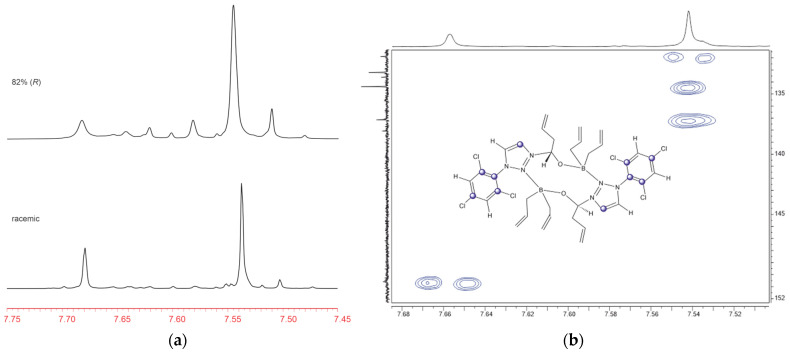
(**a**) Expansions of the ^1^H NMR spectra (600 MHz, CDCl_3_) of boronates prepared from racemic alcohol **2a** and **2a**(*R*) with 82% ee. (**b**) Expansions of the ^1^H-^13^C HMBC spectrum of boronate prepared from racemic alcohol **2a**.

Quantum chemical computations suggest that in solution, chiral boronates **3a**(*R*) exist in a dimeric form, with the heterochiral dimer **5a**(*RS*) being significantly more stable than the homochiral dimer **5a**(*RR*) (Scheme 2). We decided to verify this prediction experimentally. Two NMR samples were prepared containing equal concentrations of racemic alcohol **2a** and alcohol **2a** with 82% ee (*R*). An equivalent amount of triallylborane was added to each sample, yielding the ^1^H NMR spectra shown in Figure 1.

Comparing the spectra shown in Figure 1a, one can conclude that dimerization indeed takes place in the solutions of boronates because otherwise, the spectra would be identical. Moreover, it is possible to infer that the main component of the racemic sample is the heterochiral dimer **5a**(*RS*). This is supported by the fragment of the ^1^H-^13^C HMBC NMR spectrum shown in Figure 1b, which convincingly demonstrates that the most intense signal in the ^1^H spectrum does not overlap with any other signals, since exactly three cross-peaks with this signal are observed in the ^1^H-^13^C HMBC spectrum, as expected for two equivalent protons in the substituted phenyl ring in **5a**(*RS*).

On the other hand, in the spectrum of the scalemic boronate, additional significantly broadened signals are seen, which must belong to **5a**(*RR*) in fast equilibrium with **3a**(*R*). If the possibility of a further oligomerization is not considered, only **5a**(*RS*), **5a**(*RR*), and **3a**(*R*) co-exist in the solution, since the minor enantiomer **3a**(*S*) is strictly bound in **5a**(*RS*) (reservoir effect).

The boronate **3a**(*R*) was computed to react with **1a** with high *R*-enantioselectivity [18]. Although some competition from the *S*-pathway cannot be entirely excluded, it is unlikely to result in a reversal of the handedness of the newly formed product. Furthermore, the heterochiral dimer **5a**(*RS*) has reactive centers of both signs and might be capable of producing only a racemate, which again would not result in the formation of an (*S*)-enriched product. Hence, we hypothesized the possibility of **5a**(*RR*) being the source of the (*S*)-enantioselective reaction.

**Figure 2:**
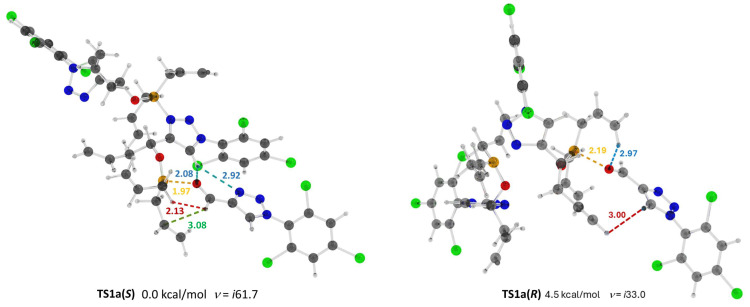
Structures of the diastereomeric transition states corresponding to the coordination of aldehyde **1a** to the dimer **5a**(*RR*) (Scheme 3). The **TS1a**(*S*) is significantly more stable due to a network of intermolecular non-covalent stabilizing interactions. Atoms: C black, H grey, Cl green, O red, N blue, B yellow. Interatomic distances: CH⋯HC red, CH⋯O and CH⋯N blue, CH⋯π, forming bonds, yellow.

**Scheme 3:**
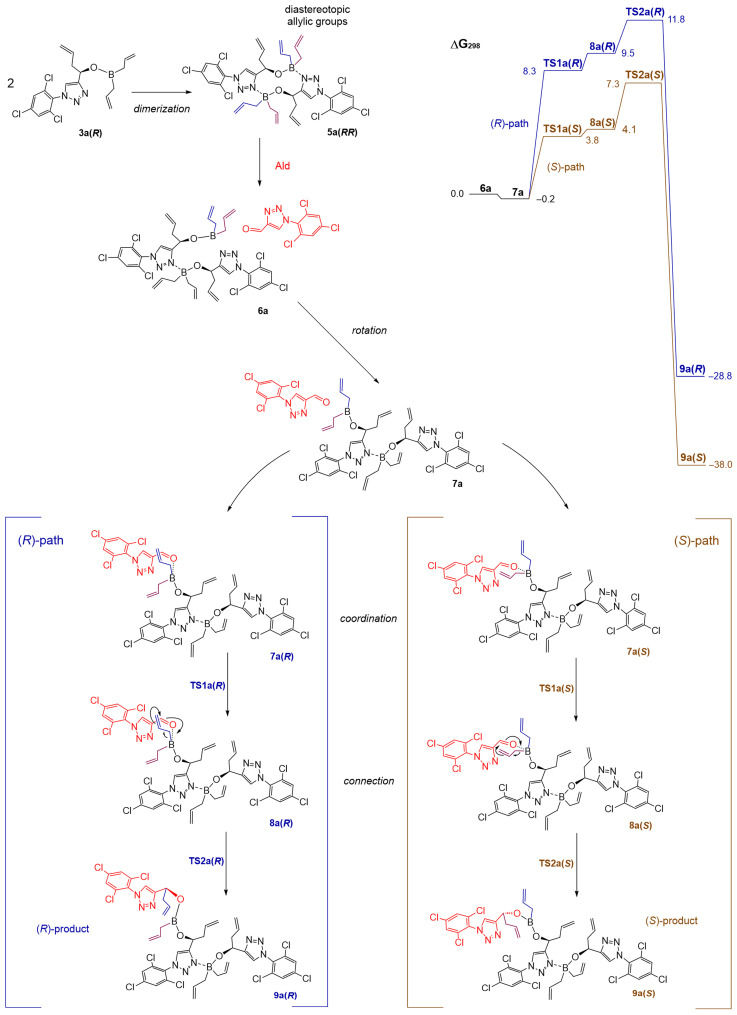
Dimerization of boronate **3a**(*R*) and the subsequent reaction with aldehyde **1a**. Computed ∆G_298_ values for this reaction. Structure of the incoming substrate is marked red.

We modeled the allylboration of **1a** using DFT computations (Scheme 3, Figure 2). The approach of **1a** to one of the boron atoms in **5a**(*RR*) results in the dissociation of the B-N coordinate bond. Simultaneously, a B-O bond is formed, yielding adduct **7a** with the aldehyde positioned between two diastereotopic allylic groups. This coordination requires overcoming free activation barriers of 3.8 and 8.3 kcal/mol for the *S*- and *R*-pathways, respectively, which indicates a significant predominance of the former.

**Figure 3:**
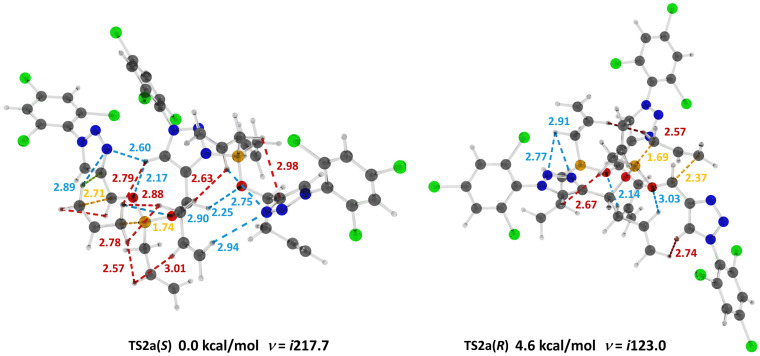
Structures of the diastereomeric transition states corresponding to the allylboration of aldehyde **1a** (Scheme 3). The **TS2a**(*S*) is significantly more stable due to network of intermolecular non-covalent stabilizing interactions. Atoms: C black, H grey, Cl green, O red, N blue, B yellow. Interatomic distances: CH⋯HC red, CH⋯O and CH⋯N blue, CH⋯π, forming bonds, yellow.

The allylboration stage is also *S*-stereoselective (Figure 3). Thus, our computations confirm the conclusion drawn from the experimental data: the reaction of dimer **5a**(*RR*) with aldehyde **1a** is *S*-stereospecific. Moreover, the computed activation parameters for this reaction are comparable to those of the *R*-stereospecific reaction of the *R*-monomer [18]; hence, these two reactions can compete. Furthermore, the heterochiral dimer **5a**(*RS*) can probably contribute to the reaction flux by yielding a racemate. Taking into account the equilibria existing between monomers and dimers, we arrive to a complex system that is capable of demonstrating the experimentally observed random handedness of the newly formed product.

**Scheme 4:**
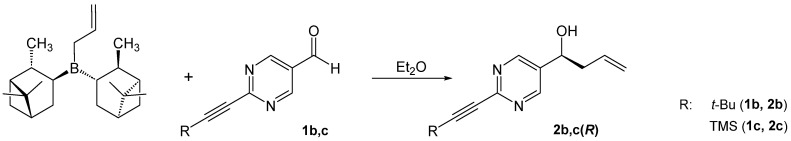
Synthesis of optically active alcohols based on Soai aldehydes.

**Scheme 5:**
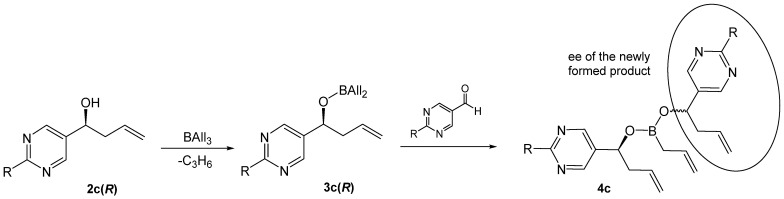
The allylboration of **1c** with optically active boronate **3c**(*R*).

**Figure 4:**
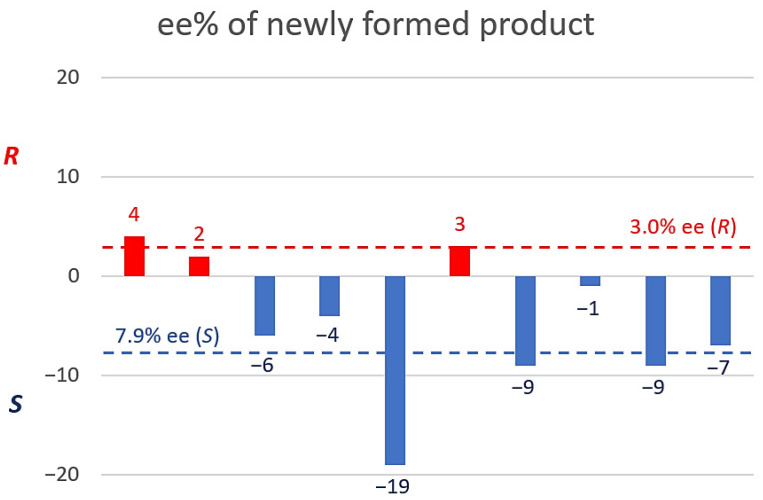
Results of 10 experiments for the allylboration of **1c** with optically active boronate **3c**(*R*). Mean values are shown as dashed lines.

Interested in extending the scope of the Soai reaction [17,18,19], we also investigated the reaction of aldehyde **1c** with chiral boronate **3c**(*R*) (Scheme 4), obtained via the reaction of **2c**(*R*) with triallylborane (Scheme 5). As can be seen from Figure 4, random handedness of the newly formed product is also observed in this reaction.

**Scheme 6:**
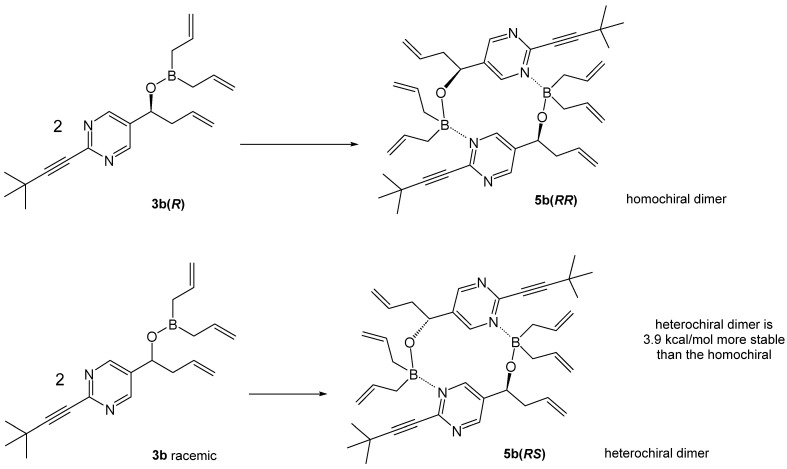
Computed Gibbs free energies for the formation of the heterochiral dimer **5b**(*RS*) and the homochiral dimer **5b**(*RR*).

**Figure 5:**
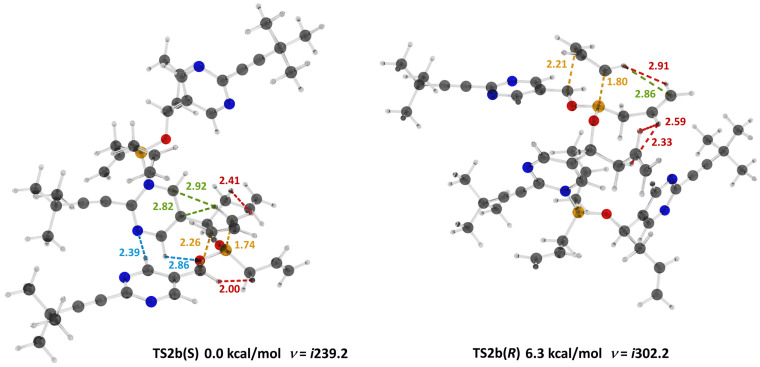
Structures of the diastereomeric transition states corresponding to the allylboration of aldehyde **1b** (Scheme 7). The **TS2b**(*S*) is significantly more stable due to a network of intermolecular non-covalent stabilizing interactions. Atoms: C black, H grey, Cl green, O red, N blue, B yellow. Interatomic distances: CH⋯HC red, CH⋯O and CH⋯N blue, CH⋯π, forming bonds, yellow.

The ^1^H NMR spectra for racemic and chiral boronates **3b**(*R*) were notably different, indicating the occurrence of oligomerization in solution. Similarly to the previous case, the heterochiral dimer **5b**(*RS*) was computed to be 3.9 kcal/mol more stable than the homochiral dimer **5b**(*RR*) (Scheme 6). A significant predominance of the *S*-pathway was computed for **5b**(*RR*) (Figure 5), leading to the conclusion that the mechanism of random handedness generation of the newly formed product is the same as in the case of **5a**(*RR*).

**Scheme 7:**
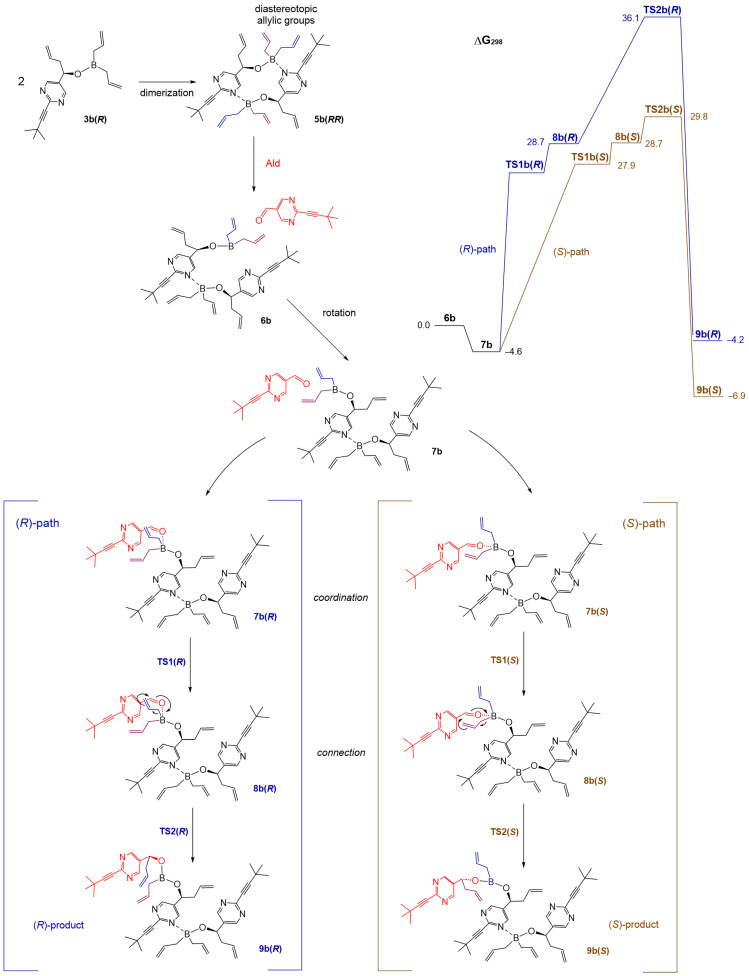
Dimerization of boronate **3b**(*R*) and the subsequent reaction with aldehyde **1b**. Computed ∆G_298_ values for this reaction. Structure of the incoming substrate is marked red.

## 3. Discussion

Our combined experimental and computational study revealed the most probable source of random handedness generation in the reactions of chiral boronates with the corresponding aldehydes. In both cases, reactions of chiral monomers and dimers of the same chirality yield products with opposite handedness. Equilibria between monomers, homo- and heterodimers are affected by the concentrations of the components, which change as the reaction proceeds, introducing randomness in the handedness of the newly formed product.

The similar behavior of two structurally different aldehydes suggests that this might be a common feature for reactions with organometallic compounds prone to oligomerization, where different oligomers promote the formation of products with opposite handedness.

We have recently reported a similar phenomenon in the reaction of diisopropylzinc with triazole aldehydes [19]. In addition, numerous examples of various chiral initiators capable of providing an initial imbalance in the enantiomeric ratio are known, including inorganic crystals [20,21] and organic compounds with various types of chirality or crypto-chirality [7]. However, in all these examples the formation of only one enantiomer of the product is triggered and can be further amplified via the effective asymmetric autoamplification characteristic of the Soai reaction [11]. The random handedness generation reported here resembles spontaneous chirality generation in the Soai reaction [12,13,14,15,16], where the experimentally observed randomness of the product chirality serves as an indicator of the authenticity of this occurrence.

These findings underline the uniqueness of the powerful mechanism of autoamplification observed in the Soai reaction. On the other hand, the stochastic generation of random handedness in the reactions of heterocyclic organometallic compounds seems to be a widespread phenomenon that under certain conditions might induce other enantioselective transformations.

## 4. Materials and Methods

### 4.1. Experimental Details

All reactions were carried out using standard Schlenk techniques under an argon atmosphere in oven-dried glassware with magnetic stirring. All solvents were purified and distilled using standard procedures. Solvents were additionally degassed by three pump–freeze–thaw cycles. Analytical thin layer chromatography (TLC) was carried out on TLC plates (Merk, Darmstadt, Germany) (silica gel 60 F_254_, 0.25 mm) using UV light (254 nm) as the visualizing agent. Silica gel 60A (Acros Organics, Geel, Belgium, 400–230 mesh, 0.040–0.063 mm) was used for open-column chromatography. NMR spectra were measured on Avance 300 and Avance 600 spectrometers (Bruker, Billerica, MA, USA) at 300.13 MHz (^1^H) and 75.47 MHz (^13^C) and 600.13 MHz (^1^H) and 150.90 MHz (^13^C), respectively at 20 °C in deuterated chloroform. The chemical shifts (*d*) are expressed in parts per million (ppm) and are calibrated using the residual undeuterated solvent peak as an internal reference (CDCl_3_: *δ*_H_ 7.26, *δ*_C_ 77.16). All coupling constants (*J*) are reported in Hertz (Hz), and multiplicities are indicated as follows: s (singlet), d (doublet) and m (multiplet). High-resolution mass spectra (HRMS) were obtained through electrospray ionization (ESI) with positive (+) ion detection on micrOTOF–QIII quadrupole time-of-flight mass spectrometer (Bruker, Billerica, MA, USA). The ee measurements were performed via HPLC analysis. The *ee* measurements were performed via HPLC analysis on an HPLC system equipped with chiral stationary phase columns (AD-H, AS-H, OD-H, OJ-H), with detection at 220 or 254 nm. Synthetic procedures and characterization details for the new compounds can be found in the Supplementary Materials.

### 4.2. Chemical Synthesis

Triallylborane [22], (+)-B-allyldiisopinocampheylborane [23], 2-((trimethylsilyl)ethynyl)pyrimidine-5-carbaldehyde [16] and 2-(3,3-dimethylbut-1-yn-1-yl)pyrimidine-5-carbaldehyde [24] were prepared by known procedure.

### 4.3. Computationall Details

Geometry optimizations were performed without any symmetry constraints (C1 symmetry) using the ωB97XD functional [25] as implemented in the Gaussian 09 software package [26]. Frequency calculations were undertaken to confirm the nature of the stationary points, yielding one imaginary frequency for all transition states (TSs) and zero for all minima. Constrained energy hypersurface scans were conducted to investigate the molecular reactivity and to locate viable reaction channels. Where low-lying barriers were estimated, frequency calculations were performed at the crude saddle points, and the obtained force constants were used to optimize the transition state structures employing the Berny algorithm [27]. All atoms were described with the 6–31G(d,p) basis set in geometry optimization and frequency calculation [28,29,30,31,32,33]. Non-specific solvation was introduced by using the SMD continuum model [34] (diethyl ether).

## 5. Conclusions

Counterintuitively, spontaneous generation of chirality with random handedness seems to be a widespread phenomenon in the reactions of organometallic compounds. It is rarely observed experimentally, since in the vast majority of cases, the initial effect is further extinguished through the competing reactions of various oligomers yielding opposite enantiomers. On the other hand, the Asymmetric Autocatalytic Amplification, so far established only for the Soai reaction applying substrates with strictly defined structures, seems to pose a significant challenge to researchers trying to find other transformations of that kind. Nevertheless, any research in this field illuminates the sophisticated chemistry of chiral alcoholates that often results in unexpected effects on the optical yields of the corresponding reactions.

## Data Availability

All data are included in the manuscript and the Supplementary Materials.

## Supplementary Materials

The following supporting information can be downloaded at: https://www.mdpi.com/article/10.3390/molecules31010128/s1.
