# Supplementary material for: On the Mechanism of Random Handedness Generation in the Reactions of Heterocyclic Aldehydes with Diallylboronates
Source: Molecules. 2025 Dec 30;31(1):128. doi: 10.3390/molecules31010128 (PMC12787661; doi:10.3390/molecules31010128)
Supplement: Supplementary file 1 [file molecules-31-00128-s001.zip › molecules-4029591-supplementary.pdf]

Supplementary Materials

On the Mechanism of Random Handedness Generation in the Reactions of Heterocyclic Aldehydes with Diallylboronates

Oleg Mikhailov and Ilya D. Gridnev *

N. D. Zelinsky Institute of Organic Chemistry, Russian Academy of Sciences, Leninsky Prosp. 47, 119911 Moscow, Russia
* Correspondence: ilyaiochem@gmail.com

Content

1. Table S1. Reactions of optically active boronate (R)-3b with one equivalent of 1b (Figure 4) ....Error! Bookmark not defined.

2. Materials and Methods .... Error! Bookmark not defined.

3. Experimental section .... Error! Bookmark not defined.

4. NMR Spectra of alcohols 2b, 2c **7**

5. HMRS data of alcohols 2b, 2c .... Error! Bookmark not defined.**9**

6. HPLC data of alcohols 2b, 2c .... Error! Bookmark not defined.

7. Cartesian coordinates of optimized structures .... Error! Bookmark not defined.**17**

1. Table S1. Reactions of optically active boronate (*R*)-3c with one equivalent of 1c (Figure 4)

Starting compounds	ee(%) of starting 3c	ee(%)	ee(%) of newly formed 3c	conditions
(*R*)-**3c**	72	38	4(*R*)	Et$_2$O, r.t.
	70	36	2(*R*)	
		33	6(*S*)	
		32	4(*S*)	
	73	27	19(*S*)	
		38	3(*R*)	
		32	9(*S*)	
		36	1(*S*)	
		32	9(*S*)	
		33	7(*S*)	

2. Materials and Methods

2.1. General Information

All reactions were carried out using standard Schlenk techniques under an argon atmosphere in oven-dried glassware with magnetic stirring. All solvents were purified and distilled using standard procedures. Solvents were additionally degassed by three pump-freeze-thaw cycles. Analytical thin layer chromatography (TLC) was carried out on Merck TLC plates (silica gel 60 F_{254}, 0.25 mm) using UV light (254 nm) as the visualizing agent. Silica gel 60A (Acros Organics, 400–230 mesh, 0.040–0.063 mm) was used for open-column chromatography. NMR spectra were measured on a Bruker Avance 300 spectrometer at 300.13 MHz (^{1}H) and 75.47 MHz (^{13}C), Bruker Avance 600 spectrometer at 600.13 MHz (^{1}H) and 150.90 MHz (^{13}C) at 20 °C in the deuterated chloroform. The chemical shifts (*d*) are expressed in parts per million (ppm) and are calibrated using residual undeuterated solvent peak as an internal reference ($CDCl_3$: δ_H 7.26, δ_C 77.16). All coupling constants (*J*) are reported in Hertz (Hz), and multiplicities are indicated as follows: s (singlet), d (doublet) and m (multiplet). High-resolution mass spectra (HRMS) were obtained through electrospray ionization (ESI) with positive (+) ion detection on a Bruker micrOTOF–QIII quadrupole time-of-flight mass spectrometer. The ee measurements were performed via HPLC analysis. The *ee* measurements were performed via HPLC analysis on an HPLC system equipped with chiral stationary phase columns (AD-H, AS-H, OD-H, OJ-H), detection at 220 or 254 nm.

2.2. Chemical Synthesis

Triallylborane [1], (+)-B-allyldiisopinocampheylborane [2], 2-((trimethylsilyl)ethynyl)pyrimidine-5-carbaldehyde [3] and 2-(3,3-dimethylbut-1-yn-1-yl)pyrimidine-5-carbaldehyde [4], were prepared by known procedure.

1. Zakharkin; L. I., Stanko, V. I. Simple synthesis of triallylboron and some of its conversions. *Russ Chem Bull* **1960**, 9, 1774–1776.
2. Brown, H. C.; Jadhav, P. K. Asymmetric carbon-carbon bond formation via. beta.-allyldiisopinocampheylborane. Simple synthesis of secondary homoallylic alcohols with excellent enantiomeric purities. *J. Am. Chem. Soc.* **1983**, *105*, 2092–2093.
3. Gridnev, I. D.; Serafimov, J. M.; Quiney, H.; Brown, J.M. Reflections on spontaneous asymmetric synthesis by amplifying autocatalysis. *Org. Biomol. Chem.* **2003**, *1*, 3811–3819.
4. Athavale, S. V.; Simon, A.; Houk, K. N.; Denmark, S. E. Structural Contributions to Autocatalysis and Asymmetric Amplification in the Soai Reaction. *J. Am. Chem. Soc.* **2020**, *142*, 18387–18406.

3. Experimental section

General procedure for allylboration

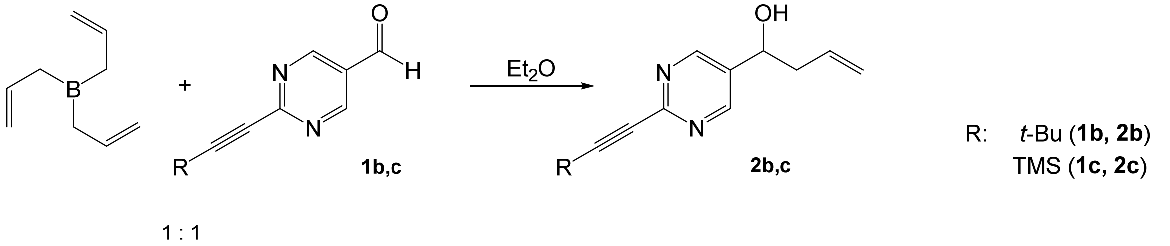

An aldehyde was placed in a 50 ml Schlenk flask equipped with a magnetic stirrer and a septa, filled with argon, and then evacuated. The argon—vacuum cycle was repeated three times, diethyl ether was added, mixed until the aldehyde was completely dissolved, then triallylborane was added drop by drop at room temperature. After 5 minutes, 5 mL of methanol was added. After stirring for 1 minute, all volatile components were removed in vacuum, the resulting residue was subjected to additional purification by column chromatography, eluting with a mixture of ethyl acetate with petroleum ether (1 : 1) to yield the corresponding alcohol.

1-(2-(3,3-Dimethylbut-1-yn-1-yl)pyrimidin-5-yl)but-3-en-1-ol (**2b**). It is obtained from aldehyde **1b** (20 mg, 0.11 mmol, 1 eq.) and triallylborane (14 mg, 0.11 mmol, 1 eq.) in ether (15 ml). Yield 21 mg (89%), colorless solid, R_f 0.20 (ethyl acetate—petroleum ether, 1:1). ^{1}H NMR ($CDCl_3$, 300 MHz, ppm): δ 8.66 (s, 2H), 5.82–5.73 (m, 1H, $C\underline{H}=CH_2$), 5.24–5.16 (m, 2H, $CH=C\underline{H}_2$), 4.84–4.79 (m, 1H, $C\underline{H}$–OH), 2.60–2.53 (m, 1H, $C\underline{H}_2$–CH=CH_2), 2.52–2.44 (m, 1H, $C\underline{H}_2$–CH=CH_2), 2.37 (d, J = 3.2 Hz, 1H, OH), 1.36 (s, 9H, TMS). ^{13}C {^{1}H} NMR ($CDCl_3$, 125 MHz, ppm): δ 155.29 (2$\underline{C}H_{Ar}$), 152.55, 134.52 (2C_{Ar}), 132.65 ($\underline{C}H=CH_2$), 120.50 ($CH=\underline{C}H_2$), 98.03, 78.59 (C≡C), 69.11 ($\underline{C}$H–O), 43.68 ($\underline{C}H_2$–CH=CH_2), 30.58 (3$\underline{C}H_3$(TMS)). HRMS (ESI) *m*/*z* 231.1492 (calcd for $C_{14}H_{18}N_2O$, 231.1492).

1-(2-((Trimethylsilyl)ethynyl)pyrimidin-5-yl)but-3-en-1-ol (**2c**). It is obtained from aldehyde **1c** (10 mg, 0.05 mmol, 1 eq.) and triallylborane (7 mg, 0.05 mmol, 1 eq.) in ether (10 ml). Yield 10 mg (83%), colorless oil, R_f 0.27 (ethyl acetate—petroleum ether, 1:1). ^{1}H NMR ($CDCl_3$, 300 MHz, ppm): δ 8.70 (s, 2H), 5.83–5.73 (m, 1H, $C\underline{H}=CH_2$), 5.26–5.17 (m, 2H, $CH=C\underline{H}_2$), 4.86–4.81 (m, 1H, $C\underline{H}$–OH), 2.62–2.55 (m, 1H, $C\underline{H}_2$–CH=CH_2), 2.52–2.45 (m, 1H, $C\underline{H}_2$–CH=CH_2), 2.23 (d, J = 3.2 Hz, 1H, OH), 0.29 (s, 9H, TMS). ^{13}C {^{1}H} NMR ($CDCl_3$, 125 MHz, ppm): δ 155.32 (2$\underline{C}H_{Ar}$), 151.72, 135.22 (2C_{Ar}), 132.53 ($\underline{C}H=CH_2$), 120.69 ($CH=\underline{C}H_2$), 102.31, 94.44 (C≡C), 69.08 ($\underline{C}$H–O), 43.70 ($\underline{C}H_2$–CH=CH_2), –0.33 (3$\underline{C}H_3$(TMS)). HRMS (ESI) *m*/*z* 247.1256 (calcd for $C_{13}H_{18}N_2OSi$, 247.1261).

General procedure for enantioselective allylboration

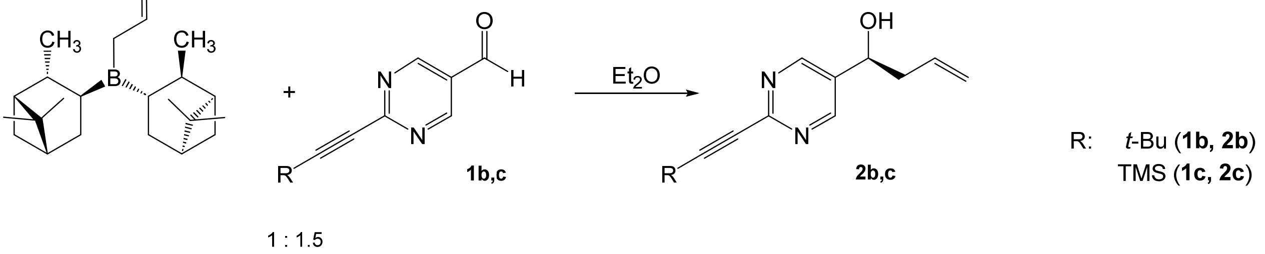

An aldehyde was placed in a 50 ml Schlenk flask equipped with a magnetic stirrer and a septa, filled with argon, and then evacuated. The argon—vacuum cycle was repeated three times, diethyl ether was added, mixed until the aldehyde was completely dissolved, then (+)-B-allyldiisopinocampheylborane was added drop by drop at room temperature. After stirring for 1 hour, an aqueous solution of NaOH (1%, 10 ml) and H_2O_2 (33%, 1 ml) was added to the reaction mixture and mixing was continued for another 30 minutes. The organic phase was separated, and the colorless aqueous layer was extracted with ethyl acetate (3×25 ml). The combined organic layers were evacuated to dry. The resulting gray-yellow residue was purified by column chromatography (eluent - ethyl acetate—petroleum ether, 1:1) to yield the corresponding alcohol. Samples were analyzed by chiral HPLC to determine *ee*.

General procedure for conducting reactions with optically active alcohols (Table 1)

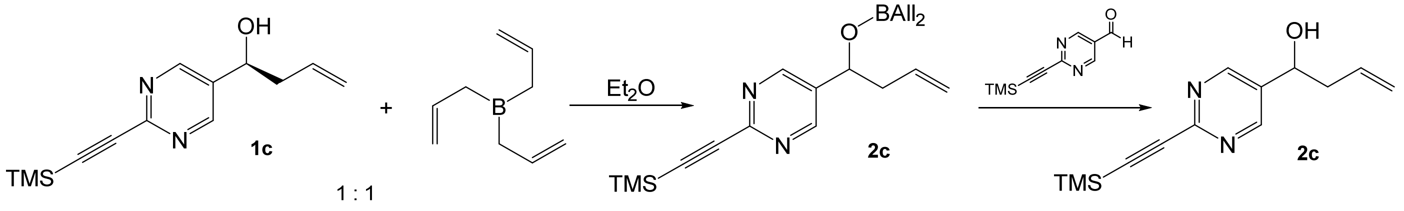

Chiral alcohol **2c** (12 mg, 0.05 mmol, 1 eq.) was placed in a 25 mL Schlenk flask equipped with a magnetic stirrer and a septum and filled with argon, repeating the argon-vacuum cycle 3 times. Then dry diethyl ether (10 mL) was added, stirred until the substance was completely dissolved and triallylborane (0.009 mL, 0.05 mmol, 1 eq.) was added. Aldehyde **1c** (10 mg, 0.05 mmol, 1 eq.) was dissolved in ether (5 mL) and was dipped into the reaction mixture. The reaction was monitored using TLC. In each case, the aldehyde conversion was complete. After 5 minutes, 5 mL of methanol was added. After stirring for 1 minute, all volatile components were removed in vacuum, the resulting residue was subjected to additional purification by column chromatography, eluting with a mixture of ethyl acetate with petroleum ether (1 : 1).

4. NMR Spectra of alcohols 2b, 2c

a)

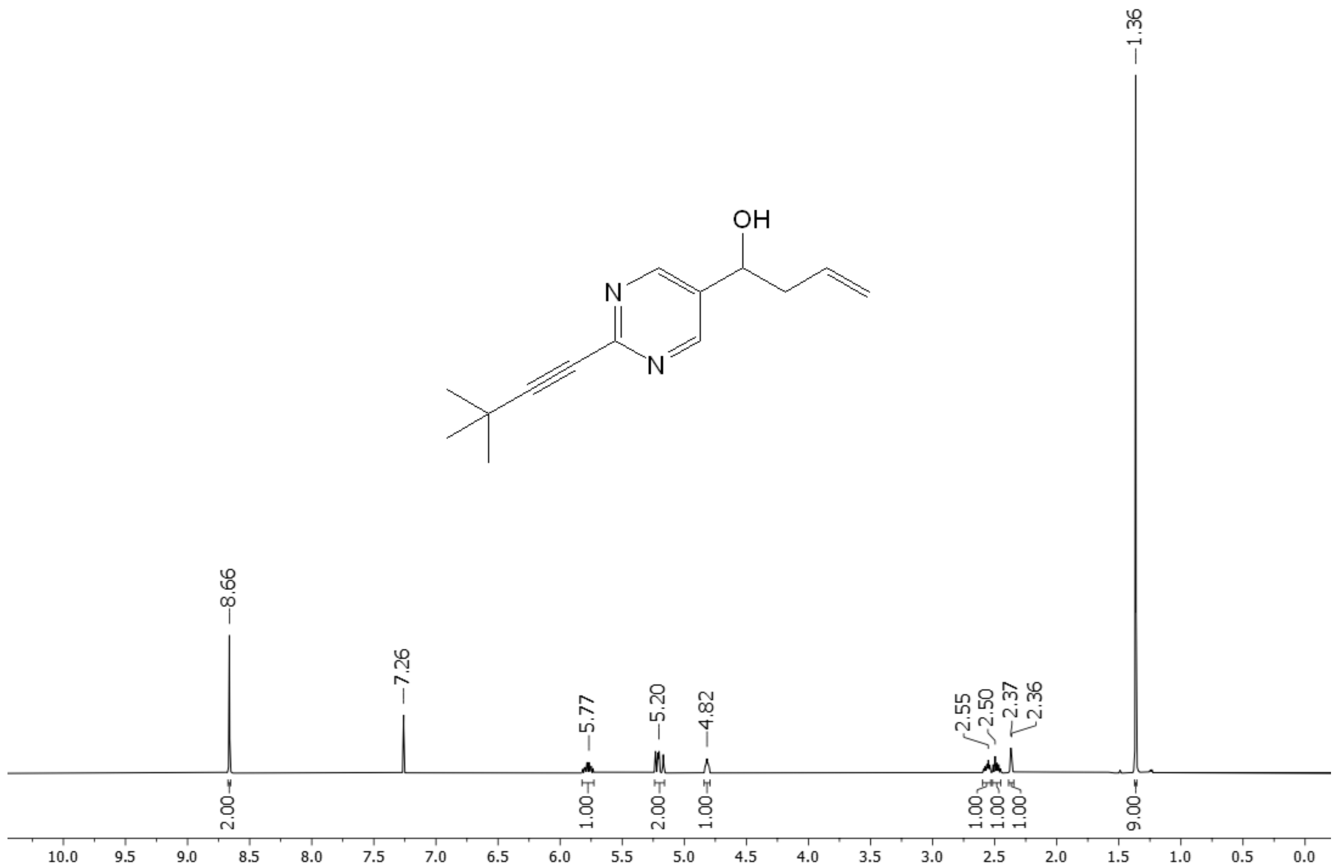

b)

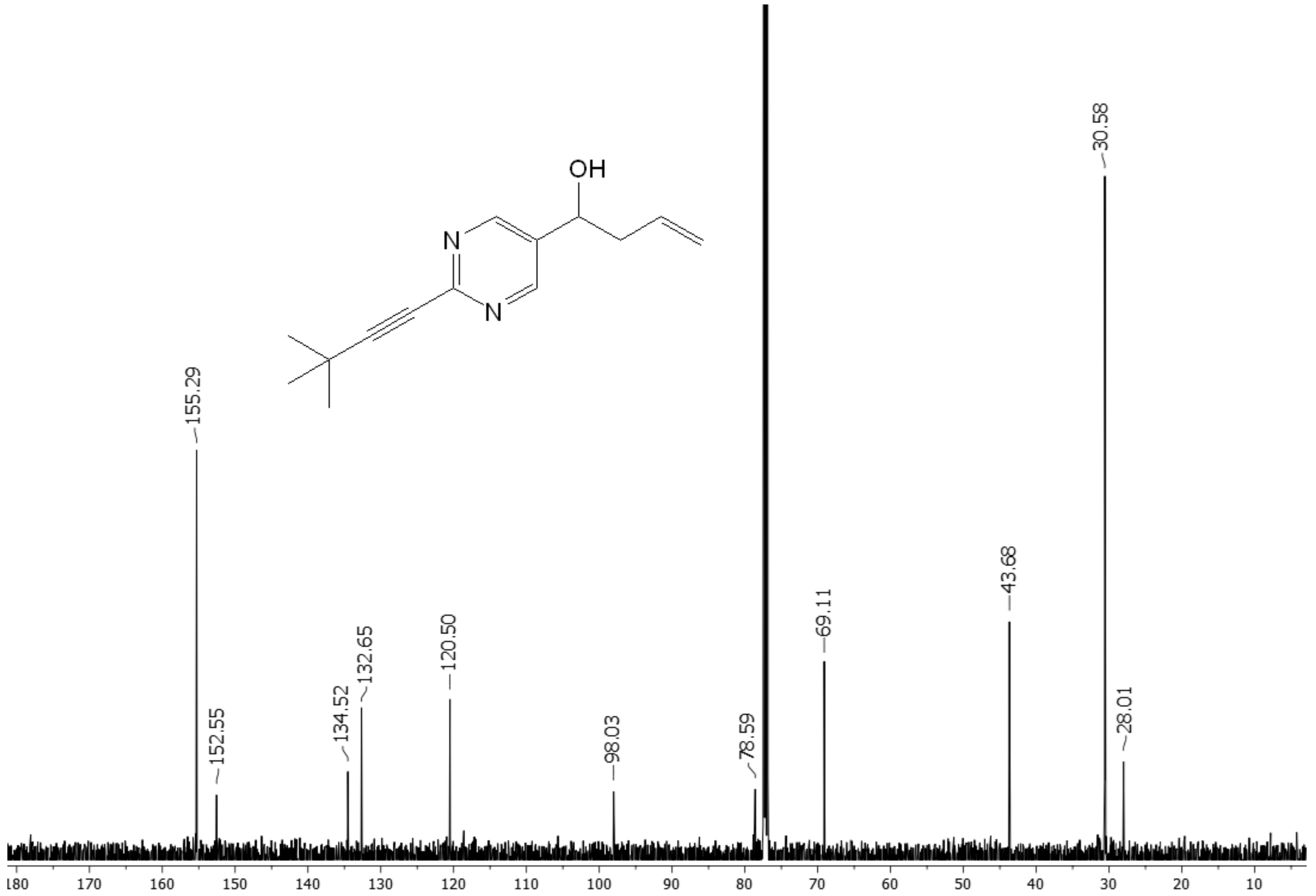

Figure S1. ^{1}H (a) and $^{13}C\{^{1}H\}$ (b) NMR spectra of compound **2b** ($CDCl_3$).

a)

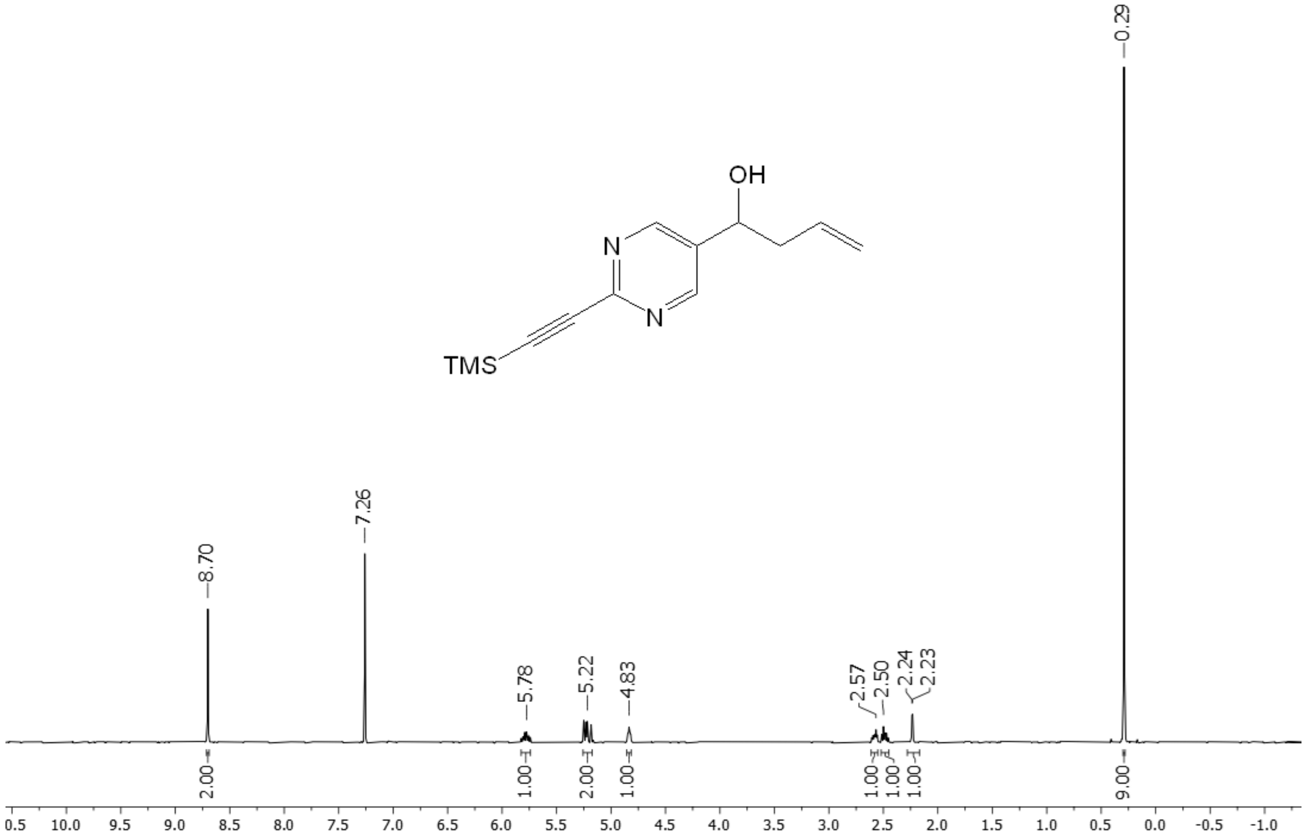

b)

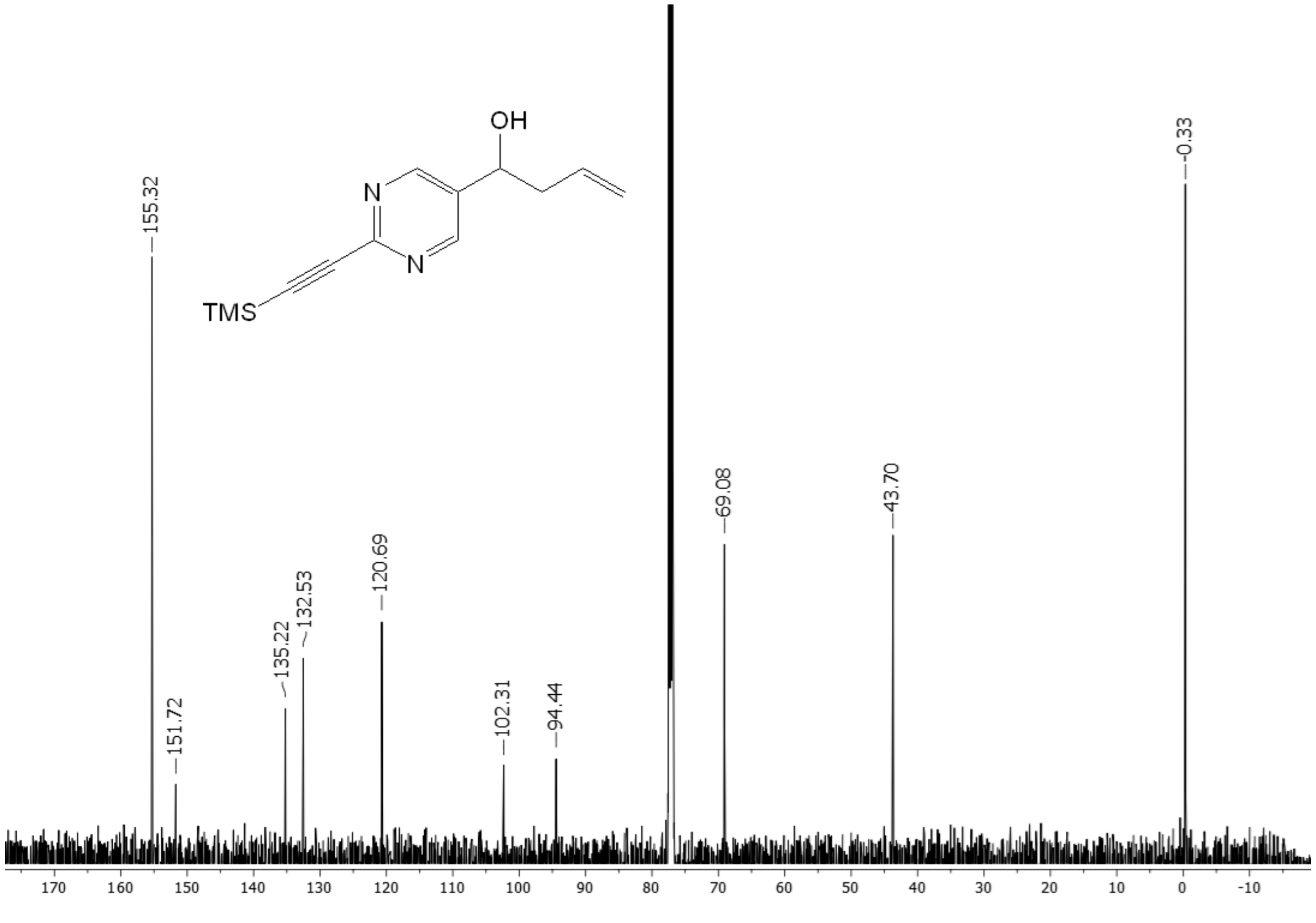

Figure S2. ^{1}H (a) and $^{13}C\{^{1}H\}$ (b) NMR spectra of compound **2c** ($CDCl_3$).

5. HMRS data of alcohols 2b, 2c

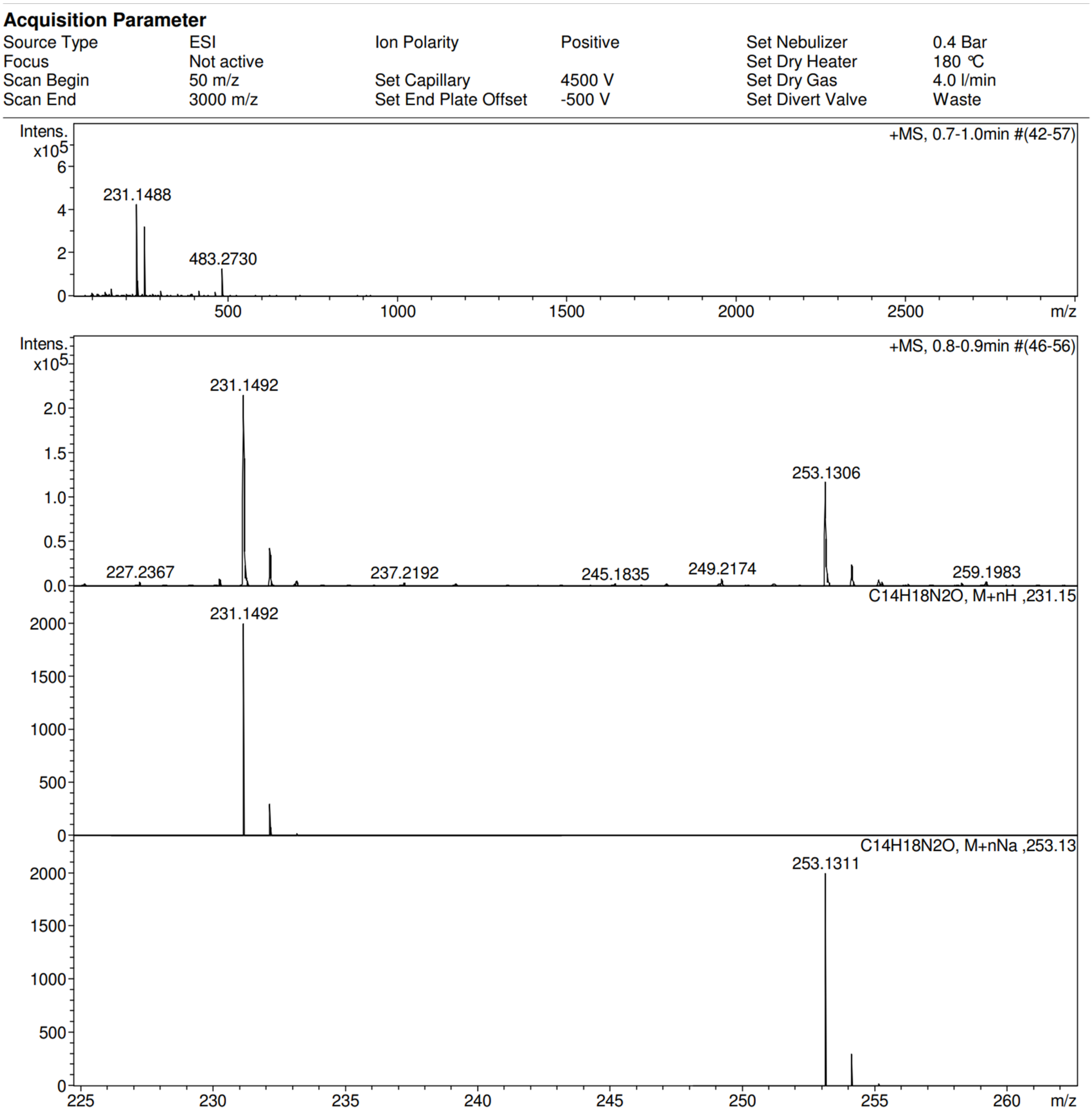

Figure S3. HRMS data of compound **2b**.

Acquisition Parameter

Source Type	ESI	Ion Polarity	Positive	Set Nebulizer	0.4 Bar
Focus	Not active			Set Dry Heater	180 °C
Scan Begin	50 m/z	Set Capillary	4500 V	Set Dry Gas	4.0 l/min
Scan End	3000 m/z	Set End Plate Offset	-500 V	Set Divert Valve	Waste

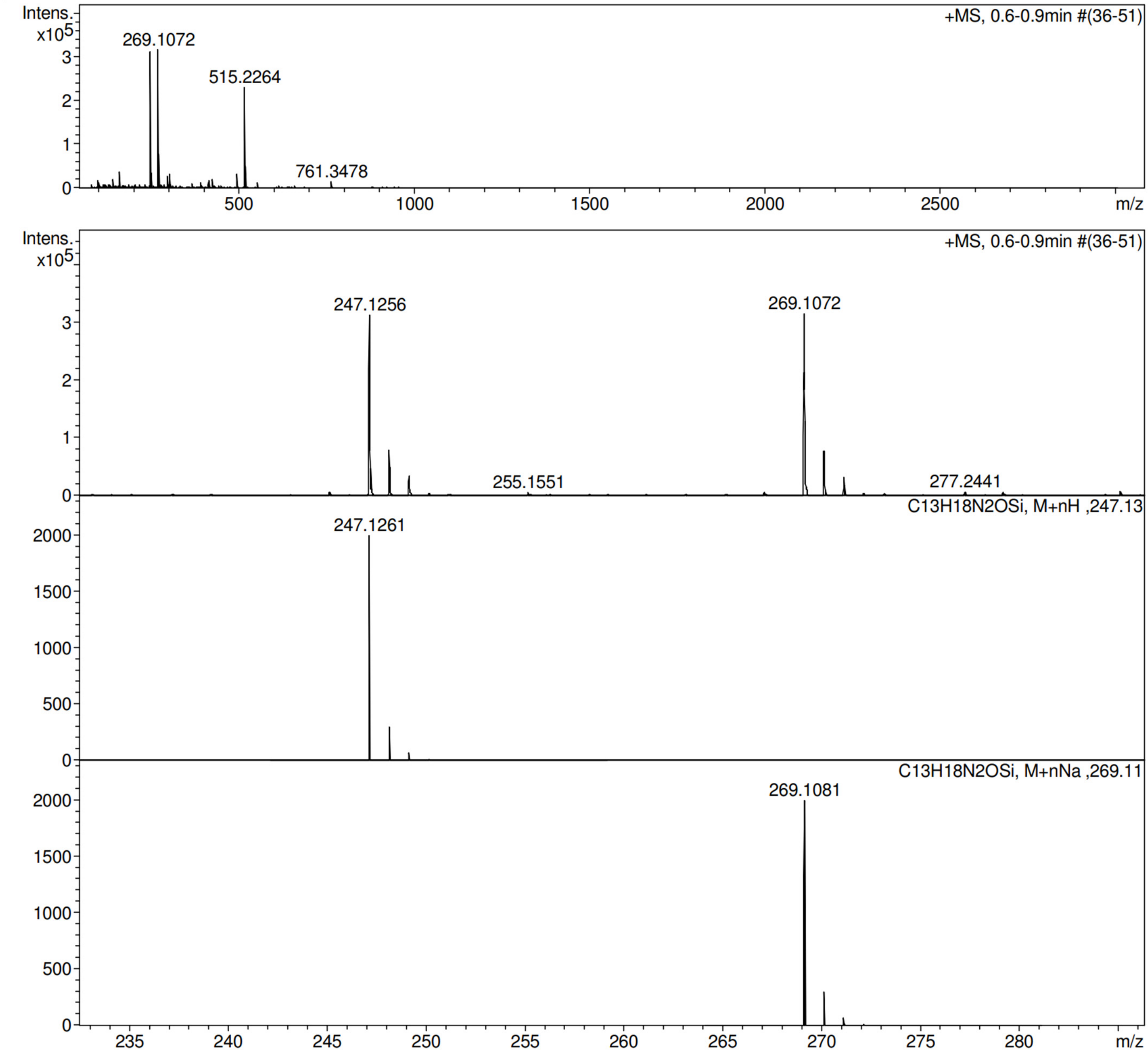

Figure S4. HRMS data of compound **2c**.

6. HPLC data of alcohols 2b, 2c

Conditions: hexane : 2-propanol = 95:5,
Flow rate = 1.0 mL/min, λ = 220 nm, Chiralpak IB.

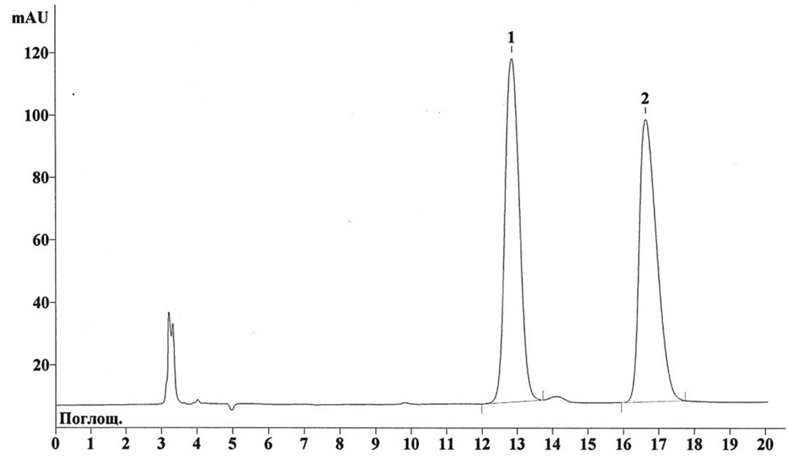

	Peak 1	Peak 2
Retention Time (min)	12.86	16.64
Relative Area (%)	50.49	49.51

Figure S5. HPLC chromatogram for compound **2b** (with triallylborane).

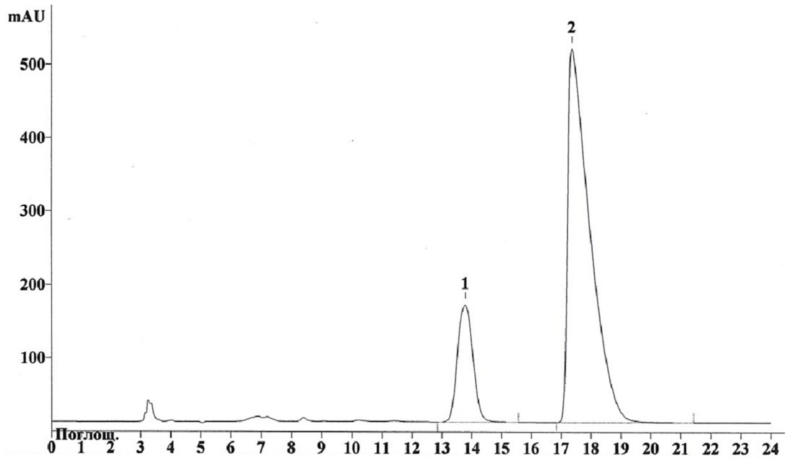

	Peak 1	Peak 2
Retention Time (min)	13.78	17.37
Relative Area (%)	18.03	81.97

Figure S6. HPLC chromatogram for compound **2b** (enantioselective allylboration).

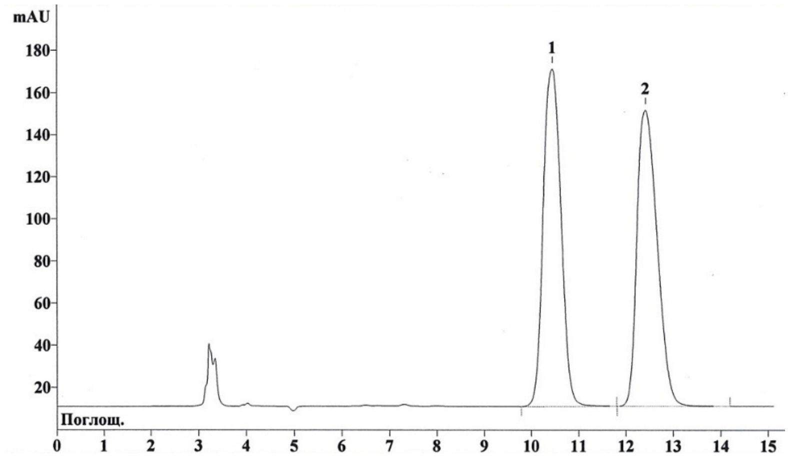

	Peak 1	Peak 2
Retention Time (min)	10.45	12.42
Relative Area (%)	50.14	49.86

Figure S7. HPLC chromatogram for compound **2c** (with triallylborane).

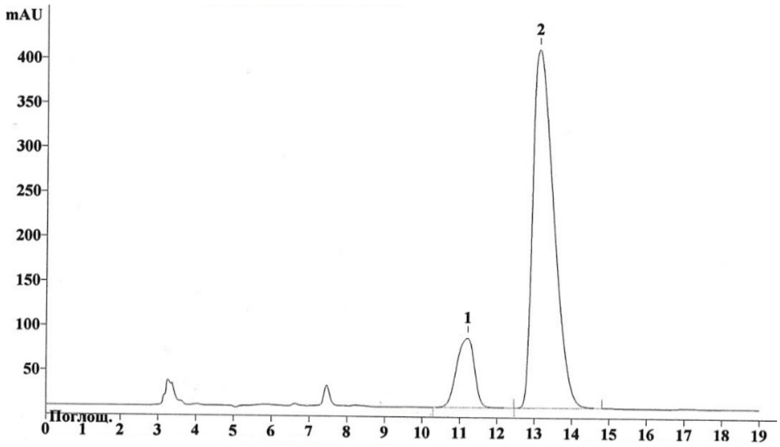

	Peak 1	Peak 2
Retention Time (min)	11.21	13.11
Relative Area (%)	14.08	85.92

Figure S8. HPLC chromatogram for compound **2c** (enantioselective allylboration, starting for Entry 1, Table S1).

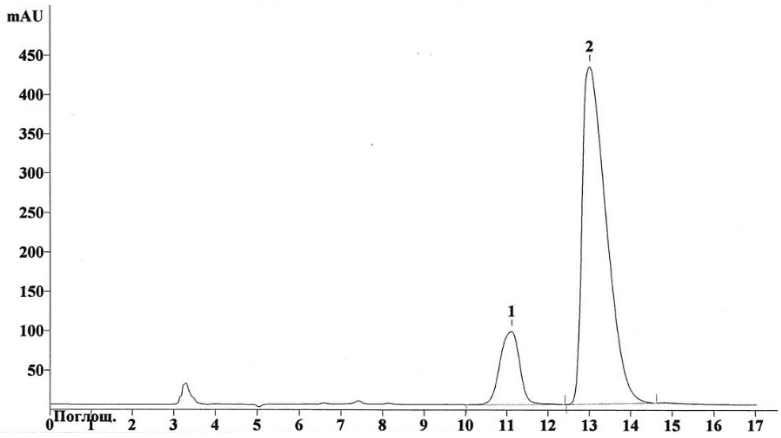

	Peak 1	Peak 2
Retention Time (min)	11.12	13.01
Relative Area (%)	15.18	84.82

Figure S9. HPLC chromatogram for compound **2c** (enantioselective allylboration, starting for Entries 2–4, Table S1).

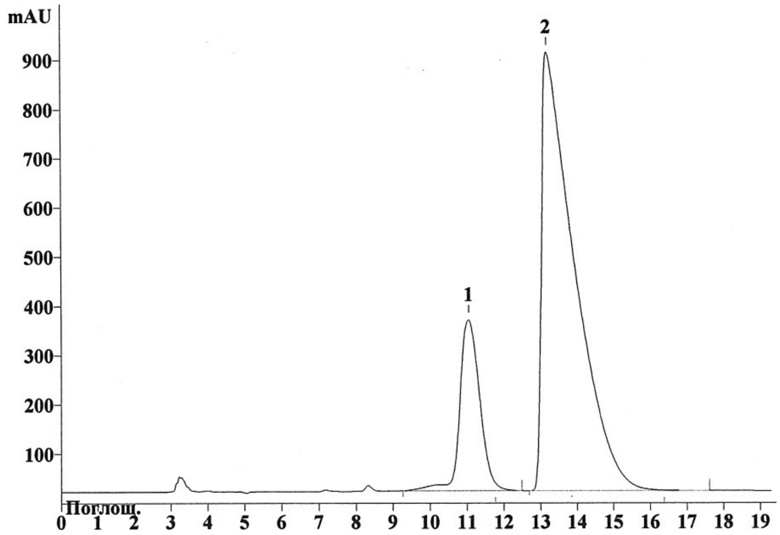

	Peak 1	Peak 2
Retention Time (min)	11.25	13.17
Relative Area (%)	13.59	86.41

Figure S10. HPLC chromatogram for compound **2c** (enantioselective allylboration, starting for Entries 5–10, Table S1).

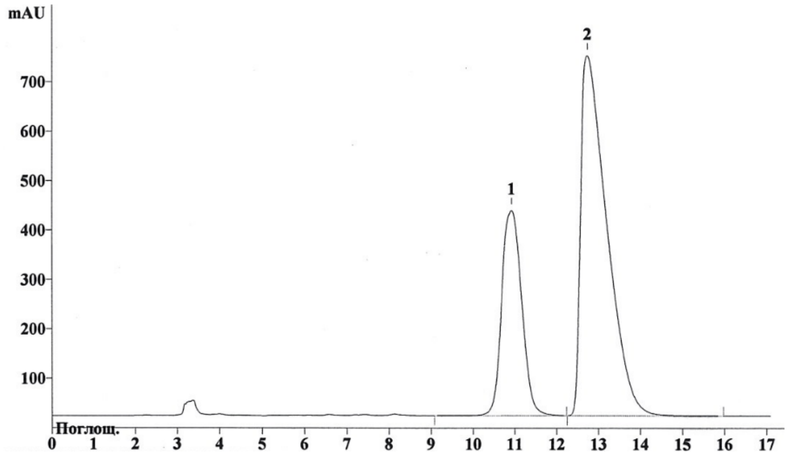

	Peak 1	Peak 2
Retention Time (min)	10.93	12.74
Relative Area (%)	30.73	69.27

Figure S11. HPLC chromatogram for compound **2c** (Table S1, Entry 1).

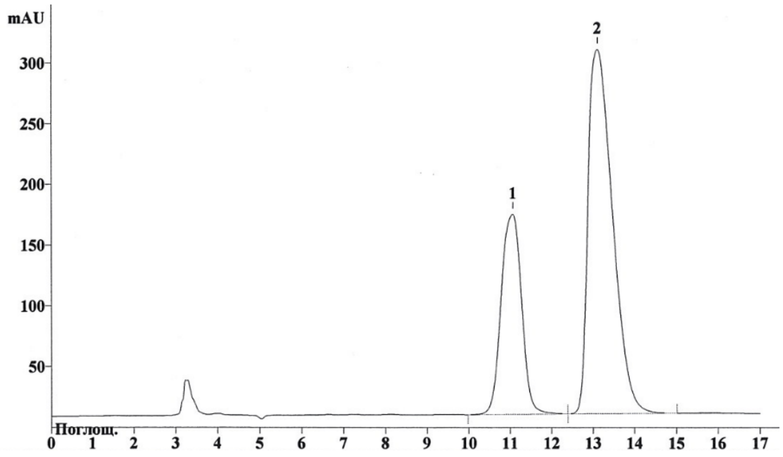

	Peak 1	Peak 2
Retention Time (min)	11.07	13.11
Relative Area (%)	31.78	68.25

Figure S12. HPLC chromatogram for compound **2c** (Table S1, Entry 2).

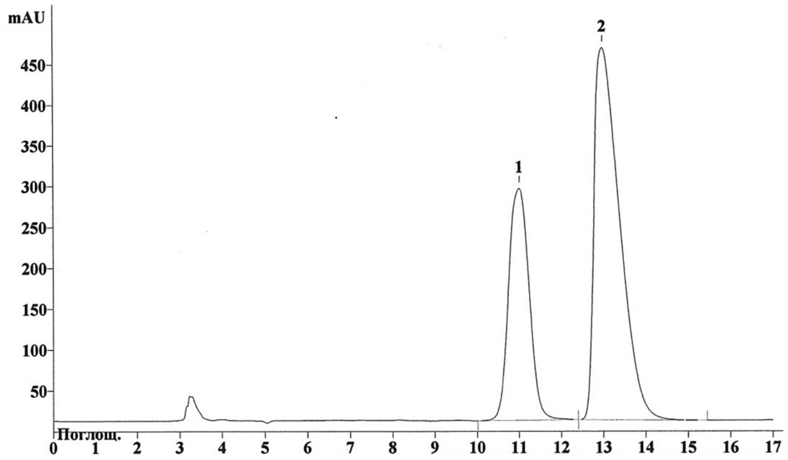

	Peak 1	Peak 2
Retention Time (min)	11.01	12.99
Relative Area (%)	33.65	66.35

Figure S13. HPLC chromatogram for compound **2c** (Table S1, Entry 3).

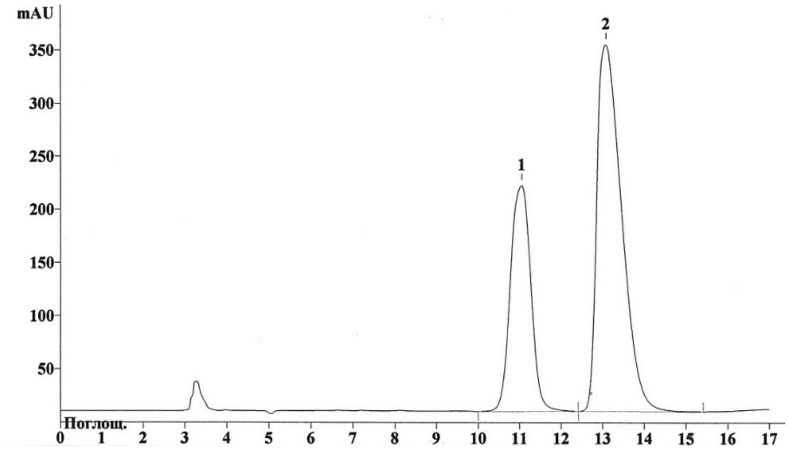

	Peak 1	Peak 2
Retention Time (min)	11.05	13.08
Relative Area (%)	34.05	65.95

Figure S14. HPLC chromatogram for compound **2c** (Table S1, Entry 4).

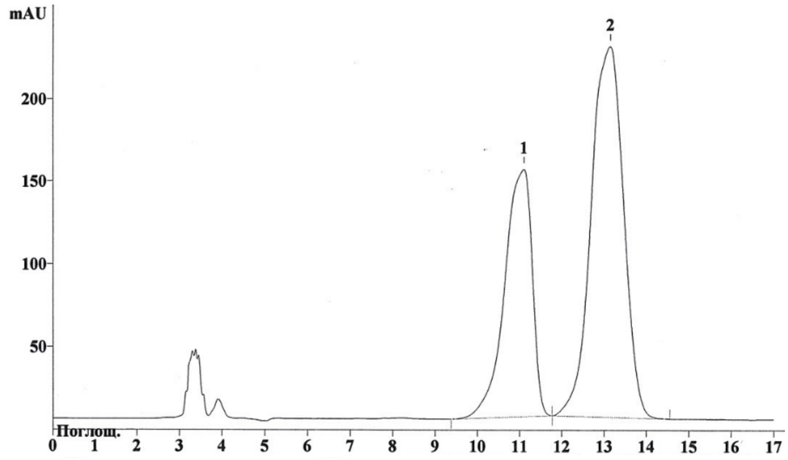

	Peak 1	Peak 2
Retention Time (min)	11.11	13.15
Relative Area (%)	36.48	63.52

Figure S15. HPLC chromatogram for compound **2c** (Table S1, Entry 5).

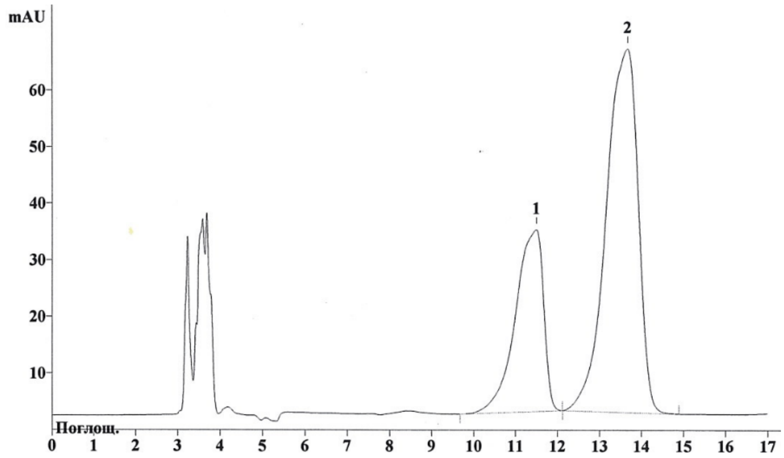

	Peak 1	Peak 2
Retention Time (min)	11.50	13.68
Relative Area (%)	30.68	69.32

Figure S16. HPLC chromatogram for compound **2c** (Table S1, Entry 6).

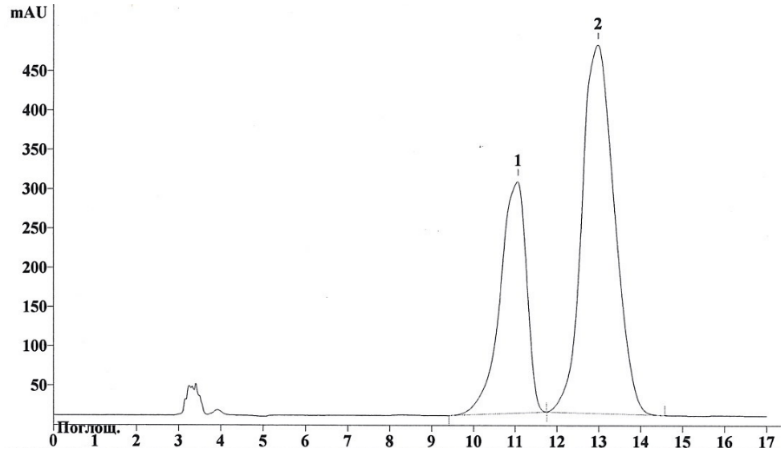

	Peak 1	Peak 2
Retention Time (min)	11.06	12.98
Relative Area (%)	34.05	65.95

Figure S17. HPLC chromatogram for compound **2c** (Table S1, Entry 7).

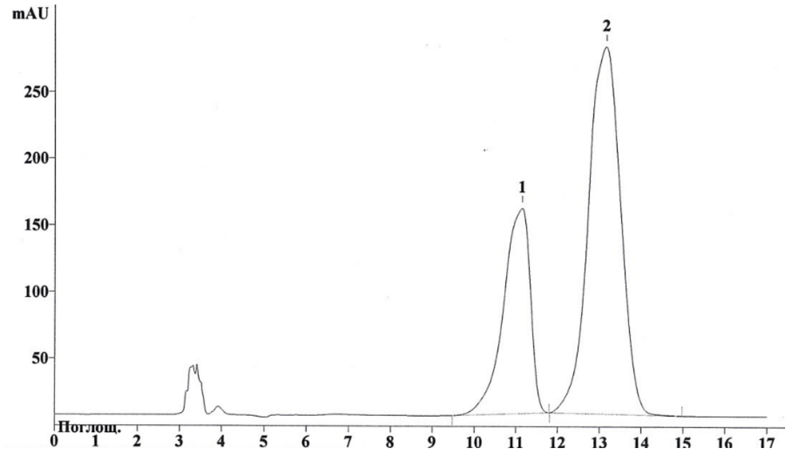

	Peak 1	Peak 2
Retention Time (min)	11.15	13.16
Relative Area (%)	32.02	67.98

Figure S18. HPLC chromatogram for compound **2c** (Table S1, Entry 8).

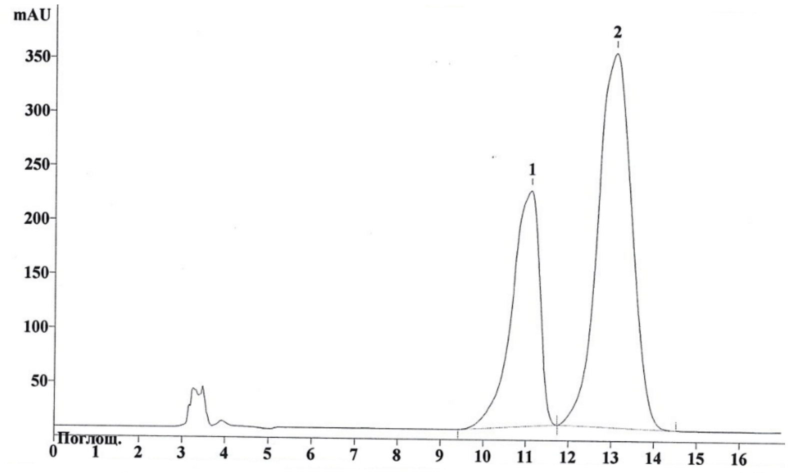

	Peak 1	Peak 2
Retention Time (min)	11.11	13.09
Relative Area (%)	34.18	65.82

Figure S19. HPLC chromatogram for compound **2c** (Table S1, Entry 9).

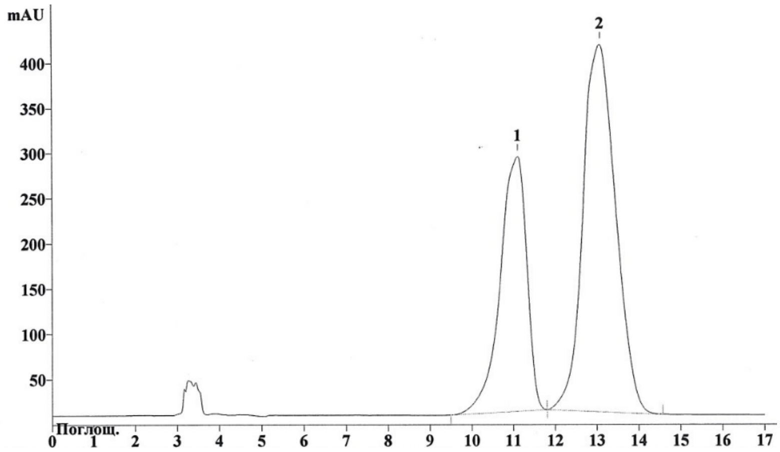

	Peak 1	Peak 2
Retention Time (min)	11.12	13.08
Relative Area (%)	36.31	63.69

Figure S20. HPLC chromatogram for compound **2c** (Table S1, Entry 10).

7. Cartesian coordinates of optimized structures

5a(*RR*)

Sum of electronic and zero-point Energies= -4681.812514
Sum of electronic and thermal Energies= -4681.758403
Sum of electronic and thermal Enthalpies= -4681.757458
Sum of electronic and thermal Free Energies= -4681.906924

Standard orientation:

Center Number	Atomic Number	Atomic Type	Coordinates (Angstroms) X	Y	Z
1	8	0	0.152666	2.014382	-1.553104
2	6	0	-1.226061	2.047251	-1.709021
3	6	0	-2.054974	1.060956	-0.887308
4	6	0	-3.092986	0.311556	-1.407380
5	7	0	-2.075314	0.814376	0.469203
6	1	0	-3.460457	0.189767	-2.422009
7	7	0	-3.032592	-0.015339	0.789276
8	7	0	-3.657868	-0.319632	-0.342320
9	6	0	-4.820239	-1.145098	-0.308785
10	6	0	-4.754025	-2.511914	-0.627240
11	6	0	-6.062143	-0.592880	0.052583
12	6	0	-5.893664	-3.317193	-0.587454
13	6	0	-7.214633	-1.377799	0.102463
14	6	0	-7.109252	-2.733856	-0.220033
15	1	0	-5.828839	-4.376239	-0.834753
16	1	0	-8.170385	-0.937684	0.384334
17	17	0	-8.536506	-3.726595	-0.163766
18	5	0	1.089545	1.035607	-2.085715
19	6	0	2.131613	1.724233	-3.166925
20	1	0	2.899066	0.990796	-3.472659
21	1	0	1.502220	1.916019	-4.060237
22	6	0	2.783205	3.001697	-2.752514
23	6	0	4.101361	3.214786	-2.603170
24	1	0	2.102717	3.834555	-2.530798
25	1	0	4.493205	4.180475	-2.266110
26	1	0	4.837573	2.431808	-2.817809
27	6	0	0.475939	-0.345064	-2.757112
28	1	0	1.191077	-0.712444	-3.514035
29	6	0	0.094242	-1.487171	-1.869146
30	6	0	0.624572	-2.720205	-1.905287
31	1	0	-0.671853	-1.290623	-1.109325
32	1	0	0.306814	-3.502385	-1.208763
33	1	0	1.401095	-2.992224	-2.630046
34	1	0	-0.416359	-0.019462	-3.330615
35	8	0	-0.151759	2.208222	1.117550
36	6	0	1.221879	2.128436	1.285648
37	6	0	1.985288	1.012731	0.565998
38	6	0	3.003501	0.298051	1.168570
39	7	0	2.000079	0.615044	-0.755065
40	1	0	3.368211	0.282315	2.191298
41	7	0	2.938752	-0.268127	-0.977853
42	7	0	3.553368	-0.461007	0.183844
43	6	0	4.697904	-1.309813	0.259769
44	6	0	4.576464	-2.676533	0.562641
45	6	0	5.981038	-0.778417	0.037805
46	6	0	5.700078	-3.503043	0.628149
47	6	0	7.119028	-1.583449	0.098497
48	6	0	6.956970	-2.940317	0.391545
49	1	0	5.593098	-4.562164	0.860070
50	1	0	8.106193	-1.157975	-0.078253
51	17	0	8.364706	-3.958884	0.470155
52	5	0	-1.212272	1.443371	1.750961

53	6	0	-0.811233	0.208236	2.764740
54	1	0	-0.013624	0.614822	3.418682
55	1	0	-1.670575	0.023069	3.432979
56	6	0	-0.357917	-1.093060	2.182975
57	6	0	-1.016397	-2.261871	2.253540
58	1	0	0.587280	-1.081877	1.626623
59	1	0	-0.628188	-3.168722	1.778536
60	1	0	-1.969624	-2.354108	2.787025
61	6	0	-2.297412	2.469871	2.466731
62	1	0	-3.254702	1.948291	2.646267
63	6	0	-1.785939	3.054031	3.743104
64	6	0	-2.263526	2.820505	4.977135
65	1	0	-0.910427	3.711526	3.651335
66	1	0	-1.802083	3.264196	5.865795
67	1	0	-3.136122	2.177687	5.144712
68	1	0	-2.498207	3.287180	1.746587
69	1	0	1.473847	1.955546	2.350143
70	6	0	1.847138	3.488488	0.878210
71	1	0	1.573085	3.646402	-0.177032
72	1	0	2.944666	3.417282	0.935340
73	6	0	1.356651	4.635257	1.711993
74	6	0	2.134952	5.426607	2.462634
75	1	0	0.277151	4.820813	1.681316
76	1	0	1.718063	6.256780	3.041699
77	1	0	3.220026	5.281095	2.521119
78	1	0	-1.507970	1.800840	-2.751805
79	6	0	-1.723977	3.488863	-1.432990
80	1	0	-2.825694	3.500060	-1.421103
81	1	0	-1.365222	3.748270	-0.423441
82	6	0	-1.210639	4.479219	-2.436819
83	6	0	-1.978378	5.268580	-3.199537
84	1	0	-0.120679	4.538001	-2.533431
85	1	0	-1.541992	5.976554	-3.911221
86	1	0	-3.072685	5.244956	-3.136552
87	17	0	6.155924	0.912096	-0.309724
88	17	0	3.011780	-3.354673	0.872213
89	17	0	-6.168100	1.096937	0.433718
90	17	0	-3.234796	-3.215125	-1.078437

6a

Sum of electronic and zero-point Energies= -6646.322839
Sum of electronic and thermal Energies= -6646.251570
Sum of electronic and thermal Enthalpies= -6646.250626
Sum of electronic and thermal Free Energies= -6646.446396

Standard orientation:

Center Number	Atomic Number	Atomic Type	X	Y	Z
1	8	0	1.284315	-0.156792	1.716034
2	6	0	0.129883	0.076683	2.493144
3	6	0	-1.042219	0.616155	1.697043
4	6	0	-1.640406	1.859107	1.776734
5	7	0	-1.739910	-0.113820	0.772213
6	1	0	-1.428509	2.745242	2.368079
7	7	0	-2.708676	0.593595	0.277727
8	7	0	-2.665325	1.806594	0.881070
9	6	0	-3.605107	2.818187	0.552826
10	6	0	-3.425218	3.625999	-0.584842
11	6	0	-4.742067	3.035149	1.351526
12	6	0	-4.343482	4.622366	-0.921774
13	6	0	-5.673101	4.027752	1.041425
14	6	0	-5.455652	4.808719	-0.097065
15	1	0	-4.189059	5.238484	-1.806833
16	1	0	-6.547987	4.181628	1.671948
17	17	0	-6.606356	6.051376	-0.501236
18	5	0	2.168937	0.945609	1.335363
19	6	0	2.719407	1.846701	2.606831
20	1	0	3.360419	2.664832	2.237696
21	1	0	1.822395	2.334204	3.038868
22	6	0	3.424762	1.084322	3.681239
23	6	0	4.719535	1.197219	4.021658
24	1	0	2.820712	0.338633	4.215817
25	1	0	5.165730	0.570758	4.801491
26	1	0	5.380679	1.924184	3.536307
27	6	0	1.615305	1.941844	0.142697
28	1	0	2.388650	2.694619	-0.095688
29	6	0	1.134922	1.289169	-1.113411
30	6	0	1.583087	1.533738	-2.356203
31	1	0	0.352929	0.530793	-0.987212
32	1	0	1.186114	1.000328	-3.226555
33	1	0	2.357702	2.284592	-2.551099
34	1	0	0.772768	2.500876	0.594549
35	8	0	1.943479	-2.244475	-0.570411
36	6	0	3.160016	-2.333202	0.160272
37	6	0	3.888158	-1.009991	0.146606
38	6	0	5.160858	-0.855925	-0.363601
39	7	0	3.537635	0.220658	0.663678
40	1	0	5.826190	-1.551282	-0.866288
41	7	0	4.508647	1.080742	0.497535
42	7	0	5.491555	0.437500	-0.119327
43	6	0	6.714998	1.111148	-0.410901
44	6	0	6.832971	1.921559	-1.552925
45	6	0	7.824327	0.973496	0.441296
46	6	0	8.022417	2.593385	-1.839383
47	6	0	9.024951	1.632318	0.173885
48	6	0	9.102189	2.437686	-0.965926
49	1	0	8.101181	3.220582	-2.726432
50	1	0	9.876322	1.520464	0.843899
51	17	0	10.590617	3.269044	-1.309692
52	5	0	1.604291	-3.021728	-1.642106
53	6	0	2.519271	-4.214936	-2.190793
54	1	0	3.115696	-4.701739	-1.401590

55	1	0	1.836343	-4.980776	-2.604483
56	6	0	3.417131	-3.732697	-3.299351
57	6	0	4.758380	-3.768929	-3.301282
58	1	0	2.906996	-3.306404	-4.173580
59	1	0	5.336995	-3.387916	-4.148766
60	1	0	5.326692	-4.198848	-2.468029
61	6	0	0.253311	-2.580583	-2.367341
62	1	0	0.531722	-1.734262	-3.026570
63	6	0	-0.463503	-3.630621	-3.162336
64	6	0	-0.601195	-3.659862	-4.496271
65	1	0	-0.903997	-4.451971	-2.580113
66	1	0	-1.130997	-4.474636	-5.000106
67	1	0	-0.192086	-2.867931	-5.134932
68	1	0	-0.416692	-2.153339	-1.599410
69	1	0	3.845560	-3.027595	-0.349151
70	6	0	2.920380	-2.854613	1.588279
71	1	0	2.191996	-2.168467	2.044277
72	1	0	3.869566	-2.765398	2.140733
73	6	0	2.426224	-4.272403	1.636786
74	6	0	3.111966	-5.295211	2.165482
75	1	0	1.424261	-4.457906	1.232934
76	1	0	2.695675	-6.307158	2.193301
77	1	0	4.108523	-5.159855	2.602309
78	1	0	0.335225	0.833530	3.272640
79	6	0	-0.299780	-1.227916	3.206165
80	1	0	-1.321114	-1.090614	3.596147
81	1	0	-0.347992	-2.018989	2.437971
82	6	0	0.604942	-1.639424	4.331953
83	6	0	0.223854	-1.762104	5.610632
84	1	0	1.648383	-1.848791	4.071955
85	1	0	0.927682	-2.067768	6.391226
86	1	0	-0.808571	-1.570027	5.925534
87	17	0	7.704578	-0.032765	1.850151
88	17	0	5.486560	2.079224	-2.632711
89	17	0	-5.001157	2.041113	2.753695
90	17	0	-2.029456	3.385928	-1.586084
91	6	0	-2.099069	-5.195226	0.759938
92	6	0	-3.268558	-4.425676	0.330523
93	6	0	-3.439893	-3.054569	0.178211
94	7	0	-4.435321	-5.048548	-0.032431
95	1	0	-2.785422	-2.197841	0.350356
96	7	0	-5.302501	-4.172123	-0.395936
97	7	0	-4.709217	-2.940109	-0.271579
98	6	0	-5.432763	-1.771695	-0.631575
99	6	0	-5.240445	-1.166170	-1.885394
100	6	0	-6.380761	-1.218830	0.245522
101	6	0	-5.969427	-0.037800	-2.263313
102	6	0	-7.126843	-0.094161	-0.109596
103	6	0	-6.905824	0.479009	-1.364512
104	1	0	-5.809042	0.421860	-3.237660
105	1	0	-7.857827	0.323259	0.581461
106	17	0	-4.081880	-1.837253	-2.989137
107	17	0	-6.625800	-1.942756	1.804023
108	17	0	-7.828288	1.884651	-1.823097
109	1	0	-2.271968	-6.297113	0.779286
110	8	0	-1.025752	-4.706686	1.064500

7a

Sum of electronic and zero-point Energies= -6646.324181
Sum of electronic and thermal Energies= -6646.253275
Sum of electronic and thermal Enthalpies= -6646.252331
Sum of electronic and thermal Free Energies= -6646.446748

Standard orientation:

Center Number	Atomic Number	Atomic Type	Coordinates (Angstroms) X	Y	Z
1	8	0	3.390225	0.745403	1.122151
2	6	0	4.684756	0.966702	1.627937
3	6	0	5.761036	0.516022	0.664417
4	6	0	6.786077	-0.392195	0.844948
5	7	0	5.880475	1.026086	-0.600160
6	1	0	7.079654	-1.023211	1.678859
7	7	0	6.895821	0.495800	-1.205143
8	7	0	7.466275	-0.376490	-0.336167
9	6	0	8.627195	-1.102551	-0.706119
10	6	0	8.526499	-2.340023	-1.366345
11	6	0	9.909503	-0.587352	-0.445454
12	6	0	9.663000	-3.050963	-1.756306
13	6	0	11.063243	-1.276318	-0.822843
14	6	0	10.917551	-2.503096	-1.476313
15	1	0	9.567465	-4.007923	-2.267786
16	1	0	12.049039	-0.862038	-0.614184
17	17	0	12.345172	-3.376491	-1.958384
18	5	0	2.702066	-0.537951	1.284195
19	6	0	2.713912	-1.126142	2.825485
20	1	0	2.137319	-2.066275	2.870651
21	1	0	3.766362	-1.405626	3.034882
22	6	0	2.236862	-0.180743	3.880850
23	6	0	1.172981	-0.353573	4.681700
24	1	0	2.800738	0.757492	3.979659
25	1	0	0.868516	0.408619	5.406886
26	1	0	0.566242	-1.264987	4.640574
27	6	0	3.128179	-1.727812	0.223565
28	1	0	2.482583	-2.608766	0.392033
29	6	0	3.117876	-1.354053	-1.222898
30	6	0	2.280730	-1.827110	-2.161665
31	1	0	3.855457	-0.599370	-1.525875
32	1	0	2.321179	-1.473453	-3.197529
33	1	0	1.527704	-2.588914	-1.929276
34	1	0	4.154002	-2.031758	0.509670
35	8	0	0.137121	3.346685	0.158833
36	6	0	1.127259	2.310455	0.141685
37	6	0	0.478609	0.997053	0.525055
38	6	0	-0.869900	0.693600	0.523859
39	7	0	1.119509	-0.149388	0.940179
40	1	0	-1.766197	1.278854	0.333328
41	7	0	0.264714	-1.113926	1.158895
42	7	0	-0.935917	-0.609375	0.909538
43	6	0	-2.090630	-1.443727	0.999213
44	6	0	-2.493069	-2.210443	-0.107348
45	6	0	-2.855826	-1.500745	2.174783
46	6	0	-3.612971	-3.039252	-0.045005
47	6	0	-3.982731	-2.319970	2.261922
48	6	0	-4.340252	-3.081624	1.146839
49	1	0	-3.913841	-3.627246	-0.911020
50	1	0	-4.567254	-2.356198	3.180228
51	17	0	-5.742722	-4.107902	1.240756
52	5	0	-0.795031	3.742912	-0.759957
53	6	0	-1.098483	2.982270	-2.135755
54	1	0	-0.393381	3.441386	-2.861813

55	1	0	-0.832203	1.914541	-2.092635
56	6	0	-2.495995	3.138985	-2.667441
57	6	0	-3.367390	2.140873	-2.871649
58	1	0	-2.815862	4.163947	-2.897316
59	1	0	-4.376569	2.330384	-3.251107
60	1	0	-3.110118	1.096787	-2.662837
61	6	0	-1.510565	5.123969	-0.386503
62	1	0	-1.323953	5.360348	0.673024
63	6	0	-0.985517	6.225728	-1.269142
64	6	0	-0.132850	7.192300	-0.896216
65	1	0	-1.315204	6.204588	-2.317077
66	1	0	0.222958	7.944299	-1.607594
67	1	0	0.238025	7.268312	0.132248
68	1	0	-2.601491	5.027460	-0.530865
69	6	0	4.818080	2.473659	1.953562
70	1	0	4.637286	3.027727	1.016333
71	1	0	4.010298	2.732329	2.655991
72	1	0	4.845222	0.404347	2.565344
73	6	0	6.151339	2.861653	2.526961
74	6	0	6.350374	3.315622	3.771458
75	1	0	7.016364	2.754854	1.859505
76	1	0	7.348874	3.584785	4.130459
77	1	0	5.521240	3.444008	4.476934
78	17	0	6.957105	-2.999639	-1.705265
79	17	0	10.063183	0.943084	0.362659
80	17	0	-1.592360	-2.113553	-1.587634
81	17	0	-2.406606	-0.530949	3.540535
82	8	0	-3.430872	2.667781	1.185081
83	6	0	-4.648009	2.670587	1.172996
84	1	0	-5.225648	3.502651	1.637865
85	6	0	-5.472934	1.619941	0.567766
86	6	0	-6.856689	1.549545	0.488218
87	7	0	-4.957112	0.503104	-0.040199
88	1	0	-7.651717	2.207712	0.829105
89	7	0	-5.912567	-0.235059	-0.478980
90	7	0	-7.092292	0.390280	-0.166056
91	6	0	-8.332419	-0.202410	-0.525181
92	6	0	-9.090689	-0.915555	0.420100
93	6	0	-8.809995	-0.114896	-1.844779
94	6	0	-10.294640	-1.527687	0.070039
95	6	0	-10.009374	-0.720931	-2.221647
96	6	0	-10.734004	-1.420906	-1.252743
97	1	0	-10.870877	-2.077838	0.813087
98	1	0	-10.367090	-0.645297	-3.247730
99	17	0	-7.899043	0.773528	-3.025257
100	17	0	-8.522055	-1.035902	2.056996
101	17	0	-12.231464	-2.181298	-1.706430
102	1	0	1.831522	2.573744	0.940964
103	6	0	1.930324	2.247748	-1.176555
104	1	0	2.746446	1.532713	-1.010194
105	1	0	1.291547	1.842546	-1.977382
106	6	0	2.492483	3.582440	-1.589111
107	6	0	3.790966	3.820167	-1.817905
108	1	0	1.777142	4.403862	-1.721930
109	1	0	4.134882	4.809739	-2.136398
110	1	0	4.550054	3.039512	-1.696398

TS1a(*S*)

Sum of electronic and zero-point Energies= -6646.320024
Sum of electronic and thermal Energies= -6646.250323
Sum of electronic and thermal Enthalpies= -6646.249379
Sum of electronic and thermal Free Energies= -6646.440418

Standard orientation:

Center Number	Atomic Number	Atomic Type	Coordinates (Angstroms) X	Y	Z
1	8	0	3.185455	0.604010	0.964389
2	6	0	4.402141	0.949346	1.580184
3	6	0	5.601026	0.616504	0.718102
4	6	0	6.693583	-0.180644	0.998952
5	7	0	5.784618	1.141614	-0.532904
6	1	0	6.971704	-0.780619	1.860648
7	7	0	6.901782	0.722284	-1.036924
8	7	0	7.476380	-0.089523	-0.113277
9	6	0	8.736793	-0.688488	-0.365479
10	6	0	8.829915	-1.939872	-1.000287
11	6	0	9.927870	-0.033098	-0.005631
12	6	0	10.066636	-2.530796	-1.266443
13	6	0	11.177987	-0.599698	-0.259158
14	6	0	11.224768	-1.846872	-0.887814
15	1	0	10.120893	-3.500803	-1.758893
16	1	0	12.090073	-0.076815	0.025980
17	17	0	12.775929	-2.571092	-1.209898
18	5	0	2.620964	-0.743552	1.070251
19	6	0	2.562360	-1.347755	2.606049
20	1	0	2.078017	-2.339719	2.590894
21	1	0	3.613841	-1.525975	2.909043
22	6	0	1.898635	-0.469704	3.617266
23	6	0	0.773595	-0.747581	4.295948
24	1	0	2.366473	0.510106	3.788122
25	1	0	0.329255	-0.028754	4.992677
26	1	0	0.254369	-1.705210	4.177824
27	6	0	3.267067	-1.861997	0.044499
28	1	0	2.725721	-2.819567	0.148762
29	6	0	3.331600	-1.461471	-1.393598
30	6	0	2.672129	-2.039659	-2.411567
31	1	0	3.964332	-0.592564	-1.617364
32	1	0	2.751464	-1.660506	-3.436192
33	1	0	2.031256	-2.915404	-2.257576
34	1	0	4.296041	-2.042272	0.413058
35	8	0	-0.280553	2.913095	0.197802
36	6	0	0.777713	1.982885	0.018804
37	6	0	0.286797	0.560399	0.212447
38	6	0	-0.994423	0.079637	0.031901
39	7	0	1.039315	-0.528264	0.599045
40	1	0	-1.927495	0.575698	-0.215480
41	7	0	0.309573	-1.612948	0.653229
42	7	0	-0.919369	-1.248193	0.311943
43	6	0	-1.973552	-2.208580	0.267280
44	6	0	-2.179037	-2.989241	-0.882717
45	6	0	-2.846065	-2.369234	1.356661
46	6	0	-3.210451	-3.927313	-0.945446
47	6	0	-3.888683	-3.295681	1.316404
48	6	0	-4.050532	-4.065802	0.162252
49	1	0	-3.354548	-4.527954	-1.842508
50	1	0	-4.558697	-3.407107	2.167651
51	17	0	-5.344320	-5.229030	0.099797
52	5	0	-1.267425	3.489879	-0.604510
53	6	0	-1.403221	3.166098	-2.194900
54	1	0	-0.518221	3.640549	-2.661548

55	1	0	-1.320431	2.083552	-2.387431
56	6	0	-2.629782	3.715484	-2.849636
57	6	0	-3.665788	3.000150	-3.322173
58	1	0	-2.688087	4.809988	-2.923917
59	1	0	-4.537049	3.484408	-3.775236
60	1	0	-3.667429	1.904206	-3.298165
61	6	0	-1.722789	4.940219	-0.044666
62	1	0	-1.734088	4.903337	1.058183
63	6	0	-0.803432	6.032257	-0.509761
64	6	0	0.099435	6.683207	0.239737
65	1	0	-0.868197	6.294988	-1.575470
66	1	0	0.753990	7.448762	-0.189150
67	1	0	0.222369	6.469551	1.307679
68	1	0	-2.747694	5.180901	-0.384485
69	6	0	4.356486	2.459585	1.915408
70	1	0	4.201435	2.999300	0.965468
71	1	0	3.469747	2.631957	2.545368
72	1	0	4.536516	0.398377	2.528672
73	6	0	5.589723	2.974172	2.601456
74	6	0	5.634664	3.436127	3.858192
75	1	0	6.515282	2.959964	2.011288
76	1	0	6.566391	3.801496	4.301813
77	1	0	4.739183	3.475193	4.489281
78	17	0	7.377274	-2.769282	-1.464127
79	17	0	9.844379	1.518364	0.771942
80	17	0	-1.146846	-2.772827	-2.260013
81	17	0	-2.636273	-1.395575	2.776148
82	8	0	-2.850701	2.432484	-0.090593
83	6	0	-4.040224	2.731534	-0.206523
84	1	0	-4.359783	3.785472	-0.302872
85	6	0	-5.092281	1.727925	-0.171674
86	6	0	-6.470073	1.905785	-0.172824
87	7	0	-4.837991	0.379300	-0.123955

88	1	0	-7.101411	2.790259	-0.199232
89	7	0	-5.943472	-0.269951	-0.096805
90	7	0	-6.963183	0.650349	-0.122705
91	6	0	-8.317971	0.219699	-0.094970
92	6	0	-8.921391	-0.151516	1.119591
93	6	0	-9.069870	0.128250	-1.279399
94	6	0	-10.241130	-0.602621	1.162570
95	6	0	-10.392832	-0.315109	-1.262363
96	6	0	-10.957590	-0.676088	-0.035528
97	1	0	-10.697245	-0.887009	2.110173
98	1	0	-10.965023	-0.380727	-2.186973
99	17	0	-8.340637	0.572312	-2.791145
100	17	0	-8.010533	-0.035670	2.591884
101	17	0	-12.604548	-1.233385	0.001082
102	1	0	1.485070	2.180893	0.834114
103	6	0	1.565235	2.139017	-1.302560
104	1	0	2.389977	1.413556	-1.269965
105	1	0	0.914575	1.859702	-2.147757
106	6	0	2.113442	3.525615	-1.503545
107	6	0	3.396684	3.808481	-1.764677
108	1	0	1.397564	4.352583	-1.427514
109	1	0	3.727418	4.841780	-1.913072
110	1	0	4.161318	3.027242	-1.829489

8a(*S*)

Sum of electronic and zero-point Energies= -6646.320040
Sum of electronic and thermal Energies= -6646.250140
Sum of electronic and thermal Enthalpies= -6646.249196
Sum of electronic and thermal Free Energies= -6646.439895

Standard orientation:

Center Number	Atomic Number	Atomic Type	Coordinates (Angstroms) X	Y	Z
1	8	0	3.131029	0.618505	0.927922
2	6	0	4.339334	0.987175	1.546545
3	6	0	5.549257	0.641296	0.704917
4	6	0	6.640000	-0.148954	1.011445
5	7	0	5.746617	1.146508	-0.552244
6	1	0	6.909424	-0.734074	1.886029
7	7	0	6.870117	0.721221	-1.036437
8	7	0	7.435627	-0.074350	-0.092997
9	6	0	8.699868	-0.674217	-0.322222
10	6	0	8.802350	-1.932802	-0.941147
11	6	0	9.885695	-0.011839	0.042113
12	6	0	10.043067	-2.523755	-1.187973
13	6	0	11.139675	-0.578357	-0.191951
14	6	0	11.195699	-1.832459	-0.805937
15	1	0	10.104519	-3.499260	-1.668603
16	1	0	12.047621	-0.049945	0.096212
17	17	0	12.751814	-2.556002	-1.105410
18	5	0	2.568234	-0.725359	1.073925
19	6	0	2.487004	-1.276385	2.629671
20	1	0	2.000458	-2.267404	2.638777
21	1	0	3.532651	-1.445453	2.956656
22	6	0	1.806955	-0.362656	3.597306
23	6	0	0.656523	-0.604063	4.247084
24	1	0	2.284411	0.614492	3.756272
25	1	0	0.202352	0.140799	4.909222
26	1	0	0.125361	-1.556340	4.138492
27	6	0	3.237080	-1.873971	0.096864
28	1	0	2.702584	-2.832884	0.221384
29	6	0	3.324193	-1.516792	-1.351531
30	6	0	2.700847	-2.140231	-2.365496
31	1	0	3.941468	-0.640224	-1.588092
32	1	0	2.794667	-1.790147	-3.399208
33	1	0	2.077135	-3.025951	-2.198938
34	1	0	4.261331	-2.033493	0.488009
35	8	0	-0.351071	2.889267	0.159493
36	6	0	0.700574	1.968402	-0.031671
37	6	0	0.228882	0.536008	0.163259
38	6	0	-1.041402	0.030912	-0.031162
39	7	0	0.991389	-0.537206	0.574743
40	1	0	-1.978759	0.501129	-0.306995
41	7	0	0.277491	-1.632558	0.634840
42	7	0	-0.951278	-1.291682	0.269729
43	6	0	-1.991555	-2.266916	0.230153
44	6	0	-2.137400	-3.110425	-0.884222
45	6	0	-2.908950	-2.382839	1.288302
46	6	0	-3.153876	-4.065078	-0.940994
47	6	0	-3.939253	-3.323344	1.251623
48	6	0	-4.041117	-4.155394	0.134229
49	1	0	-3.250752	-4.715104	-1.809470
50	1	0	-4.645558	-3.398380	2.077247
51	17	0	-5.318688	-5.336640	0.075925
52	5	0	-1.421411	3.436185	-0.603996
53	6	0	-1.474410	3.208389	-2.251855
54	1	0	-0.604285	3.753791	-2.656470

55	1	0	-1.346623	2.139368	-2.487283
56	6	0	-2.724872	3.728702	-2.857101
57	6	0	-3.803768	2.990987	-3.197007
58	1	0	-2.797631	4.818852	-2.970147
59	1	0	-4.712042	3.454110	-3.595990
60	1	0	-3.791289	1.896666	-3.142287
61	6	0	-1.767428	4.944160	-0.071865
62	1	0	-1.797089	4.915047	1.032173
63	6	0	-0.789673	5.983956	-0.526037
64	6	0	0.112421	6.617712	0.239217
65	1	0	-0.811449	6.229363	-1.597796
66	1	0	0.810231	7.348023	-0.183049
67	1	0	0.193943	6.421088	1.314425
68	1	0	-2.773275	5.251188	-0.418941
69	6	0	4.282382	2.505382	1.841625
70	1	0	4.134110	3.018327	0.875935
71	1	0	3.388525	2.689934	2.457812
72	1	0	4.467701	0.461137	2.509938
73	6	0	5.506276	3.044592	2.525346
74	6	0	5.536992	3.540759	3.769446
75	1	0	6.437543	3.019384	1.944550
76	1	0	6.462617	3.922991	4.211680
77	1	0	4.635321	3.592341	4.390749
78	17	0	7.356212	-2.770704	-1.409855
79	17	0	9.790514	1.548751	0.799774
80	17	0	-1.050519	-2.952292	-2.226281
81	17	0	-2.771189	-1.336609	2.664535
82	8	0	-2.753318	2.502573	-0.195613
83	6	0	-3.956340	2.809079	-0.323514
84	1	0	-4.270781	3.859948	-0.403114
85	6	0	-4.986257	1.795103	-0.246976
86	6	0	-6.366174	1.952643	-0.198367
87	7	0	-4.710317	0.450164	-0.208358
88	1	0	-7.011700	2.827180	-0.203673
89	7	0	-5.804882	-0.215009	-0.141291
90	7	0	-6.837295	0.689654	-0.130062
91	6	0	-8.185405	0.241576	-0.066666
92	6	0	-8.753327	-0.138775	1.161930
93	6	0	-8.966197	0.146683	-1.232003
94	6	0	-10.068716	-0.598591	1.237249
95	6	0	-10.284828	-0.306381	-1.182547
96	6	0	-10.815738	-0.672530	0.057847
97	1	0	-10.498841	-0.889602	2.194961
98	1	0	-10.878913	-0.374291	-2.093086
99	17	0	-8.278817	0.598745	-2.760970
100	17	0	-7.803404	-0.023885	2.609419
101	17	0	-12.458679	-1.236549	0.135938
102	1	0	1.419902	2.161537	0.775015
103	6	0	1.480402	2.122132	-1.359928
104	1	0	2.298876	1.388700	-1.343548
105	1	0	0.818466	1.856462	-2.200962
106	6	0	2.037790	3.505087	-1.556051
107	6	0	3.313906	3.780002	-1.857817
108	1	0	1.333100	4.335656	-1.433406
109	1	0	3.651449	4.812437	-1.997116
110	1	0	4.067644	2.992757	-1.963922

TS2a(*S*)

Sum of electronic and zero-point Energies= -6646.317913
Sum of electronic and thermal Energies= -6646.249377
Sum of electronic and thermal Enthalpies= -6646.248433
Sum of electronic and thermal Free Energies= -6646.434820

Standard orientation:

Center Number	Atomic Number	Atomic Type	Coordinates (Angstroms) X	Y	Z
1	8	0	3.077316	0.653928	0.794798
2	6	0	4.258480	1.022496	1.463961
3	6	0	5.503288	0.653309	0.685996
4	6	0	6.561769	-0.157018	1.047499
5	7	0	5.773250	1.152844	-0.559981
6	1	0	6.775711	-0.745810	1.934859
7	7	0	6.911089	0.705212	-0.986880
8	7	0	7.413042	-0.099760	-0.015663
9	6	0	8.672988	-0.728314	-0.181836
10	6	0	8.786228	-1.948149	-0.872567
11	6	0	9.845388	-0.134774	0.318998
12	6	0	10.023920	-2.567102	-1.058586
13	6	0	11.095668	-0.731754	0.150162
14	6	0	11.162518	-1.945228	-0.539717
15	1	0	10.093486	-3.511644	-1.596616
16	1	0	11.992558	-0.256296	0.545142
17	17	0	12.714073	-2.706438	-0.759293
18	5	0	2.504554	-0.688057	0.916822
19	6	0	2.397004	-1.261634	2.462724
20	1	0	1.915597	-2.255057	2.446559
21	1	0	3.436742	-1.430684	2.807894
22	6	0	1.691736	-0.367209	3.430647
23	6	0	0.527307	-0.623914	4.048913
24	1	0	2.161220	0.608910	3.617717
25	1	0	0.054851	0.107747	4.712934
26	1	0	0.002214	-1.575749	3.911022
27	6	0	3.182601	-1.824470	-0.068176
28	1	0	2.632784	-2.778805	0.019189
29	6	0	3.314343	-1.433941	-1.504452
30	6	0	2.717705	-2.028633	-2.551345
31	1	0	3.945335	-0.557067	-1.700446
32	1	0	2.845857	-1.655563	-3.573288
33	1	0	2.083472	-2.913476	-2.424739
34	1	0	4.194354	-2.006718	0.345148
35	8	0	-0.339945	2.967314	0.051841
36	6	0	0.670463	2.017927	-0.190312
37	6	0	0.178134	0.590118	-0.013377
38	6	0	-1.090174	0.089497	-0.232086
39	7	0	0.933158	-0.488465	0.398624
40	1	0	-2.024196	0.561691	-0.518303
41	7	0	0.216077	-1.582246	0.440263
42	7	0	-1.006583	-1.235512	0.060485
43	6	0	-2.047946	-2.208401	0.003897
44	6	0	-2.165538	-3.064990	-1.103677
45	6	0	-2.993360	-2.310151	1.038457
46	6	0	-3.181226	-4.019345	-1.175716
47	6	0	-4.024355	-3.248962	0.985314
48	6	0	-4.097045	-4.095077	-0.123570
49	1	0	-3.256504	-4.679324	-2.038824
50	1	0	-4.755253	-3.309770	1.790268
51	17	0	-5.374716	-5.275297	-0.200947
52	5	0	-1.492432	3.522082	-0.577500
53	6	0	-1.539235	3.470586	-2.315791
54	1	0	-0.717741	4.132194	-2.624881

55	1	0	-1.343311	2.431543	-2.613579
56	6	0	-2.844345	3.937206	-2.727094
57	6	0	-3.949823	3.127506	-2.805892
58	1	0	-3.001160	5.021679	-2.790689
59	1	0	-4.935183	3.539744	-3.042156
60	1	0	-3.840458	2.042678	-2.891923
61	6	0	-1.772906	5.015879	0.036383
62	1	0	-1.745580	4.916336	1.136596
63	6	0	-0.794963	6.060157	-0.403481
64	6	0	0.199871	6.579907	0.332026
65	1	0	-0.896954	6.410101	-1.441178
66	1	0	0.892396	7.320079	-0.081711
67	1	0	0.364543	6.277182	1.372406
68	1	0	-2.790803	5.358462	-0.233601
69	6	0	4.202233	2.545624	1.730856
70	1	0	4.109920	3.043159	0.750215
71	1	0	3.279973	2.749827	2.296955
72	1	0	4.336699	0.511185	2.440551
73	6	0	5.395149	3.083504	2.468259
74	6	0	5.366530	3.601966	3.703287
75	1	0	6.355299	3.036833	1.938003
76	1	0	6.272289	3.981639	4.186927
77	1	0	4.434157	3.675142	4.275119
78	17	0	7.357246	-2.703252	-1.505175
79	17	0	9.739538	1.378837	1.165952
80	17	0	-1.043937	-2.923040	-2.418974
81	17	0	-2.891353	-1.246523	2.404809
82	8	0	-2.715443	2.602186	-0.281317
83	6	0	-3.936119	2.910623	-0.504198
84	1	0	-4.280313	3.943042	-0.369871
85	6	0	-4.938263	1.853348	-0.404164
86	6	0	-6.295901	1.949027	-0.141789
87	7	0	-4.628237	0.527320	-0.555804
88	1	0	-6.958146	2.788207	0.051885
89	7	0	-5.687778	-0.189512	-0.403654
90	7	0	-6.721340	0.661068	-0.145292
91	6	0	-8.030517	0.155680	0.077052
92	6	0	-8.369033	-0.434428	1.307838
93	6	0	-9.007785	0.214199	-0.932127
94	6	0	-9.643976	-0.954249	1.535455
95	6	0	-10.291143	-0.294309	-0.728902
96	6	0	-10.588121	-0.874571	0.507726
97	1	0	-9.891431	-1.407223	2.494851
98	1	0	-11.035998	-0.242102	-1.522162
99	17	0	-8.613300	0.928070	-2.465753
100	17	0	-7.183695	-0.505288	2.573771
101	17	0	-12.182388	-1.516471	0.776527
102	1	0	1.422308	2.172520	0.593859
103	6	0	1.412601	2.180386	-1.540459
104	1	0	2.199168	1.412987	-1.575350
105	1	0	0.713609	1.966632	-2.366562
106	6	0	2.021018	3.543963	-1.715638
107	6	0	3.309745	3.774826	-1.999656
108	1	0	1.345670	4.397850	-1.588410
109	1	0	3.688336	4.795209	-2.120578
110	1	0	4.035272	2.961706	-2.107716

9a(*S*)

Sum of electronic and zero-point Energies= -6646.384439
Sum of electronic and thermal Energies= -6646.314634
Sum of electronic and thermal Enthalpies= -6646.313690
Sum of electronic and thermal Free Energies= -6646.506884

Standard orientation:

Center Number	Atomic Number	Atomic Type	Coordinates (Angstroms) X	Y	Z
1	8	0	-2.906847	0.722774	-0.247696
2	6	0	-4.006949	1.120880	-1.025597
3	6	0	-5.331073	0.694084	-0.431340
4	6	0	-6.341266	-0.072414	-0.978842
5	7	0	-5.741818	1.093711	0.812318
6	1	0	-6.453509	-0.581669	-1.931656
7	7	0	-6.922019	0.626488	1.066132
8	7	0	-7.310309	-0.092190	-0.019613
9	6	0	-8.595200	-0.689865	-0.064149
10	6	0	-8.778606	-2.053010	0.228759
11	6	0	-9.728346	0.079104	-0.387145
12	6	0	-10.044810	-2.640675	0.202090
13	6	0	-11.006592	-0.480640	-0.417933
14	6	0	-11.142668	-1.839166	-0.120678
15	1	0	-10.167079	-3.698059	0.433350
16	1	0	-11.872165	0.130732	-0.670039
17	17	0	-12.730097	-2.555422	-0.154462
18	5	0	-2.450100	-0.661759	-0.120843
19	6	0	-2.574001	-1.570649	-1.487363
20	1	0	-2.277870	-2.611906	-1.272561
21	1	0	-3.657316	-1.605702	-1.720271
22	6	0	-1.827230	-1.065998	-2.681890
23	6	0	-0.833014	-1.698416	-3.325679
24	1	0	-2.103273	-0.063589	-3.039100
25	1	0	-0.314723	-1.239299	-4.174089
26	1	0	-0.496423	-2.697334	-3.024687
27	6	0	-3.078012	-1.457925	1.181305
28	1	0	-2.552112	-2.419332	1.325400
29	6	0	-3.088342	-0.669497	2.449715
30	6	0	-2.395706	-0.939889	3.569723
31	1	0	-3.711498	0.234213	2.429908
32	1	0	-2.447480	-0.287027	4.447790
33	1	0	-1.758769	-1.828419	3.654476
34	1	0	-4.122435	-1.702470	0.906602
35	8	0	0.071085	2.814320	-0.138279
36	6	0	-0.465910	1.935680	0.866486
37	6	0	-0.008486	0.516918	0.633122
38	6	0	1.252644	-0.026134	0.793001
39	7	0	-0.814604	-0.516983	0.210729
40	1	0	2.215601	0.398464	1.081280
41	7	0	-0.137660	-1.627998	0.103965
42	7	0	1.111042	-1.334044	0.452699
43	6	0	2.122488	-2.336616	0.391762
44	6	0	2.297385	-3.247961	1.445820
45	6	0	2.969405	-2.421732	-0.725924
46	6	0	3.284596	-4.233035	1.390431
47	6	0	3.968089	-3.392980	-0.802495
48	6	0	4.105789	-4.288282	0.261384
49	1	0	3.408559	-4.936095	2.212997
50	1	0	4.619653	-3.446214	-1.673327
51	17	0	5.341578	-5.511733	0.178552
52	5	0	1.347406	3.256890	-0.333369
53	6	0	2.399102	5.692630	2.897063
54	1	0	1.870729	6.650366	2.927628

55	1	0	2.234531	5.010883	3.738276
56	6	0	3.213390	5.375426	1.883177
57	6	0	3.997371	4.098991	1.771768
58	1	0	3.350198	6.095362	1.065013
59	1	0	5.076769	4.333433	1.755253
60	1	0	3.816916	3.446953	2.641150
61	6	0	1.605797	4.246407	-1.557790
62	1	0	2.444857	3.865042	-2.168856
63	6	0	0.410466	4.509742	-2.426420
64	6	0	0.313009	4.224744	-3.731983
65	1	0	-0.450551	4.970516	-1.925956
66	1	0	-0.599117	4.441204	-4.297150
67	1	0	1.136500	3.757790	-4.285117
68	1	0	1.959367	5.206494	-1.129241
69	6	0	-3.930787	2.659551	-1.163331
70	1	0	-4.002154	3.075459	-0.142699
71	1	0	-2.932427	2.903349	-1.557711
72	1	0	-3.957419	0.685386	-2.040305
73	6	0	-5.000791	3.254246	-2.033406
74	6	0	-4.783131	3.875062	-3.200416
75	1	0	-6.032333	3.160792	-1.669056
76	1	0	-5.605476	4.292039	-3.790633
77	1	0	-3.772115	3.996145	-3.606632
78	17	0	-7.405155	-3.034665	0.636071
79	17	0	-9.534815	1.762904	-0.767750
80	17	0	1.279791	-3.144963	2.846567
81	17	0	2.769597	-1.302429	-2.036446
82	8	0	2.346376	2.840804	0.517989
83	6	0	3.694949	3.299772	0.484982
84	1	0	3.859001	3.955893	-0.384752
85	6	0	4.621075	2.117353	0.359171
86	6	0	5.860314	2.031143	-0.245482
87	7	0	4.368025	0.917819	0.960430
88	1	0	6.458732	2.730994	-0.821670
89	7	0	5.355622	0.100695	0.763069
90	7	0	6.281029	0.765197	0.033114
91	6	0	7.517646	0.148557	-0.297296
92	6	0	7.731968	-0.423889	-1.563167
93	6	0	8.556289	0.086107	0.649613
94	6	0	8.940719	-1.041947	-1.887289
95	6	0	9.773404	-0.529884	0.353881
96	6	0	9.945335	-1.085616	-0.916796
97	1	0	9.089612	-1.481766	-2.872770
98	1	0	10.567953	-0.571283	1.098087
99	17	0	8.325326	0.800144	2.214908
100	17	0	6.466400	-0.366193	-2.751430
101	17	0	11.458199	-1.854040	-1.303023
102	1	0	-1.545645	1.941665	0.691024
103	6	0	-0.194228	2.413541	2.306735
104	1	0	-0.642918	1.671476	2.985213
105	1	0	0.893272	2.421568	2.486928
106	6	0	-0.774708	3.773569	2.580268
107	6	0	-1.705300	4.033765	3.507381
108	1	0	-0.395117	4.596773	1.963134
109	1	0	-2.090409	5.046632	3.661608
110	1	0	-2.119003	3.245288	4.146057

TS1a(*R*)

Sum of electronic and zero-point Energies= -6646.311069
Sum of electronic and thermal Energies= -6646.240992
Sum of electronic and thermal Enthalpies= -6646.240048
Sum of electronic and thermal Free Energies= -6646.433158

Standard orientation:

Center Number	Atomic Number	Atomic Type	Coordinates (Angstroms) X	Y	Z
1	8	0	-1.989219	-0.097304	1.723275
2	6	0	-2.435384	-1.265427	2.376034
3	6	0	-2.947859	-2.326646	1.425315
4	6	0	-4.191752	-2.924311	1.357117
5	7	0	-2.149675	-2.928070	0.491272
6	1	0	-5.118918	-2.773911	1.902659
7	7	0	-2.811878	-3.839673	-0.147223
8	7	0	-4.067736	-3.855139	0.368735
9	6	0	-5.030020	-4.781926	-0.107686
10	6	0	-5.835033	-4.483647	-1.221752
11	6	0	-5.185734	-6.035875	0.510062
12	6	0	-6.772630	-5.397050	-1.708001
13	6	0	-6.115063	-6.968105	0.046108
14	6	0	-6.897411	-6.628983	-1.060979
15	1	0	-7.388268	-5.149491	-2.571992
16	1	0	-6.220970	-7.934876	0.536576
17	17	0	-8.064624	-7.778665	-1.652829
18	5	0	-2.930655	0.964669	1.366728
19	6	0	-3.767559	1.584047	2.653068
20	1	0	-4.490078	2.340018	2.302119
21	1	0	-4.374021	0.741150	3.041826
22	6	0	-2.943695	2.141565	3.767172
23	6	0	-2.914572	3.421809	4.173624
24	1	0	-2.282049	1.428942	4.277055
25	1	0	-2.254036	3.754192	4.981702
26	1	0	-3.554979	4.184813	3.716743
27	6	0	-3.988976	0.619290	0.150312
28	1	0	-4.642821	1.492567	-0.026008
29	6	0	-3.417337	0.145947	-1.146607
30	6	0	-3.605121	0.716273	-2.349055
31	1	0	-2.780953	-0.745973	-1.092579
32	1	0	-3.143163	0.312703	-3.256335
33	1	0	-4.239827	1.601666	-2.472031
34	1	0	-4.644550	-0.176637	0.556958
35	8	0	0.419452	0.456667	-0.553778
36	6	0	0.543441	1.542368	0.347793
37	6	0	-0.676018	2.445642	0.377995
38	6	0	-0.619695	3.769425	-0.009616
39	7	0	-1.980979	2.233444	0.786134
40	1	0	0.198010	4.366872	-0.399881
41	7	0	-2.687148	3.327425	0.655532
42	7	0	-1.871295	4.257861	0.177826
43	6	0	-2.354408	5.575557	-0.077345
44	6	0	-3.037797	5.866095	-1.270667
45	6	0	-2.146593	6.608694	0.852490
46	6	0	-3.524384	7.148188	-1.529779
47	6	0	-2.619421	7.899996	0.614066
48	6	0	-3.307340	8.147518	-0.577154
49	1	0	-4.055687	7.358855	-2.457146
50	1	0	-2.454162	8.689875	1.345754
51	17	0	-3.905999	9.750830	-0.887724
52	5	0	0.690200	0.485853	-1.914746
53	6	0	0.650905	1.808672	-2.834082
54	1	0	0.829973	1.488173	-3.880569

55	1	0	-0.424874	2.084345	-2.847445
56	6	0	1.440984	3.081829	-2.649390
57	6	0	2.593062	3.283184	-1.990925
58	1	0	1.020687	3.948205	-3.181879
59	1	0	3.070836	4.268828	-1.993722
60	1	0	3.106577	2.486665	-1.445845
61	6	0	0.357128	-0.897277	-2.668750
62	1	0	0.858260	-0.944145	-3.651946
63	6	0	0.588037	-2.156860	-1.893383
64	6	0	1.382231	-3.174355	-2.263503
65	1	0	0.057059	-2.229597	-0.936644
66	1	0	1.505921	-4.059673	-1.630920
67	1	0	1.926037	-3.173961	-3.216231
68	1	0	-0.727029	-0.812934	-2.894048
69	1	0	1.350611	2.207944	0.006370
70	6	0	0.913908	1.011183	1.742320
71	1	0	0.082745	0.355953	2.044298
72	1	0	0.950599	1.861108	2.442731
73	6	0	2.207076	0.251640	1.771630
74	6	0	3.241863	0.528952	2.577157
75	1	0	2.270084	-0.603509	1.089456
76	1	0	4.145267	-0.089785	2.583148
77	1	0	3.219776	1.372381	3.277199
78	1	0	-3.267359	-1.034009	3.066139
79	6	0	-1.283628	-1.878376	3.211408
80	1	0	-1.606514	-2.871691	3.562343
81	1	0	-0.436683	-2.038389	2.521046
82	6	0	-0.851677	-1.050631	4.386666
83	6	0	-0.927975	-1.442287	5.665913
84	1	0	-0.445113	-0.058505	4.162205
85	1	0	-0.593936	-0.795875	6.483746
86	1	0	-1.321776	-2.426561	5.945269
87	17	0	-1.283258	6.281851	2.321260
88	17	0	-3.265628	4.617337	-2.451787
89	17	0	-4.198205	-6.436135	1.882096
90	17	0	-5.662801	-2.946924	-2.008873
91	6	0	3.547969	-0.475043	-2.517785
92	6	0	4.911669	-0.878397	-2.201666
93	6	0	5.664479	-0.665832	-1.053406
94	7	0	5.676546	-1.594223	-3.087131
95	1	0	5.452821	-0.158551	-0.116015
96	7	0	6.829060	-1.833002	-2.573418
97	7	0	6.840983	-1.270088	-1.319646
98	6	0	7.994031	-1.384032	-0.495852
99	6	0	8.204051	-2.537613	0.279986
100	6	0	8.959884	-0.363359	-0.464299
101	6	0	9.343849	-2.677075	1.073188
102	6	0	10.107447	-0.477801	0.321373
103	6	0	10.280048	-1.639342	1.079692
104	1	0	9.494765	-3.575864	1.669670
105	1	0	10.848626	0.320287	0.335916
106	17	0	7.021546	-3.806255	0.254328
107	17	0	8.721914	1.065828	-1.420986
108	17	0	11.706128	-1.798538	2.063695
109	1	0	3.180533	-0.803303	-3.510565
110	8	0	2.853716	0.188758	-1.756056

8a(*R*)

Sum of electronic and zero-point Energies= -6646.311301
Sum of electronic and thermal Energies= -6646.241367
Sum of electronic and thermal Enthalpies= -6646.240423
Sum of electronic and thermal Free Energies= -6646.431237

Standard orientation:

Center Number	Atomic Number	Atomic Type	Coordinates (Angstroms) X	Y	Z
1	8	0	-2.008304	0.069394	1.876756
2	6	0	-2.579573	-1.051518	2.511064
3	6	0	-3.091768	-2.095843	1.540893
4	6	0	-4.362586	-2.616390	1.388578
5	7	0	-2.265734	-2.766033	0.680420
6	1	0	-5.315993	-2.398097	1.861081
7	7	0	-2.934974	-3.649131	0.009948
8	7	0	-4.224821	-3.575135	0.429039
9	6	0	-5.202608	-4.457197	-0.098191
10	6	0	-5.987093	-4.094855	-1.207718
11	6	0	-5.392580	-5.732841	0.463315
12	6	0	-6.935409	-4.967457	-1.745209
13	6	0	-6.331456	-6.626691	-0.054470
14	6	0	-7.090828	-6.224863	-1.156560
15	1	0	-7.533864	-4.670075	-2.605315
16	1	0	-6.463468	-7.611149	0.392667
17	17	0	-8.268601	-7.325601	-1.817307
18	5	0	-2.833734	1.212633	1.483897
19	6	0	-3.577270	1.966616	2.757612
20	1	0	-4.245567	2.762268	2.386926
21	1	0	-4.237355	1.196189	3.205311
22	6	0	-2.678356	2.507526	3.820267
23	6	0	-2.529476	3.799076	4.159746
24	1	0	-2.064667	1.767200	4.349752
25	1	0	-1.819740	4.112291	4.933051
26	1	0	-3.114840	4.591757	3.679863
27	6	0	-3.947874	0.930515	0.301386
28	1	0	-4.540311	1.847079	0.127496
29	6	0	-3.445070	0.401111	-1.002541
30	6	0	-3.634551	0.961205	-2.209360
31	1	0	-2.857011	-0.523152	-0.951854
32	1	0	-3.219128	0.518183	-3.120735
33	1	0	-4.221323	1.879203	-2.331078
34	1	0	-4.650223	0.191995	0.737777
35	8	0	0.363433	0.265559	-0.479259
36	6	0	0.650500	1.335089	0.386136
37	6	0	-0.448424	2.388084	0.415919
38	6	0	-0.249910	3.682546	-0.020788
39	7	0	-1.763985	2.339907	0.841969
40	1	0	0.622632	4.169948	-0.444131
41	7	0	-2.346637	3.500433	0.676043
42	7	0	-1.437932	4.314714	0.156710
43	6	0	-1.778028	5.664476	-0.152439
44	6	0	-2.464861	5.972615	-1.339724
45	6	0	-1.428932	6.711465	0.717764
46	6	0	-2.815051	7.287291	-1.651113
47	6	0	-1.764339	8.034599	0.426452
48	6	0	-2.458884	8.300367	-0.756838
49	1	0	-3.350451	7.511153	-2.573115
50	1	0	-1.490704	8.835488	1.112009
51	17	0	-2.888729	9.944123	-1.131113
52	5	0	0.809593	0.196144	-1.841005
53	6	0	0.736345	1.511186	-2.810120
54	1	0	0.805930	1.157420	-3.860352

55	1	0	-0.311196	1.868212	-2.746410
56	6	0	1.634528	2.721937	-2.737698
57	6	0	2.807152	2.887743	-2.104393
58	1	0	1.280028	3.577068	-3.333971
59	1	0	3.360007	3.829467	-2.189552
60	1	0	3.265883	2.107091	-1.491050
61	6	0	0.161764	-1.130104	-2.556757
62	1	0	0.454491	-1.196035	-3.620637
63	6	0	0.460186	-2.410239	-1.851682
64	6	0	1.191715	-3.430688	-2.336694
65	1	0	0.071069	-2.485256	-0.829059
66	1	0	1.394904	-4.324080	-1.736863
67	1	0	1.584873	-3.430265	-3.360810
68	1	0	-0.927810	-0.942212	-2.540049
69	1	0	1.527515	1.895108	0.019980
70	6	0	0.985478	0.814917	1.797878
71	1	0	0.081258	0.293622	2.147623
72	1	0	1.161896	1.677875	2.460872
73	6	0	2.156217	-0.120579	1.833041
74	6	0	3.252874	0.041732	2.587615
75	1	0	2.065662	-1.013624	1.204247
76	1	0	4.052466	-0.706376	2.605149
77	1	0	3.383129	0.914736	3.237928
78	1	0	-3.439538	-0.751644	3.138348
79	6	0	-1.532806	-1.729994	3.430553
80	1	0	-1.969455	-2.667764	3.810439
81	1	0	-0.677689	-2.007151	2.789177
82	6	0	-1.065462	-0.890251	4.582857
83	6	0	-1.250647	-1.193361	5.874925
84	1	0	-0.530986	0.031185	4.327076
85	1	0	-0.881942	-0.543093	6.674618
86	1	0	-1.773942	-2.105905	6.183810
87	17	0	-0.562594	6.361141	2.179794
88	17	0	-2.872436	4.701712	-2.446002
89	17	0	-4.440063	-6.206406	1.836343
90	17	0	-5.773243	-2.530089	-1.926235
91	6	0	3.135257	-0.843910	-2.457495
92	6	0	4.490687	-1.235708	-2.164180
93	6	0	5.267782	-1.025773	-1.028196
94	7	0	5.240720	-1.945867	-3.070202
95	1	0	5.072859	-0.524790	-0.083396
96	7	0	6.400873	-2.182178	-2.581228
97	7	0	6.437435	-1.624066	-1.321492
98	6	0	7.606548	-1.743125	-0.519994
99	6	0	7.825412	-2.899431	0.249259
100	6	0	8.575573	-0.725195	-0.505052
101	6	0	8.978803	-3.044024	1.021249
102	6	0	9.736651	-0.845716	0.259447
103	6	0	9.918477	-2.009281	1.012740
104	1	0	9.137745	-3.944380	1.613309
105	1	0	10.481035	-0.050455	0.262154
106	17	0	6.637398	-4.162920	0.241996
107	17	0	8.323728	0.706971	-1.453267
108	17	0	11.360345	-2.175089	1.971224
109	1	0	2.732356	-1.148834	-3.435643
110	8	0	2.463649	-0.160987	-1.665095

TS2a(*R*)

Sum of electronic and zero-point Energies= -6646.311644
Sum of electronic and thermal Energies= -6646.243737
Sum of electronic and thermal Enthalpies= -6646.242793
Sum of electronic and thermal Free Energies= -6646.427549

Standard orientation:

Center Number	Atomic Number	Atomic Type	Coordinates (Angstroms) X	Y	Z
1	8	0	-1.836884	0.130593	1.903350
2	6	0	-2.430346	-0.992132	2.511278
3	6	0	-3.049182	-1.965724	1.527763
4	6	0	-4.346482	-2.436608	1.459631
5	7	0	-2.321232	-2.624685	0.573929
6	1	0	-5.249250	-2.208031	2.018843
7	7	0	-3.076269	-3.453321	-0.075069
8	7	0	-4.323848	-3.355772	0.453021
9	6	0	-5.372941	-4.184433	-0.021009
10	6	0	-6.266860	-3.734502	-1.009154
11	6	0	-5.531154	-5.491409	0.474876
12	6	0	-7.291958	-4.551353	-1.490360
13	6	0	-6.544631	-6.331189	0.009886
14	6	0	-7.412784	-5.842259	-0.969854
15	1	0	-7.975785	-4.186030	-2.255462
16	1	0	-6.651227	-7.340834	0.404672
17	17	0	-8.686052	-6.874274	-1.560293
18	5	0	-2.617384	1.338243	1.630985
19	6	0	-3.166036	2.098376	2.997253
20	1	0	-3.837768	2.930100	2.723409
21	1	0	-3.799714	1.346915	3.510661
22	6	0	-2.121680	2.580445	3.948953
23	6	0	-1.876916	3.858253	4.284538
24	1	0	-1.480693	1.805679	4.389023
25	1	0	-1.065398	4.128190	4.968885
26	1	0	-2.482589	4.682495	3.890699
27	6	0	-3.869617	1.165561	0.572770
28	1	0	-4.410420	2.124436	0.478747
29	6	0	-3.558018	0.628345	-0.786875
30	6	0	-3.850776	1.216817	-1.958862
31	1	0	-3.030807	-0.332968	-0.813878
32	1	0	-3.573278	0.759901	-2.914832
33	1	0	-4.385211	2.172827	-2.005231
34	1	0	-4.571758	0.469957	1.076192
35	8	0	0.363512	0.201166	-0.418382
36	6	0	0.781143	1.287556	0.358333
37	6	0	-0.252137	2.403539	0.419358
38	6	0	-0.014993	3.686638	-0.029898
39	7	0	-1.543800	2.416852	0.911253
40	1	0	0.855052	4.141433	-0.493512
41	7	0	-2.078889	3.604481	0.776666
42	7	0	-1.159990	4.375693	0.211281
43	6	0	-1.443496	5.743537	-0.076188
44	6	0	-2.187769	6.092863	-1.216758
45	6	0	-0.971843	6.767478	0.763507
46	6	0	-2.477643	7.426712	-1.508567
47	6	0	-1.246164	8.108461	0.490722
48	6	0	-2.001848	8.415891	-0.643917
49	1	0	-3.057088	7.683525	-2.394551
50	1	0	-0.878439	8.891960	1.152279
51	17	0	-2.354238	10.082679	-0.994883
52	5	0	0.783327	0.009644	-1.786342
53	6	0	0.538885	1.278998	-2.804154
54	1	0	-0.553617	1.416944	-2.886747

55	1	0	0.940260	2.181519	-2.305432
56	6	0	1.159081	1.174765	-4.159761
57	6	0	0.525347	0.906472	-5.314365
58	1	0	2.249250	1.314046	-4.201365
59	1	0	1.069064	0.821073	-6.260871
60	1	0	-0.561522	0.769000	-5.355434
61	6	0	0.058362	-1.408082	-2.361365
62	1	0	0.040343	-1.381366	-3.463092
63	6	0	0.808949	-2.540596	-1.834292
64	6	0	1.845818	-3.158410	-2.474648
65	1	0	0.659232	-2.759650	-0.769793
66	1	0	2.450977	-3.911042	-1.959907
67	1	0	1.955940	-3.116450	-3.561842
68	1	0	-0.962060	-1.352144	-1.955171
69	1	0	1.666929	1.769835	-0.097858
70	6	0	1.181705	0.827362	1.776507
71	1	0	0.268618	0.392088	2.212303
72	1	0	1.461819	1.710345	2.374073
73	6	0	2.279668	-0.192255	1.797451
74	6	0	3.416250	-0.094961	2.502500
75	1	0	2.094781	-1.094864	1.204278
76	1	0	4.152239	-0.905628	2.512955
77	1	0	3.643198	0.784347	3.116700
78	1	0	-3.240413	-0.684113	3.199088
79	6	0	-1.373339	-1.765284	3.341125
80	1	0	-1.827292	-2.712024	3.675633
81	1	0	-0.553803	-2.024221	2.647892
82	6	0	-0.835796	-1.021817	4.528616
83	6	0	-0.998900	-1.398600	5.804128
84	1	0	-0.268586	-0.108756	4.316695
85	1	0	-0.581104	-0.816218	6.631492
86	1	0	-1.552485	-2.306557	6.070883
87	17	0	-0.025129	6.367798	2.162180
88	17	0	-2.737844	4.850194	-2.291758
89	17	0	-4.444896	-6.073338	1.698959
90	17	0	-6.091686	-2.130184	-1.646831
91	6	0	2.946772	-1.057288	-2.504079
92	6	0	4.307962	-1.454149	-2.188548
93	6	0	5.063090	-1.207731	-1.050492
94	7	0	5.075834	-2.179629	-3.063470
95	1	0	4.853836	-0.672541	-0.128509
96	7	0	6.233645	-2.398069	-2.547229
97	7	0	6.247430	-1.808388	-1.311041
98	6	0	7.402140	-1.892907	-0.488258
99	6	0	7.446245	-2.777058	0.604749
100	6	0	8.535733	-1.107622	-0.764102
101	6	0	8.583943	-2.883797	1.405604
102	6	0	9.687929	-1.199695	0.017991
103	6	0	9.691560	-2.090031	1.095140
104	1	0	8.603841	-3.574317	2.248014
105	1	0	10.559423	-0.586692	-0.208180
106	17	0	6.060974	-3.760482	0.968430
107	17	0	8.497558	0.009823	-2.091140
108	17	0	11.119091	-2.212153	2.082214
109	1	0	2.630938	-1.140802	-3.549828
110	8	0	2.329477	-0.283146	-1.703781

9a(*R*)

Sum of electronic and zero-point Energies= -6646.370230
Sum of electronic and thermal Energies= -6646.300628
Sum of electronic and thermal Enthalpies= -6646.299684
Sum of electronic and thermal Free Energies= -6646.492322

Standard orientation:

Center Number	Atomic Number	Atomic Type	Coordinates (Angstroms) X	Y	Z
1	8	0	-1.183081	0.259780	-1.959662
2	6	0	-0.034938	0.702989	-2.647584
3	6	0	1.074674	1.174461	-1.728819
4	6	0	1.667429	2.418142	-1.616426
5	7	0	1.737819	0.327888	-0.882475
6	1	0	1.479923	3.375355	-2.094445
7	7	0	2.680266	0.961435	-0.259195
8	7	0	2.656042	2.243526	-0.693581
9	6	0	3.602524	3.178299	-0.198779
10	6	0	3.338948	3.930493	0.959954
11	6	0	4.847066	3.342670	-0.832198
12	6	0	4.275977	4.830435	1.471567
13	6	0	5.802367	4.234906	-0.343407
14	6	0	5.497463	4.967245	0.807204
15	1	0	4.055931	5.404846	2.370732
16	1	0	6.761140	4.350262	-0.847368
17	17	0	6.679736	6.080983	1.435233
18	5	0	-2.263060	1.188577	-1.615919
19	6	0	-2.980057	1.904818	-2.924620
20	1	0	-3.722965	2.646532	-2.586256
21	1	0	-2.172412	2.484863	-3.415345
22	6	0	-3.595237	0.988228	-3.931254
23	6	0	-4.901003	0.895110	-4.234434
24	1	0	-2.902733	0.311579	-4.448370
25	1	0	-5.270405	0.169561	-4.967095
26	1	0	-5.649561	1.543668	-3.764686
27	6	0	-1.896129	2.342514	-0.498338
28	1	0	-2.756084	3.026134	-0.376821
29	6	0	-1.434471	1.873827	0.844339
30	6	0	-1.983403	2.210875	2.023567
31	1	0	-0.587132	1.177681	0.850955
32	1	0	-1.601136	1.813365	2.969651
33	1	0	-2.830082	2.904916	2.086665
34	1	0	-1.090981	2.946406	-0.963513
35	8	0	-1.421206	-1.646615	0.668000
36	6	0	-2.494394	-2.101613	-0.133549
37	6	0	-3.526642	-1.007233	-0.299132
38	6	0	-4.825216	-1.122010	0.151493
39	7	0	-3.447869	0.233503	-0.902179
40	1	0	-5.338497	-1.919574	0.680375
41	7	0	-4.598637	0.850193	-0.836359
42	7	0	-5.434849	0.038163	-0.203254
43	6	0	-6.793600	0.422512	-0.001446
44	6	0	-7.141451	1.309739	1.031787
45	6	0	-7.808591	-0.082937	-0.832571
46	6	0	-8.467438	1.696830	1.232268
47	6	0	-9.141759	0.285389	-0.647640
48	6	0	-9.449236	1.175055	0.385635
49	1	0	-8.724448	2.386873	2.034857
50	1	0	-9.918880	-0.111219	-1.299780
51	17	0	-11.106293	1.644168	0.627130
52	5	0	-0.928492	-2.282275	1.788542
53	6	0	-1.583136	-3.645631	2.313471
54	1	0	-2.575901	-3.368839	2.720961

55	1	0	-1.793677	-4.258763	1.415779
56	6	0	-0.866554	-4.520583	3.302076
57	6	0	-1.297221	-4.828923	4.533819
58	1	0	0.075548	-4.964776	2.954155
59	1	0	-0.723134	-5.490929	5.189296
60	1	0	-2.231797	-4.422069	4.937054
61	6	0	-0.202227	-0.571728	6.484195
62	1	0	0.658544	-0.820560	7.115394
63	6	0	-0.102946	-0.514784	5.150098
64	6	0	1.153688	-0.774654	4.368763
65	1	0	-0.990954	-0.259557	4.558660
66	1	0	1.436366	0.125527	3.793849
67	1	0	1.986872	-1.015152	5.047567
68	1	0	-1.147122	-0.366470	6.997280
69	1	0	-3.039986	-2.904095	0.388663
70	6	0	-1.996326	-2.668521	-1.477598
71	1	0	-1.501449	-1.834632	-1.997194
72	1	0	-2.878088	-2.971140	-2.065131
73	6	0	-1.049423	-3.824004	-1.327619
74	6	0	-1.270333	-5.063818	-1.785568
75	1	0	-0.097443	-3.607508	-0.826986
76	1	0	-0.525906	-5.857530	-1.668055
77	1	0	-2.197289	-5.330099	-2.306528
78	1	0	-0.281151	1.552326	-3.310809
79	6	0	0.531123	-0.441228	-3.525559
80	1	0	1.471923	-0.089428	-3.979019
81	1	0	0.791493	-1.270026	-2.843631
82	6	0	-0.395542	-0.932059	-4.598555
83	6	0	-0.151238	-0.859555	-5.913974
84	1	0	-1.332706	-1.386218	-4.258093
85	1	0	-0.864270	-1.242442	-6.650969
86	1	0	0.773001	-0.418619	-6.305411
87	17	0	-7.404098	-1.184005	-2.112109
88	17	0	-5.908956	1.922688	2.084752
89	17	0	5.207466	2.411550	-2.254657
90	17	0	1.820098	3.732801	1.774748
91	6	0	1.016839	-1.933465	3.361088
92	6	0	2.328095	-2.233877	2.686166
93	6	0	2.726406	-1.932686	1.398853
94	7	0	3.368100	-2.844369	3.327391
95	1	0	2.234374	-1.442177	0.560966
96	7	0	4.382659	-2.943259	2.524737
97	7	0	4.008506	-2.390681	1.342378
98	6	0	4.909241	-2.331563	0.248948
99	6	0	5.915671	-1.350517	0.197311
100	6	0	4.816383	-3.245741	-0.815301
101	6	0	6.806897	-1.278016	-0.874800
102	6	0	5.691246	-3.194793	-1.901646
103	6	0	6.677356	-2.205163	-1.911328
104	1	0	7.577350	-0.508602	-0.900048
105	1	0	5.603592	-3.911985	-2.716680
106	17	0	6.053503	-0.197539	1.486754
107	17	0	3.588247	-4.476237	-0.781033
108	17	0	7.779860	-2.123944	-3.258720
109	1	0	0.676177	-2.830569	3.900637
110	8	0	0.085485	-1.560378	2.353534

3b(*R*)

Sum of electronic and zero-point Energies= -986.859871
Sum of electronic and thermal Energies= -986.832392
Sum of electronic and thermal Enthalpies= -986.831447
Sum of electronic and thermal Free Energies= -986.923976

Standard orientation:

Center Number	Atomic Number	Atomic Type	Coordinates (Angstroms) X	Y	Z
1	8	0	2.837885	-0.010680	-0.618154
2	6	0	2.114803	-1.150535	-0.147871
3	5	0	3.854424	0.633289	0.027932
4	6	0	4.531156	1.852001	-0.738708
5	1	0	4.686331	2.679665	-0.022488
6	1	0	5.553146	1.492687	-0.988967
7	6	0	3.849037	2.335768	-1.982818
8	6	0	3.334621	3.559102	-2.171133
9	1	0	3.757039	1.601936	-2.794258
10	1	0	2.833676	3.831691	-3.105504
11	1	0	3.390944	4.334077	-1.397650
12	6	0	4.376902	0.230128	1.485143
13	1	0	5.431482	0.536803	1.585274
14	6	0	3.546012	0.927774	2.531981
15	6	0	3.933583	1.980806	3.266163
16	1	0	2.524170	0.549139	2.668073
17	1	0	3.258192	2.450973	3.987566
18	1	0	4.938909	2.408206	3.177346
19	1	0	4.333761	-0.863114	1.643862
20	6	0	2.428556	-2.323168	-1.107571
21	1	0	2.134116	-2.006796	-2.123754
22	1	0	3.521628	-2.461840	-1.105246
23	1	0	2.451193	-1.431447	0.862923
24	6	0	1.743617	-3.611880	-0.747665
25	6	0	2.370111	-4.708313	-0.302372
26	1	0	0.653211	-3.636707	-0.867240
27	1	0	1.819128	-5.622181	-0.059971
28	1	0	3.457849	-4.737532	-0.171432
29	6	0	0.643256	-0.802114	-0.086689
30	6	0	0.029644	0.064305	-1.004394
31	6	0	-0.206857	-1.339385	0.889540
32	7	0	-1.273975	0.345089	-0.970049
33	1	0	0.619494	0.557677	-1.783460
34	7	0	-1.513020	-1.073218	0.941808
35	1	0	0.187020	-2.009475	1.663128
36	6	0	-1.999159	-0.243259	0.000247
37	6	0	-3.408160	0.043476	0.037827
38	6	0	-4.603783	0.284678	0.070656
39	6	0	-6.047360	0.573282	0.108094
40	6	0	-6.549980	0.463457	1.565720
41	1	0	-6.379209	-0.547377	1.967235
42	1	0	-6.033713	1.185788	2.216864
43	1	0	-7.631116	0.674212	1.602434
44	6	0	-6.785253	-0.456373	-0.780052
45	1	0	-7.867862	-0.250798	-0.761194
46	1	0	-6.438865	-0.398307	-1.823487
47	1	0	-6.620311	-1.482324	-0.416186
48	6	0	-6.294231	2.001334	-0.429629
49	1	0	-5.773330	2.750649	0.186361
50	1	0	-5.938888	2.099462	-1.467025
51	1	0	-7.373324	2.224318	-0.408761

1b

Sum of electronic and zero-point Energies= -610.462499
Sum of electronic and thermal Energies= -610.449236
Sum of electronic and thermal Enthalpies= -610.448292
Sum of electronic and thermal Free Energies= -610.502909

Standard orientation:

Center Number	Atomic Number	Atomic Type	Coordinates (Angstroms) X	Y	Z
1	8	0	-5.424461	0.728972	-0.000071
2	6	0	-4.702398	-0.249565	-0.000063
3	1	0	-5.127587	-1.282689	-0.000009
4	6	0	-3.225247	-0.187101	-0.000157
5	6	0	-2.527820	1.034492	-0.000194
6	6	0	-2.434304	-1.345742	-0.000182
7	7	0	-1.200578	1.096685	-0.000318
8	7	0	-1.103468	-1.305348	-0.000313
9	1	0	-2.900256	-2.338666	-0.000072
10	6	0	-0.536774	-0.081340	-0.000466
11	6	0	0.895568	-0.024861	-0.000973
12	6	0	2.115587	0.018387	-0.002392
13	6	0	3.586030	0.060837	-0.001502
14	6	0	4.114302	-0.861732	-1.125053
15	1	0	5.216081	-0.843197	-1.128126
16	1	0	3.783997	-1.900575	-0.971555
17	1	0	3.758965	-0.526389	-2.111630
18	6	0	4.058357	1.512421	-0.241678
19	1	0	3.687998	2.184084	0.548059
20	1	0	5.159708	1.548125	-0.240441
21	1	0	3.700120	1.888886	-1.212250
22	6	0	4.095680	-0.440568	1.370901
23	1	0	3.726284	0.198523	2.187634
24	1	0	3.767003	-1.473753	1.561506
25	1	0	5.197325	-0.417600	1.382544
26	1	0	-3.079512	1.980751	-0.000111

5b(*RR*)

Sum of electronic and zero-point Energies= -1973.706765
Sum of electronic and thermal Energies= -1973.652502
Sum of electronic and thermal Enthalpies= -1973.651558
Sum of electronic and thermal Free Energies= -1973.800556

Standard orientation:

Center Number	Atomic Number	Atomic Type	Coordinates (Angstroms) X	Y	Z
1	8	0	1.411908	-1.904789	-0.921620
2	6	0	0.720670	-2.619431	0.088631
3	5	0	2.755290	-1.326346	-0.808733
4	6	0	3.329247	-1.091309	-2.331678
5	1	0	4.279267	-0.530175	-2.303415
6	1	0	3.562061	-2.101922	-2.719987
7	6	0	2.354658	-0.420293	-3.244415
8	6	0	2.456395	0.822197	-3.746466
9	1	0	1.451338	-0.998611	-3.478956
10	1	0	1.672670	1.251996	-4.379214
11	1	0	3.331169	1.454130	-3.550093
12	6	0	3.841064	-2.149397	0.124300
13	1	0	4.850180	-1.763010	-0.084812
14	6	0	3.651859	-2.247975	1.609268
15	6	0	4.426285	-1.698109	2.560292
16	1	0	2.793252	-2.837073	1.958146
17	1	0	4.196369	-1.826509	3.623222
18	1	0	5.315454	-1.106795	2.317869
19	1	0	3.824160	-3.170721	-0.304196
20	8	0	-1.432216	1.546063	-0.032079
21	6	0	-0.498157	2.491548	0.413550
22	5	0	-2.617622	1.066093	0.706790
23	6	0	-2.534821	1.061165	2.352695
24	1	0	-3.316320	0.346487	2.682001
25	1	0	-1.573639	0.646539	2.712382
26	6	0	-2.796378	2.364347	3.048015
27	6	0	-1.976957	2.992155	3.906905
28	1	0	-3.762587	2.838292	2.828326
29	1	0	-2.251083	3.947259	4.366359
30	1	0	-1.000940	2.575410	4.183908
31	6	0	-4.006927	1.692639	0.134210
32	1	0	-4.076656	2.723919	0.527396
33	6	0	-4.188877	1.741434	-1.357785
34	6	0	-4.668911	2.779148	-2.062012
35	1	0	-3.902946	0.845970	-1.925317
36	1	0	-4.765653	2.738184	-3.152151
37	1	0	-4.984550	3.710667	-1.576753
38	1	0	-4.860944	1.157315	0.588731
39	6	0	0.999787	-4.135856	-0.013839
40	1	0	0.680332	-4.474729	-1.015184
41	1	0	2.088260	-4.277682	0.030428
42	1	0	1.026769	-2.276366	1.092835
43	6	0	0.339669	-4.958449	1.058989
44	6	0	0.993109	-5.647133	2.004165
45	1	0	-0.757875	-4.984982	1.060259
46	1	0	0.456792	-6.227303	2.761527
47	1	0	2.087722	-5.665380	2.051876
48	1	0	-0.744004	2.853078	1.427325
49	6	0	-0.461574	3.723277	-0.538641
50	1	0	0.399194	4.355600	-0.266307
51	1	0	-0.275771	3.329180	-1.553699
52	6	0	-1.715763	4.545437	-0.529886
53	6	0	-1.788039	5.822121	-0.131048
54	1	0	-2.617857	4.040677	-0.890391

55	1	0	-2.732523	6.375194	-0.152653
56	1	0	-0.906878	6.367306	0.227338
57	6	0	-0.759244	-2.272449	-0.051742
58	6	0	-1.702427	-2.996270	-0.795474
59	6	0	-1.241081	-1.082991	0.483161
60	7	0	-2.984157	-2.641017	-0.857257
61	1	0	-1.430282	-3.905848	-1.339704
62	7	0	-2.495803	-0.630232	0.306095
63	1	0	-0.593743	-0.427341	1.061988
64	6	0	-3.373294	-1.491724	-0.285156
65	6	0	-4.779945	-1.246734	-0.301519
66	6	0	-5.998862	-1.198059	-0.343236
67	6	0	-7.465584	-1.112912	-0.402528
68	6	0	-7.942550	0.150697	0.348454
69	1	0	-7.633368	0.124457	1.404767
70	1	0	-7.534355	1.062587	-0.112892
71	1	0	-9.042377	0.205967	0.311125
72	6	0	-8.063631	-2.379034	0.255317
73	1	0	-9.163315	-2.331701	0.206285
74	1	0	-7.730837	-3.290296	-0.264905
75	1	0	-7.766571	-2.453475	1.312834
76	6	0	-7.894501	-1.038205	-1.887625
77	1	0	-7.466856	-0.149489	-2.376328
78	1	0	-7.566304	-1.932288	-2.439534
79	1	0	-8.992921	-0.974906	-1.947485
80	6	0	0.910830	1.911993	0.467118
81	6	0	1.276374	0.736540	-0.183658
82	6	0	1.950366	2.547592	1.163067
83	7	0	2.532641	0.243587	-0.163672
84	1	0	0.555259	0.130012	-0.727029
85	7	0	3.216462	2.150187	1.073867
86	1	0	1.755940	3.423345	1.792551
87	6	0	3.499783	1.043559	0.373663
88	6	0	4.888331	0.786326	0.155447
89	6	0	6.091918	0.753281	-0.046613
90	6	0	7.541131	0.695862	-0.293897
91	6	0	7.797609	0.074945	-1.686323
92	1	0	7.324615	0.673726	-2.479824
93	1	0	8.881948	0.039021	-1.878583
94	1	0	7.401676	-0.950584	-1.742903
95	6	0	8.112539	2.131935	-0.241223
96	1	0	7.934896	2.592219	0.742864
97	1	0	9.199218	2.102297	-0.421077
98	1	0	7.650717	2.769426	-1.010836
99	6	0	8.205858	-0.172928	0.800693
100	1	0	8.030818	0.248787	1.802288
101	1	0	7.818385	-1.202884	0.781791
102	1	0	9.293043	-0.209228	0.624822

5b(*RS*)

Sum of electronic and zero-point Energies= -1973.713851
Sum of electronic and thermal Energies= -1973.659564
Sum of electronic and thermal Enthalpies= -1973.658620
Sum of electronic and thermal Free Energies= -1973.806706

Standard orientation:

Center Number	Atomic Number	Atomic Type	Coordinates (Angstroms) X	Y	Z
1	8	0	-1.300242	1.940680	-0.245554
2	6	0	-0.582618	2.610792	0.772003
3	5	0	-2.660828	1.388600	-0.201988
4	6	0	-3.451234	1.924672	-1.538558
5	1	0	-4.465215	1.505191	-1.629155
6	1	0	-3.573233	3.011658	-1.356589
7	6	0	-2.692801	1.728167	-2.813101
8	6	0	-3.087373	1.008336	-3.876344
9	1	0	-1.701121	2.198425	-2.846481
10	1	0	-2.448901	0.890160	-4.758428
11	1	0	-4.064011	0.510266	-3.907246
12	6	0	-3.505424	1.616305	1.196405
13	1	0	-4.582182	1.493897	0.992777
14	6	0	-3.131871	0.778415	2.382216
15	6	0	-3.909706	-0.129248	2.994338
16	1	0	-2.112980	0.907371	2.774330
17	1	0	-3.544216	-0.716037	3.843379
18	1	0	-4.939656	-0.318978	2.671637
19	1	0	-3.369232	2.685017	1.453441
20	8	0	1.226643	-1.617985	0.753576
21	6	0	0.573764	-2.584362	-0.036782
22	5	0	2.645007	-1.232359	0.690229
23	6	0	3.277801	-1.399689	2.199225
24	1	0	2.556716	-0.928027	2.896732
25	1	0	3.261681	-2.481681	2.426065
26	6	0	4.643138	-0.852422	2.464302
27	6	0	5.754305	-1.564907	2.714667
28	1	0	4.738270	0.242396	2.438422
29	1	0	6.721571	-1.080637	2.884415
30	1	0	5.736805	-2.660204	2.764061
31	6	0	3.535095	-1.938129	-0.481511
32	1	0	3.515824	-3.025126	-0.275132
33	6	0	3.205647	-1.706337	-1.927915
34	6	0	2.987485	-2.649763	-2.859353
35	1	0	3.156550	-0.659675	-2.257400
36	1	0	2.758841	-2.388571	-3.897731
37	1	0	3.028390	-3.719034	-2.618696
38	1	0	4.584115	-1.636097	-0.311572
39	6	0	-0.882114	4.125743	0.763565
40	1	0	-0.623167	4.525903	-0.232344
41	1	0	-1.969600	4.245278	0.877296
42	1	0	-0.838462	2.219162	1.772485
43	6	0	-0.172231	4.891982	1.846134
44	6	0	-0.775138	5.467582	2.894451
45	1	0	0.920066	4.966975	1.765138
46	1	0	-0.204975	6.007063	3.657086
47	1	0	-1.862242	5.426939	3.027159
48	6	0	0.879515	2.261925	0.501411
49	6	0	1.768657	3.017885	-0.272688
50	6	0	1.343980	0.997193	0.853849
51	7	0	2.991765	2.586208	-0.581133
52	1	0	1.502412	4.009525	-0.650895
53	7	0	2.514954	0.487313	0.426827
54	1	0	0.740742	0.311232	1.446946

55	6	0	3.352262	1.343949	-0.230869
56	6	0	4.686934	0.967815	-0.572621
57	6	0	5.851459	0.792440	-0.893239
58	6	0	7.252300	0.561506	-1.276004
59	6	0	7.543705	-0.956400	-1.271417
60	1	0	7.375104	-1.388025	-0.273189
61	1	0	6.902194	-1.484714	-1.993084
62	1	0	8.595346	-1.127912	-1.552184
63	6	0	8.167477	1.278630	-0.254370
64	1	0	9.221102	1.117825	-0.533837
65	1	0	7.972816	2.362055	-0.240336
66	1	0	8.012819	0.882798	0.760912
67	6	0	7.486671	1.143555	-2.689569
68	1	0	6.839105	0.651460	-3.431449
69	1	0	7.281565	2.224913	-2.711522
70	1	0	8.536347	0.981794	-2.983103
71	6	0	-0.869369	-2.134136	-0.247391
72	6	0	-1.189296	-0.777859	-0.274567
73	6	0	-1.965381	-2.991818	-0.414801
74	7	0	-2.443837	-0.304792	-0.360514
75	1	0	-0.426845	-0.008251	-0.222543
76	7	0	-3.214940	-2.543100	-0.536172
77	1	0	-1.842883	-4.077656	-0.454236
78	6	0	-3.445417	-1.226407	-0.477400
79	6	0	-4.818770	-0.834048	-0.543919
80	6	0	-6.023369	-0.650690	-0.615759
81	6	0	-7.472608	-0.412775	-0.705408
82	6	0	-7.729996	0.908465	-1.465608
83	1	0	-7.319047	0.865490	-2.485940
84	1	0	-8.815313	1.085299	-1.536645
85	1	0	-7.273927	1.762849	-0.942584
86	6	0	-8.127348	-1.593501	-1.459193
87	1	0	-7.951269	-2.544365	-0.933171
88	1	0	-9.214739	-1.429023	-1.527609
89	1	0	-7.724809	-1.682325	-2.480016
90	6	0	-8.053582	-0.317355	0.725690
91	1	0	-7.870503	-1.244493	1.290219
92	1	0	-7.607837	0.522749	1.279959
93	1	0	-9.141900	-0.154863	0.667396
94	1	0	1.042548	-2.655341	-1.035131
95	6	0	0.671262	-3.983430	0.618890
96	1	0	1.749714	-4.207124	0.691195
97	1	0	0.239797	-4.747038	-0.049316
98	6	0	0.055071	-4.058924	1.987363
99	6	0	-0.787921	-5.011295	2.407217
100	1	0	0.345959	-3.257775	2.677045
101	1	0	-1.188431	-5.010653	3.425843
102	1	0	-1.105178	-5.833424	1.755162

7b

Sum of electronic and zero-point Energies= -2584.188244
Sum of electronic and thermal Energies= -2584.117925
Sum of electronic and thermal Enthalpies= -2584.116981
Sum of electronic and thermal Free Energies= -2584.310873

Standard orientation:

Center Number	Atomic Number	Atomic Type	Coordinates (Angstroms) X	Y	Z
1	8	0	-2.500556	1.175476	1.976587
2	6	0	-3.593155	2.056810	2.089873
3	5	0	-1.135425	1.697568	1.739652
4	6	0	-0.589802	2.594458	3.013042
5	1	0	0.351386	3.115440	2.775680
6	1	0	-1.353658	3.388772	3.139416
7	6	0	-0.451825	1.840310	4.297443
8	6	0	0.672375	1.678944	5.014966
9	1	0	-1.363274	1.345031	4.660199
10	1	0	0.687127	1.079375	5.931313
11	1	0	1.619898	2.142256	4.715589
12	6	0	-1.003678	2.489538	0.299867
13	1	0	-0.030519	2.995882	0.199651
14	6	0	-1.277153	1.658688	-0.913521
15	6	0	-0.427015	1.414736	-1.925434
16	1	0	-2.268000	1.187044	-0.957279
17	1	0	-0.708466	0.772797	-2.766589
18	1	0	0.577009	1.854083	-1.956003
19	1	0	-1.761560	3.296882	0.362314
20	8	0	-0.324122	-4.519404	1.380936
21	6	0	-0.964356	-3.425995	2.045593
22	5	0	-0.175360	-4.764020	0.041174
23	6	0	-0.664222	-3.771502	-1.109991
24	1	0	-1.643600	-4.181582	-1.436726
25	1	0	-0.865099	-2.755520	-0.735793
26	6	0	0.253404	-3.712809	-2.300971
27	6	0	0.951644	-2.639474	-2.697275
28	1	0	0.360742	-4.643536	-2.873292
29	1	0	1.611622	-2.681010	-3.569540
30	1	0	0.888141	-1.680122	-2.171328
31	6	0	0.474493	-6.186241	-0.284056
32	1	0	0.845981	-6.645059	0.646207
33	6	0	-0.527802	-7.090581	-0.951097
34	6	0	-1.124939	-8.150670	-0.385955
35	1	0	-0.804371	-6.829549	-1.981755
36	1	0	-1.862743	-8.747328	-0.931584
37	1	0	-0.893479	-8.464321	0.638427
38	1	0	1.339229	-6.037890	-0.957461
39	6	0	-4.344558	1.742302	3.412186
40	1	0	-4.654699	0.682529	3.366568
41	1	0	-3.615181	1.839159	4.231102
42	1	0	-3.252067	3.106910	2.147028
43	6	0	-5.538338	2.614762	3.679701
44	6	0	-5.618439	3.519769	4.664073
45	1	0	-6.397307	2.490988	3.007637
46	1	0	-6.515937	4.129057	4.809589
47	1	0	-4.792266	3.682045	5.365847
48	8	0	3.592303	-3.926913	0.313968
49	6	0	4.594497	-3.906066	-0.377020
50	1	0	4.990884	-4.842796	-0.837350
51	1	0	-0.823717	-3.649980	3.117139
52	6	0	-2.483475	-3.395153	1.788147
53	1	0	-2.923183	-2.615999	2.429666
54	1	0	-2.666825	-3.099828	0.740436

55	6	0	-3.156617	-4.709473	2.078958
56	6	0	-4.092161	-4.888907	3.019186
57	1	0	-2.848377	-5.563795	1.464696
58	1	0	-4.555518	-5.866505	3.183370
59	1	0	-4.435476	-4.066360	3.657054
60	6	0	5.381568	-2.694311	-0.679192
61	6	0	5.016233	-1.410804	-0.228626
62	6	0	6.554008	-2.769763	-1.446734
63	7	0	5.744099	-0.333373	-0.511034
64	1	0	4.107544	-1.261907	0.367338
65	7	0	7.293070	-1.701659	-1.736971
66	1	0	6.903075	-3.734341	-1.835317
67	6	0	6.857864	-0.520041	-1.254196
68	6	0	7.645607	0.638133	-1.558061
69	6	0	8.317901	1.622948	-1.820094
70	6	0	9.137814	2.802213	-2.138589
71	6	0	9.504281	2.767688	-3.640791
72	1	0	10.124625	3.644040	-3.888555
73	1	0	10.072336	1.857740	-3.888033
74	1	0	8.600312	2.793989	-4.268665
75	6	0	8.339191	4.085608	-1.817663
76	1	0	8.065667	4.124245	-0.751843
77	1	0	8.951590	4.971127	-2.052053
78	1	0	7.415403	4.134881	-2.414590
79	6	0	10.426563	2.754564	-1.283165
80	1	0	10.189149	2.771819	-0.208362
81	1	0	11.009010	1.845125	-1.496417
82	1	0	11.052186	3.631365	-1.515523
83	6	0	-4.512621	1.938831	0.885216
84	6	0	-5.124853	3.049368	0.288792
85	6	0	-4.827584	0.704256	0.298425
86	7	0	-5.955630	2.954353	-0.752465
87	1	0	-4.929866	4.060109	0.667232
88	7	0	-5.655298	0.581233	-0.740757
89	1	0	-4.381446	-0.220289	0.679851
90	6	0	-6.192869	1.717604	-1.225204
91	6	0	-7.092054	1.597946	-2.342672
92	6	0	-7.855265	1.497615	-3.289347
93	6	0	-8.779870	1.377298	-4.429576
94	6	0	-8.436760	0.101010	-5.231067
95	1	0	-7.408127	0.144730	-5.621308
96	1	0	-8.529209	-0.797476	-4.601447
97	1	0	-9.127604	0.000628	-6.083953
98	6	0	-8.635531	2.622728	-5.333820
99	1	0	-9.326724	2.543510	-6.188634
100	1	0	-8.873441	3.543268	-4.778690
101	1	0	-7.609804	2.710197	-5.724342
102	6	0	-10.227477	1.285552	-3.891890
103	1	0	-10.352291	0.406340	-3.240995
104	1	0	-10.492119	2.184774	-3.314368
105	1	0	-10.930207	1.195406	-4.736209
106	6	0	-0.246430	-2.109243	1.785631
107	6	0	-0.893014	-0.876587	1.838294
108	6	0	1.133464	-2.054007	1.540246
109	7	0	-0.246893	0.295156	1.667294
110	1	0	-1.959940	-0.762559	2.020346
111	7	0	1.780928	-0.902010	1.379779
112	1	0	1.741194	-2.958705	1.454242
113	6	0	1.100169	0.249264	1.448707
114	6	0	1.873309	1.436670	1.282332
115	6	0	2.677375	2.342184	1.127966
116	6	0	3.635935	3.439249	0.930376
117	6	0	3.043336	4.754882	1.483125
118	1	0	2.840519	4.676499	2.562338
119	1	0	3.762603	5.574669	1.325975
120	1	0	2.104772	5.015874	0.970491

121	6	0	4.948522	3.092220	1.672015
122	1	0	5.382888	2.159166	1.283090
123	1	0	5.677028	3.905933	1.524641
124	1	0	4.772862	2.975887	2.752776
125	6	0	3.914409	3.576449	-0.586429
126	1	0	4.337180	2.645115	-0.992179
127	1	0	2.992908	3.817122	-1.138687
128	1	0	4.639620	4.389915	-0.749703

TS1b(*S*)

Sum of electronic and zero-point Energies= -2584.184525
Sum of electronic and thermal Energies= -2584.114137
Sum of electronic and thermal Enthalpies= -2584.113193
Sum of electronic and thermal Free Energies= -2584.306363

Standard orientation:

Center Number	Atomic Number	Atomic Type	Coordinates (Angstroms) X	Y	Z
1	8	0	-2.354077	-1.339223	-2.000725
2	6	0	-3.485588	-2.171709	-1.907284
3	5	0	-0.990666	-1.912313	-1.945355
4	6	0	-0.670366	-2.891183	-3.237902
5	1	0	0.284704	-3.427646	-3.113119
6	1	0	-1.462164	-3.667051	-3.201722
7	6	0	-0.707569	-2.215055	-4.571197
8	6	0	0.303455	-2.126452	-5.451659
9	1	0	-1.652080	-1.716098	-4.828889
10	1	0	0.197026	-1.580244	-6.394889
11	1	0	1.274524	-2.600193	-5.264759
12	6	0	-0.680344	-2.660927	-0.511595
13	1	0	0.263009	-3.228845	-0.540483
14	6	0	-0.717456	-1.791987	0.705509
15	6	0	0.272823	-1.626905	1.598752
16	1	0	-1.647662	-1.230563	0.866520
17	1	0	0.158883	-0.964272	2.462868
18	1	0	1.227200	-2.158565	1.508119
19	1	0	-1.485647	-3.419731	-0.428512
20	8	0	0.022545	4.175156	-1.866113
21	6	0	-0.601632	3.189679	-2.680639
22	5	0	0.121506	4.290440	-0.473344
23	6	0	-0.783607	3.384837	0.519795
24	1	0	-1.816805	3.759799	0.374418
25	1	0	-0.784866	2.327723	0.206171
26	6	0	-0.449279	3.493180	1.974254
27	6	0	0.066783	2.518514	2.741596
28	1	0	-0.629289	4.472297	2.438716
29	1	0	0.297677	2.683629	3.798949
30	1	0	0.255918	1.515270	2.343850
31	6	0	0.563741	5.786117	-0.042771
32	1	0	1.289345	6.167853	-0.781281
33	6	0	-0.618213	6.709793	0.036359
34	6	0	-0.911912	7.700316	-0.820251
35	1	0	-1.312923	6.531450	0.869493
36	1	0	-1.811046	8.313022	-0.699260
37	1	0	-0.265252	7.933867	-1.673850
38	1	0	1.062448	5.780167	0.943891
39	6	0	-4.411035	-1.887939	-3.122184
40	1	0	-4.661913	-0.812124	-3.098212
41	1	0	-3.817531	-2.070019	-4.031325
42	1	0	-3.199676	-3.238645	-1.955688
43	6	0	-5.669402	-2.708746	-3.155564
44	6	0	-5.922439	-3.684500	-4.037912
45	1	0	-6.422113	-2.484496	-2.388843
46	1	0	-6.857338	-4.252955	-4.010292
47	1	0	-5.207588	-3.947889	-4.825987
48	8	0	1.853179	3.310528	-0.153718
49	6	0	2.559965	3.411339	0.851936
50	1	0	2.398316	4.234375	1.574365
51	1	0	-0.347850	3.493583	-3.711777
52	6	0	-2.139041	3.194463	-2.592484
53	1	0	-2.525405	2.460154	-3.317868
54	1	0	-2.451276	2.856505	-1.589698

55	6	0	-2.734499	4.544499	-2.883169
56	6	0	-3.603433	4.794801	-3.870415
57	1	0	-2.416141	5.365042	-2.229521
58	1	0	-4.008475	5.798501	-4.032777
59	1	0	-3.947664	4.007965	-4.551651
60	6	0	3.667115	2.505564	1.162992
61	6	0	4.043428	1.416537	0.348855
62	6	0	4.429998	2.696901	2.329579
63	7	0	5.050879	0.613425	0.671462
64	1	0	3.512459	1.195054	-0.583434
65	7	0	5.442285	1.903165	2.662352
66	1	0	4.202057	3.523962	3.012711
67	6	0	5.712699	0.883970	1.819474
68	6	0	6.793005	0.012874	2.168189
69	6	0	7.711217	-0.736819	2.461425
70	6	0	8.818832	-1.639934	2.806774
71	6	0	9.247584	-1.390263	4.270224
72	1	0	10.079905	-2.063217	4.531633
73	1	0	9.583228	-0.351364	4.411445
74	1	0	8.414778	-1.582707	4.964055
75	6	0	8.346554	-3.102482	2.632889
76	1	0	8.032828	-3.295878	1.595483
77	1	0	9.173908	-3.786687	2.880530
78	1	0	7.499776	-3.326300	3.299856
79	6	0	10.003229	-1.354240	1.852083
80	1	0	9.716155	-1.524229	0.803095
81	1	0	10.351684	-0.315033	1.953985
82	1	0	10.840125	-2.027349	2.098205
83	6	0	-4.211354	-1.953469	-0.589552
84	6	0	-4.766269	-3.005537	0.151270
85	6	0	-4.391632	-0.678514	-0.032811
86	7	0	-5.428152	-2.823309	1.296844
87	1	0	-4.666006	-4.041204	-0.195316
88	7	0	-5.050559	-0.468593	1.108234
89	1	0	-3.976632	0.206024	-0.527611
90	6	0	-5.548795	-1.555348	1.729229
91	6	0	-6.266590	-1.341438	2.958381
92	6	0	-6.876779	-1.161049	3.999489
93	6	0	-7.607367	-0.943362	5.259793
94	6	0	-8.300544	0.437427	5.213779
95	1	0	-7.563248	1.244969	5.085499
96	1	0	-9.019454	0.490753	4.381497
97	1	0	-8.846393	0.611165	6.155402
98	6	0	-6.601061	-0.985083	6.434087
99	1	0	-7.135397	-0.822874	7.384367
100	1	0	-6.092072	-1.960063	6.483825
101	1	0	-5.836441	-0.200178	6.327231
102	6	0	-8.663221	-2.058489	5.435221
103	1	0	-9.388277	-2.049507	4.606608
104	1	0	-8.187563	-3.050915	5.467135
105	1	0	-9.212527	-1.905532	6.378419
106	6	0	0.034625	1.821017	-2.485533
107	6	0	-0.665691	0.639877	-2.266921
108	6	0	1.427711	1.687851	-2.561502
109	7	0	-0.049120	-0.552896	-2.100404
110	1	0	-1.751679	0.579841	-2.226663
111	7	0	2.047496	0.528496	-2.370077
112	1	0	2.059056	2.554630	-2.774698
113	6	0	1.313578	-0.567683	-2.127685
114	6	0	2.065457	-1.758224	-1.892549
115	6	0	2.892245	-2.632666	-1.686787
116	6	0	3.895435	-3.675869	-1.428073
117	6	0	3.658146	-4.863258	-2.389801
118	1	0	3.734947	-4.543480	-3.440446
119	1	0	4.418413	-5.639575	-2.207013
120	1	0	2.663588	-5.308467	-2.233235

121	6	0	5.300751	-3.071929	-1.659304
122	1	0	5.470531	-2.207410	-0.999116
123	1	0	6.068423	-3.833548	-1.446745
124	1	0	5.420152	-2.740315	-2.702372
125	6	0	3.763569	-4.150617	0.038587
126	1	0	3.933700	-3.318625	0.738785
127	1	0	2.766769	-4.575214	0.232371
128	1	0	4.516704	-4.930219	0.236755

8b(*S*)

Sum of electronic and zero-point Energies= -2584.185000
Sum of electronic and thermal Energies= -2584.114492
Sum of electronic and thermal Enthalpies= -2584.113548
Sum of electronic and thermal Free Energies= -2584.305180

Standard orientation:

Center Number	Atomic Number	Atomic Type	Coordinates (Angstroms) X	Y	Z
1	8	0	-2.313130	-1.235196	-2.081684
2	6	0	-3.446542	-2.066826	-2.005446
3	5	0	-0.951984	-1.815080	-2.088486
4	6	0	-0.663294	-2.718624	-3.441972
5	1	0	0.293041	-3.262684	-3.369966
6	1	0	-1.455827	-3.494637	-3.433211
7	6	0	-0.728387	-1.966212	-4.732663
8	6	0	0.260515	-1.836195	-5.632849
9	1	0	-1.673953	-1.444481	-4.935538
10	1	0	0.135873	-1.235263	-6.539878
11	1	0	1.231153	-2.328311	-5.498549
12	6	0	-0.614939	-2.648356	-0.708257
13	1	0	0.325195	-3.216352	-0.790478
14	6	0	-0.624203	-1.855083	0.559780
15	6	0	0.382768	-1.751390	1.443730
16	1	0	-1.547143	-1.298487	0.771360
17	1	0	0.287684	-1.144152	2.349715
18	1	0	1.330917	-2.283535	1.304399
19	1	0	-1.421542	-3.408470	-0.654406
20	8	0	0.072483	4.250664	-1.682557
21	6	0	-0.547525	3.324601	-2.552639
22	5	0	0.265133	4.230272	-0.263587
23	6	0	-0.779258	3.329179	0.658396
24	1	0	-1.765675	3.811217	0.538178
25	1	0	-0.847024	2.306339	0.253001
26	6	0	-0.404338	3.295090	2.094114
27	6	0	0.265305	2.297337	2.709140
28	1	0	-0.634453	4.194391	2.681449
29	1	0	0.549630	2.363899	3.764152
30	1	0	0.501003	1.362030	2.190266
31	6	0	0.561285	5.753599	0.253647
32	1	0	1.327184	6.198098	-0.407149
33	6	0	-0.658604	6.622639	0.274728
34	6	0	-0.920613	7.650198	-0.548185
35	1	0	-1.413303	6.372593	1.034226
36	1	0	-1.852233	8.220421	-0.473614
37	1	0	-0.215141	7.955974	-1.329370
38	1	0	0.982212	5.738668	1.277664
39	6	0	-4.401439	-1.709580	-3.176886
40	1	0	-4.648866	-0.636792	-3.081719
41	1	0	-3.831716	-1.836626	-4.110282
42	1	0	-3.168234	-3.130522	-2.121228
43	6	0	-5.662661	-2.524748	-3.227812
44	6	0	-5.950267	-3.429932	-4.172518
45	1	0	-6.387856	-2.357863	-2.420904
46	1	0	-6.886244	-3.997018	-4.155955
47	1	0	-5.263702	-3.634012	-5.002258
48	8	0	1.681070	3.371802	-0.020902
49	6	0	2.362853	3.419472	1.028171
50	1	0	2.207068	4.237030	1.747343
51	1	0	-0.293877	3.685470	-3.566593
52	6	0	-2.087604	3.321486	-2.473949
53	1	0	-2.473717	2.620343	-3.232204
54	1	0	-2.403679	2.941458	-1.487525

55	6	0	-2.679404	4.683732	-2.705371
56	6	0	-3.574281	4.973978	-3.658197
57	1	0	-2.329895	5.478232	-2.035880
58	1	0	-3.972850	5.986467	-3.776998
59	1	0	-3.947121	4.212946	-4.353762
60	6	0	3.450279	2.496999	1.309755
61	6	0	3.847985	1.445554	0.454953
62	6	0	4.194737	2.638735	2.498351
63	7	0	4.847401	0.628707	0.764251
64	1	0	3.346254	1.267912	-0.501453
65	7	0	5.196761	1.829640	2.816934
66	1	0	3.955536	3.438486	3.209308
67	6	0	5.481751	0.843114	1.939122
68	6	0	6.544248	-0.051851	2.277219
69	6	0	7.443774	-0.828599	2.558176
70	6	0	8.530629	-1.761034	2.890166
71	6	0	8.564530	-1.977701	4.420562
72	1	0	9.381349	-2.671792	4.675473
73	1	0	8.736305	-1.028258	4.950451
74	1	0	7.617664	-2.409472	4.779637
75	6	0	8.284662	-3.105191	2.167247
76	1	0	8.252476	-2.966455	1.075585
77	1	0	9.100360	-3.806406	2.405717
78	1	0	7.333456	-3.557425	2.487927
79	6	0	9.869146	-1.144298	2.415798
80	1	0	9.864018	-0.976099	1.327980
81	1	0	10.061403	-0.183130	2.916658
82	1	0	10.693317	-1.834330	2.657851
83	6	0	-4.136984	-1.919978	-0.659129
84	6	0	-4.666122	-3.011243	0.042538
85	6	0	-4.308702	-0.675510	-0.034415
86	7	0	-5.298903	-2.890677	1.212610
87	1	0	-4.569617	-4.027661	-0.357961

88	7	0	-4.938070	-0.527169	1.132587
89	1	0	-3.912027	0.235633	-0.494667
90	6	0	-5.414674	-1.646787	1.710889
91	6	0	-6.100281	-1.498072	2.967735
92	6	0	-6.682318	-1.369672	4.032362
93	6	0	-7.387562	-1.217884	5.316588
94	6	0	-7.154276	0.209462	5.861145
95	1	0	-6.082127	0.398777	6.025312
96	1	0	-7.534699	0.966913	5.158436
97	1	0	-7.679670	0.332093	6.822230
98	6	0	-6.841004	-2.260496	6.319096
99	1	0	-7.365423	-2.158402	7.283137
100	1	0	-6.994685	-3.285030	5.946487
101	1	0	-5.763743	-2.113852	6.493092
102	6	0	-8.899402	-1.453168	5.089652
103	1	0	-9.309578	-0.724030	4.373852
104	1	0	-9.087401	-2.465309	4.699297
105	1	0	-9.438436	-1.343053	6.044689
106	6	0	0.079299	1.939252	-2.451327
107	6	0	-0.621259	0.750556	-2.276218
108	6	0	1.468930	1.802545	-2.568296
109	7	0	-0.007225	-0.452359	-2.187156
110	1	0	-1.706241	0.693794	-2.211803
111	7	0	2.088055	0.633016	-2.449862
112	1	0	2.100164	2.677205	-2.751457
113	6	0	1.354084	-0.471540	-2.247077
114	6	0	2.107027	-1.673843	-2.085380
115	6	0	2.939170	-2.554229	-1.933179
116	6	0	3.953329	-3.599849	-1.733288
117	6	0	3.796748	-4.679351	-2.829669
118	1	0	3.919356	-4.244840	-3.833719
119	1	0	4.566467	-5.455504	-2.690927
120	1	0	2.807172	-5.158721	-2.776066

121	6	0	5.351964	-2.945040	-1.823496
122	1	0	5.462181	-2.148278	-1.071657
123	1	0	6.128117	-3.707033	-1.645864
124	1	0	5.517486	-2.505903	-2.819429
125	6	0	3.760332	-4.235414	-0.336272
126	1	0	3.881803	-3.483774	0.458617
127	1	0	2.763157	-4.691146	-0.239212
128	1	0	4.516995	-5.022495	-0.187256

TS2b(*S*)

Sum of electronic and zero-point Energies= -2584.182525
Sum of electronic and thermal Energies= -2584.113144
Sum of electronic and thermal Enthalpies= -2584.112200
Sum of electronic and thermal Free Energies= -2584.303519

Standard orientation:

Center Number	Atomic Number	Atomic Type	Coordinates (Angstroms) X	Y	Z
1	8	0	-2.350701	-1.264875	-2.015549
2	6	0	-3.510722	-2.059485	-1.943837
3	5	0	-1.006587	-1.882463	-1.966414
4	6	0	-0.720011	-2.869290	-3.260204
5	1	0	0.221205	-3.430767	-3.140052
6	1	0	-1.530604	-3.625428	-3.220323
7	6	0	-0.746226	-2.192990	-4.593875
8	6	0	0.258467	-2.141970	-5.484354
9	1	0	-1.675085	-1.661400	-4.842953
10	1	0	0.162209	-1.593834	-6.427562
11	1	0	1.214004	-2.649202	-5.305692
12	6	0	-0.715476	-2.645575	-0.535747
13	1	0	0.218649	-3.228655	-0.564777
14	6	0	-0.745804	-1.786211	0.687706
15	6	0	0.241558	-1.639805	1.587895
16	1	0	-1.670112	-1.215518	0.849711
17	1	0	0.128284	-0.984937	2.457946
18	1	0	1.189911	-2.182416	1.499966
19	1	0	-1.534310	-3.390896	-0.463496
20	8	0	0.149087	4.168828	-1.798339
21	6	0	-0.453726	3.215303	-2.647076
22	5	0	0.433196	4.191725	-0.393587
23	6	0	-0.709545	3.317410	0.588691
24	1	0	-1.640557	3.893924	0.494951
25	1	0	-0.808214	2.322395	0.133127
26	6	0	-0.185296	3.275288	1.932359
27	6	0	0.759298	2.366803	2.350182
28	1	0	-0.395861	4.129203	2.588989
29	1	0	1.178456	2.420912	3.358799
30	1	0	0.899138	1.425223	1.812380
31	6	0	0.642594	5.746873	0.076214
32	1	0	1.400623	6.189142	-0.595782
33	6	0	-0.599644	6.582129	0.056923
34	6	0	-0.877930	7.579181	-0.797334
35	1	0	-1.356354	6.337341	0.816324
36	1	0	-1.824139	8.127765	-0.748869
37	1	0	-0.171480	7.880223	-1.579393
38	1	0	1.060626	5.785557	1.100703
39	6	0	-4.432984	-1.694932	-3.138896
40	1	0	-4.650307	-0.613763	-3.066746
41	1	0	-3.850593	-1.854426	-4.059352
42	1	0	-3.263599	-3.133204	-2.034475
43	6	0	-5.716766	-2.473571	-3.199392
44	6	0	-6.014328	-3.385566	-4.134430
45	1	0	-6.450724	-2.271925	-2.408518
46	1	0	-6.966782	-3.924708	-4.125606
47	1	0	-5.319716	-3.623412	-4.948300
48	8	0	1.723804	3.370948	-0.112126
49	6	0	2.304333	3.375028	1.035286
50	1	0	2.273199	4.286851	1.644312
51	1	0	-0.169099	3.530805	-3.668020
52	6	0	-1.995594	3.246712	-2.604315
53	1	0	-2.381059	2.514649	-3.332971
54	1	0	-2.340328	2.926192	-1.605772

55	6	0	-2.551773	4.607146	-2.919603
56	6	0	-3.417098	4.866076	-3.908095
57	1	0	-2.197925	5.427136	-2.283935
58	1	0	-3.789068	5.879359	-4.089746
59	1	0	-3.791164	4.077608	-4.571689
60	6	0	3.433144	2.475598	1.304509
61	6	0	3.818522	1.407006	0.472042
62	6	0	4.232893	2.674519	2.446023
63	7	0	4.851567	0.619873	0.761913
64	1	0	3.284347	1.187449	-0.456451
65	7	0	5.268095	1.898198	2.746293
66	1	0	4.016227	3.498934	3.136428
67	6	0	5.533121	0.884897	1.895266
68	6	0	6.629109	0.018730	2.216898
69	6	0	7.555890	-0.731535	2.478774
70	6	0	8.674349	-1.636104	2.788121
71	6	0	8.911587	-1.650684	4.315329
72	1	0	9.750402	-2.324546	4.553180
73	1	0	9.158289	-0.643954	4.686242
74	1	0	8.017993	-2.007612	4.850337
75	6	0	8.326012	-3.058273	2.291469
76	1	0	8.151143	-3.064868	1.204443
77	1	0	9.160674	-3.742363	2.514385
78	1	0	7.421897	-3.440479	2.790271
79	6	0	9.941877	-1.123171	2.063535
80	1	0	9.792330	-1.100942	0.973115
81	1	0	10.206313	-0.108676	2.399320
82	1	0	10.787026	-1.794547	2.285583
83	6	0	-4.220518	-1.865959	-0.613635
84	6	0	-4.788877	-2.928050	0.101973
85	6	0	-4.370450	-0.604538	-0.018578
86	7	0	-5.435461	-2.766324	1.259287
87	1	0	-4.712561	-3.954782	-0.275737
88	7	0	-5.012836	-0.415006	1.135465
89	1	0	-3.942991	0.285899	-0.491998
90	6	0	-5.526221	-1.509646	1.730013
91	6	0	-6.226740	-1.318821	2.972915
92	6	0	-6.821720	-1.161678	4.026498
93	6	0	-7.539451	-0.968842	5.298209
94	6	0	-6.685989	-0.077140	6.229468
95	1	0	-5.713835	-0.547277	6.444355
96	1	0	-6.500783	0.907385	5.772742
97	1	0	-7.215727	0.076137	7.183755
98	6	0	-7.779814	-2.343724	5.961705
99	1	0	-8.317259	-2.208581	6.914417
100	1	0	-8.384086	-2.995298	5.311700
101	1	0	-6.826444	-2.853384	6.170510
102	6	0	-8.894196	-0.279900	5.010796
103	1	0	-8.745258	0.701890	4.535299
104	1	0	-9.517365	-0.896513	4.344704
105	1	0	-9.440554	-0.129210	5.956070
106	6	0	0.137378	1.821163	-2.479254
107	6	0	-0.599059	0.661230	-2.263333
108	6	0	1.523354	1.639981	-2.580016
109	7	0	-0.021806	-0.554500	-2.120223
110	1	0	-1.685729	0.637640	-2.208256
111	7	0	2.106006	0.458127	-2.411846
112	1	0	2.181883	2.487670	-2.791716
113	6	0	1.338876	-0.615193	-2.168124
114	6	0	2.056185	-1.828540	-1.942353
115	6	0	2.866040	-2.717849	-1.732798
116	6	0	3.855951	-3.771174	-1.465105
117	6	0	3.669120	-4.920791	-2.482287
118	1	0	3.798699	-4.561243	-3.514707
119	1	0	4.420023	-5.704034	-2.290698
120	1	0	2.668260	-5.370611	-2.393144

121	6	0	5.268733	-3.156048	-1.604193
122	1	0	5.399930	-2.312106	-0.909405
123	1	0	6.028287	-3.921462	-1.376694
124	1	0	5.439571	-2.790094	-2.628382
125	6	0	3.653640	-4.302034	-0.026131
126	1	0	3.786240	-3.497376	0.713111
127	1	0	2.649723	-4.734941	0.101406
128	1	0	4.398172	-5.087899	0.179492

9b(*S*)

Sum of electronic and zero-point Energies= -2584.239758
Sum of electronic and thermal Energies= -2584.169348
Sum of electronic and thermal Enthalpies= -2584.168404
Sum of electronic and thermal Free Energies= -2584.361833

Standard orientation:

Center Number	Atomic Number	Atomic Type	Coordinates (Angstroms) X	Y	Z
1	8	0	-2.211720	2.203575	1.181463
2	6	0	-3.468067	2.726177	0.819101
3	5	0	-0.965882	2.803842	0.652378
4	6	0	-0.769313	4.366194	1.154336
5	1	0	0.049156	4.869701	0.615008
6	1	0	-1.700928	4.877329	0.836371
7	6	0	-0.595610	4.541060	2.629542
8	6	0	0.452976	5.106540	3.250571
9	1	0	-1.398780	4.130841	3.257555
10	1	0	0.510986	5.162721	4.342755
11	1	0	1.291545	5.537598	2.691064
12	6	0	-0.832870	2.652050	-0.981799
13	1	0	0.060693	3.169949	-1.364061
14	6	0	-0.882361	1.255150	-1.515445
15	6	0	0.083077	0.627108	-2.207798
16	1	0	-1.800461	0.692834	-1.299321
17	1	0	-0.041745	-0.404630	-2.552300
18	1	0	1.031253	1.114268	-2.461953
19	1	0	-1.702347	3.215477	-1.378653
20	8	0	1.279651	-1.902241	4.173628
21	6	0	0.235839	-0.955729	3.945222
22	5	0	1.790884	-2.816589	3.287864
23	6	0	0.341813	-6.673406	-0.612599
24	1	0	0.561806	-7.386150	-1.413258
25	1	0	-0.190916	-7.061935	0.262842
26	6	0	0.697206	-5.386056	-0.706120
27	6	0	0.424416	-4.345540	0.344263
28	1	0	1.224092	-5.045065	-1.606255
29	1	0	-0.123063	-3.494946	-0.099444
30	1	0	-0.208187	-4.766687	1.141610
31	6	0	2.934618	-3.788476	3.817794
32	1	0	3.756657	-3.839346	3.081884
33	6	0	3.475300	-3.444542	5.176003
34	6	0	4.748889	-3.132115	5.450511
35	1	0	2.745829	-3.442499	5.995583
36	1	0	5.068554	-2.880004	6.466496
37	1	0	5.520338	-3.113644	4.671744
38	1	0	2.496176	-4.808280	3.845606
39	6	0	-4.248626	3.082706	2.114056
40	1	0	-4.325941	2.157153	2.712964
41	1	0	-3.628341	3.793525	2.681325
42	1	0	-3.357080	3.657835	0.234764
43	6	0	-5.616243	3.661487	1.884944
44	6	0	-5.962875	4.929114	2.144692
45	1	0	-6.372793	2.983794	1.468826
46	1	0	-6.976366	5.295860	1.954304
47	1	0	-5.248307	5.647096	2.563411
48	8	0	1.285020	-2.850200	2.012628
49	6	0	1.701642	-3.791714	1.011012
50	1	0	2.233929	-4.631184	1.484778
51	1	0	0.111712	-0.453641	4.920430
52	6	0	-1.114663	-1.615307	3.599619
53	1	0	-1.884742	-0.828467	3.596581
54	1	0	-1.054529	-2.034226	2.581809

55	6	0	-1.511307	-2.687664	4.577055
56	6	0	-2.594312	-2.641154	5.362283
57	1	0	-0.855393	-3.564979	4.630885
58	1	0	-2.837180	-3.457591	6.049187
59	1	0	-3.283510	-1.789174	5.345113
60	6	0	2.644626	-3.117504	0.037265
61	6	0	2.349674	-1.887318	-0.570968
62	6	0	3.867480	-3.689725	-0.335848
63	7	0	3.164839	-1.300423	-1.446936
64	1	0	1.420216	-1.355752	-0.341882
65	7	0	4.700230	-3.120327	-1.209775
66	1	0	4.185151	-4.649095	0.090094
67	6	0	4.313653	-1.943410	-1.734980
68	6	0	5.198190	-1.318189	-2.680902
69	6	0	5.946587	-0.786156	-3.484385
70	6	0	6.852215	-0.144324	-4.451918
71	6	0	7.620421	-1.237120	-5.229862
72	1	0	8.304417	-0.765945	-5.954256
73	1	0	8.215086	-1.863482	-4.547091
74	1	0	6.926760	-1.889823	-5.782205
75	6	0	6.022654	0.716883	-5.431395
76	1	0	5.466559	1.500787	-4.894239
77	1	0	6.693552	1.202382	-6.158545
78	1	0	5.299500	0.098909	-5.985782
79	6	0	7.851202	0.751751	-3.682031
80	1	0	7.324265	1.536227	-3.117001
81	1	0	8.450565	0.158237	-2.974513
82	1	0	8.536208	1.237420	-4.395855
83	6	0	-4.238729	1.738167	-0.041307
84	6	0	-5.012976	2.135997	-1.139542
85	6	0	-4.243119	0.359324	0.216735
86	7	0	-5.716628	1.280523	-1.885574
87	1	0	-5.061298	3.192107	-1.430998
88	7	0	-4.938292	-0.516251	-0.511500
89	1	0	-3.649497	-0.052080	1.039889
90	6	0	-5.655551	-0.017117	-1.536994
91	6	0	-6.415986	-0.949736	-2.326621
92	6	0	-7.061890	-1.737740	-2.997823
93	6	0	-7.844456	-2.683467	-3.811876
94	6	0	-7.442138	-4.129555	-3.444481
95	1	0	-6.372308	-4.302945	-3.638630
96	1	0	-7.637736	-4.335462	-2.380688
97	1	0	-8.024819	-4.843211	-4.049381
98	6	0	-7.555112	-2.419566	-5.307814
99	1	0	-8.139671	-3.118839	-5.927569
100	1	0	-7.832173	-1.391568	-5.588080
101	1	0	-6.487514	-2.564134	-5.534902
102	6	0	-9.348814	-2.467047	-3.524278
103	1	0	-9.577797	-2.645933	-2.462267
104	1	0	-9.655372	-1.440187	-3.776694
105	1	0	-9.945872	-3.167263	-4.130926
106	6	0	0.685450	0.112804	2.959272
107	6	0	-0.206222	0.900540	2.234174
108	6	0	2.040345	0.424180	2.779962
109	7	0	0.198300	1.879558	1.398316
110	1	0	-1.288423	0.799306	2.295212
111	7	0	2.452602	1.375227	1.946379
112	1	0	2.818859	-0.115434	3.327355
113	6	0	1.543180	2.080228	1.262975
114	6	0	2.068529	3.063488	0.371621
115	6	0	2.675771	3.831247	-0.357281
116	6	0	3.396578	4.757318	-1.242832
117	6	0	3.697300	6.058704	-0.460890
118	1	0	4.315277	5.853034	0.426539
119	1	0	4.245677	6.757485	-1.112919
120	1	0	2.767252	6.547746	-0.132432

121	6	0	4.720521	4.090345	-1.684571
122	1	0	4.527399	3.153376	-2.229565
123	1	0	5.272467	4.774491	-2.349092
124	1	0	5.355645	3.860008	-0.815498
125	6	0	2.524539	5.074063	-2.479084
126	1	0	2.293443	4.159058	-3.045592
127	1	0	1.577059	5.551137	-2.185387
128	1	0	3.068329	5.764731	-3.143501

8b(*R*)

Sum of electronic and zero-point Energies= -2584.183138
Sum of electronic and thermal Energies= -2584.112198
Sum of electronic and thermal Enthalpies= -2584.111254
Sum of electronic and thermal Free Energies= -2584.305170

Standard orientation:

Center Number	Atomic Number	Atomic Type	Coordinates (Angstroms) X	Y	Z
1	8	0	-2.260688	2.375544	0.543655
2	6	0	-3.384687	2.866645	-0.149095
3	5	0	-0.894209	2.734240	0.103312
4	6	0	-0.606598	4.356129	0.220743
5	1	0	0.339991	4.641074	-0.266217
6	1	0	-1.409835	4.827505	-0.381489
7	6	0	-0.654357	4.904727	1.611524
8	6	0	0.339212	5.538302	2.256758
9	1	0	-1.591292	4.735218	2.160107
10	1	0	0.226110	5.879089	3.291381
11	1	0	1.302235	5.743139	1.774269
12	6	0	-0.537175	2.136308	-1.392796
13	1	0	0.423894	2.522136	-1.768618
14	6	0	-0.571884	0.645690	-1.504783
15	6	0	0.449960	-0.156642	-1.848448
16	1	0	-1.527776	0.169023	-1.250017
17	1	0	0.335194	-1.245422	-1.878593
18	1	0	1.433792	0.242764	-2.121827
19	1	0	-1.318132	2.566321	-2.052499
20	8	0	-0.044249	-1.685536	4.138685
21	6	0	-0.496116	-0.346258	4.245592
22	5	0	-0.054900	-2.588416	3.031727
23	6	0	-1.219006	-2.522978	1.881673
24	1	0	-1.433004	-1.469313	1.622764
25	1	0	-0.834245	-2.993492	0.958697
26	6	0	-2.489712	-3.215819	2.275756
27	6	0	-2.967079	-4.350558	1.741286
28	1	0	-3.061871	-2.760399	3.094763
29	1	0	-3.893085	-4.808818	2.102950
30	1	0	-2.454688	-4.860696	0.916996
31	6	0	0.281558	-4.099087	3.566470
32	1	0	0.167883	-4.854907	2.768320
33	6	0	1.606637	-4.239079	4.238717
34	6	0	2.618995	-5.032355	3.845560
35	1	0	1.766355	-3.588069	5.108501
36	1	0	3.576622	-5.047202	4.376259
37	1	0	2.519748	-5.718650	2.995659
38	1	0	-0.526850	-4.303820	4.294480
39	6	0	-4.350975	3.516240	0.877502
40	1	0	-4.615383	2.738392	1.616678
41	1	0	-3.785163	4.297652	1.407805
42	1	0	-3.093236	3.653195	-0.869005
43	6	0	-5.599065	4.100325	0.278109
44	6	0	-5.880609	5.408827	0.223200
45	1	0	-6.319129	3.388638	-0.145969
46	1	0	-6.806900	5.776667	-0.229132
47	1	0	-5.198532	6.162070	0.634141
48	8	0	1.350806	-2.116444	2.114279
49	6	0	1.948952	-2.906975	1.363258
50	1	0	1.556556	-3.928436	1.222306
51	1	0	-0.077845	0.009779	5.204008
52	6	0	-2.026254	-0.212643	4.364412
53	1	0	-2.273201	0.855118	4.484561
54	1	0	-2.497504	-0.551168	3.426070

55	6	0	-2.584453	-0.992835	5.521763
56	6	0	-3.311982	-0.469665	6.516638
57	1	0	-2.360243	-2.065748	5.530344
58	1	0	-3.694402	-1.091287	7.332228
59	1	0	-3.557942	0.597913	6.553158
60	6	0	3.135955	-2.560717	0.604757
61	6	0	3.760208	-1.293194	0.627850
62	6	0	3.720642	-3.522622	-0.243760
63	7	0	4.824700	-1.023305	-0.115601
64	1	0	3.379726	-0.483486	1.258760
65	7	0	4.787731	-3.265184	-0.988693
66	1	0	3.297111	-4.531817	-0.311556
67	6	0	5.299391	-2.017971	-0.901706
68	6	0	6.440202	-1.719356	-1.708332
69	6	0	7.414121	-1.457237	-2.397405
70	6	0	8.582355	-1.129757	-3.226692
71	6	0	9.133791	-2.420625	-3.872649
72	1	0	10.008317	-2.176415	-4.496645
73	1	0	9.446844	-3.144022	-3.104168
74	1	0	8.374462	-2.898851	-4.510277
75	6	0	8.139875	-0.133609	-4.326081
76	1	0	7.738181	0.791107	-3.883956
77	1	0	9.009046	0.127086	-4.950986
78	1	0	7.366680	-0.575756	-4.972846
79	6	0	9.662862	-0.477271	-2.332171
80	1	0	9.283620	0.442192	-1.860614
81	1	0	9.987952	-1.166980	-1.538178
82	1	0	10.539322	-0.218249	-2.947485
83	6	0	-4.069243	1.762235	-0.938542
84	6	0	-4.531779	1.948796	-2.247955
85	6	0	-4.304026	0.486058	-0.404795
86	7	0	-5.159309	0.997852	-2.944855
87	1	0	-4.383332	2.909065	-2.756485
88	7	0	-4.930371	-0.480479	-1.078122
89	1	0	-3.963713	0.234128	0.605221
90	6	0	-5.338931	-0.183233	-2.326921
91	6	0	-6.021612	-1.216532	-3.060461
92	6	0	-6.602561	-2.091384	-3.681569
93	6	0	-7.299475	-3.152412	-4.428562
94	6	0	-8.287628	-3.871679	-3.481651
95	1	0	-7.758830	-4.322452	-2.627657
96	1	0	-9.040957	-3.170357	-3.090529
97	1	0	-8.810485	-4.672584	-4.029378
98	6	0	-6.253267	-4.161901	-4.957058
99	1	0	-6.762492	-4.962162	-5.518438
100	1	0	-5.533981	-3.669657	-5.629764
101	1	0	-5.693803	-4.621260	-4.127540
102	6	0	-8.067241	-2.523650	-5.613217
103	1	0	-8.814119	-1.796773	-5.258017
104	1	0	-7.379369	-2.004448	-6.298436
105	1	0	-8.591231	-3.311808	-6.177996
106	6	0	0.124666	0.542768	3.176812
107	6	0	-0.575071	1.215546	2.181127
108	6	0	1.514893	0.715921	3.154007
109	7	0	0.041838	1.956858	1.232562
110	1	0	-1.660032	1.207412	2.093104
111	7	0	2.136548	1.424832	2.218097
112	1	0	2.146064	0.251701	3.918903
113	6	0	1.402979	2.022229	1.267106
114	6	0	2.154046	2.726381	0.278114
115	6	0	2.970490	3.235577	-0.473174
116	6	0	3.962727	3.821571	-1.386010
117	6	0	4.540278	2.684944	-2.265519
118	1	0	3.755126	2.233037	-2.891154
119	1	0	5.318122	3.096068	-2.929268
120	1	0	4.988744	1.893381	-1.645515

121	6	0	3.294029	4.894497	-2.273819
122	1	0	2.879518	5.711657	-1.663598
123	1	0	4.042904	5.321355	-2.960079
124	1	0	2.480193	4.462098	-2.875724
125	6	0	5.093294	4.460245	-0.545277
126	1	0	4.701496	5.261643	0.099984
127	1	0	5.583834	3.708858	0.092119
128	1	0	5.849624	4.894866	-1.218493

TS2b(*R*)

Sum of electronic and zero-point Energies= -2584.174186
Sum of electronic and thermal Energies= -2584.104745
Sum of electronic and thermal Enthalpies= -2584.103801
Sum of electronic and thermal Free Energies= -2584.293409

Standard orientation:

Center Number	Atomic Number	Atomic Type	Coordinates (Angstroms) X	Y	Z
1	8	0	-2.160189	2.273333	0.629943
2	6	0	-3.341949	2.801444	0.074840
3	5	0	-0.837246	2.620881	0.063447
4	6	0	-0.522408	4.239830	0.160610
5	1	0	0.384934	4.512296	-0.402030
6	1	0	-1.365604	4.723427	-0.373222
7	6	0	-0.448030	4.787226	1.550549
8	6	0	0.604340	5.405227	2.112317
9	1	0	-1.339519	4.631494	2.173958
10	1	0	0.581328	5.746289	3.152763
11	1	0	1.527627	5.596468	1.552665
12	6	0	-0.632884	2.031645	-1.462342
13	1	0	0.292774	2.406345	-1.926996
14	6	0	-0.706873	0.543765	-1.591108
15	6	0	0.253865	-0.270114	-2.060372
16	1	0	-1.639452	0.079858	-1.243206
17	1	0	0.109676	-1.354848	-2.103750
18	1	0	1.211502	0.114279	-2.430693
19	1	0	-1.467081	2.482010	-2.038457
20	8	0	0.161101	-1.708038	4.094111
21	6	0	-0.108824	-0.323272	4.248508
22	5	0	0.065493	-2.516715	2.912635
23	6	0	-1.319518	-2.512196	2.029447
24	1	0	-1.511854	-1.454131	1.763777
25	1	0	-1.150444	-3.041611	1.074427
26	6	0	-2.531155	-3.086228	2.692525
27	6	0	-3.198093	-4.183687	2.300635
28	1	0	-2.883743	-2.571131	3.595306
29	1	0	-4.064713	-4.556674	2.855689
30	1	0	-2.909786	-4.744733	1.403750
31	6	0	0.350313	-4.206570	3.467099
32	1	0	-0.482541	-4.759257	3.009931
33	6	0	1.646090	-4.605690	3.020005
34	6	0	1.928952	-4.968702	1.715266
35	1	0	2.500093	-4.369096	3.668126
36	1	0	2.954173	-5.211006	1.424346
37	1	0	1.138360	-5.374793	1.077193
38	1	0	0.242181	-4.117075	4.554701
39	6	0	-4.207603	3.377831	1.227278
40	1	0	-4.401874	2.552086	1.935615
41	1	0	-3.594018	4.126190	1.752309
42	1	0	-3.117319	3.634455	-0.616308
43	6	0	-5.506291	3.991935	0.786641
44	6	0	-5.790633	5.299888	0.839471
45	1	0	-6.264064	3.304312	0.389466
46	1	0	-6.755385	5.690019	0.500383
47	1	0	-5.072116	6.030038	1.229442
48	8	0	1.250309	-2.197480	2.021865
49	6	0	1.622203	-2.901718	0.999681
50	1	0	0.849756	-3.308743	0.334810
51	1	0	0.476274	-0.019110	5.134609
52	6	0	-1.586152	-0.028560	4.576156
53	1	0	-1.712149	1.060884	4.690377
54	1	0	-2.216531	-0.342426	3.726403

55	6	0	-2.043706	-0.726666	5.826221
56	6	0	-2.591626	-0.119348	6.886320
57	1	0	-1.899472	-1.813222	5.845939
58	1	0	-2.907293	-0.685080	7.768505
59	1	0	-2.751166	0.964809	6.912807
60	6	0	2.914413	-2.590771	0.377026
61	6	0	3.940007	-1.884371	1.033020
62	6	0	3.193683	-2.975178	-0.946780
63	7	0	5.096660	-1.595906	0.444716
64	1	0	3.810192	-1.550011	2.066535
65	7	0	4.343684	-2.691613	-1.550538
66	1	0	2.449750	-3.526871	-1.533861
67	6	0	5.256199	-2.007570	-0.830248
68	6	0	6.498571	-1.690014	-1.473501
69	6	0	7.553965	-1.417249	-2.022989
70	6	0	8.827013	-1.084791	-2.682303
71	6	0	9.010516	0.451141	-2.670616
72	1	0	9.963379	0.711877	-3.158967
73	1	0	8.193758	0.950629	-3.214401
74	1	0	9.029645	0.838931	-1.640495
75	6	0	9.983731	-1.755731	-1.904556
76	1	0	9.870929	-2.850855	-1.895507
77	1	0	10.943162	-1.509519	-2.387523
78	1	0	10.017459	-1.401345	-0.862853
79	6	0	8.799515	-1.602447	-4.138056
80	1	0	8.664833	-2.694783	-4.166143
81	1	0	7.978748	-1.138405	-4.706525
82	1	0	9.750479	-1.356765	-4.637711
83	6	0	-4.100279	1.749087	-0.718596
84	6	0	-4.672961	2.018554	-1.968670
85	6	0	-4.298904	0.442926	-0.246211
86	7	0	-5.364795	1.114390	-2.666912
87	1	0	-4.563035	3.009373	-2.425979

88	7	0	-4.985605	-0.479204	-0.923302
89	1	0	-3.878428	0.128582	0.714943
90	6	0	-5.496732	-0.103599	-2.111763
91	6	0	-6.244518	-1.087778	-2.849381
92	6	0	-6.880636	-1.919111	-3.476178
93	6	0	-7.647013	-2.919349	-4.238906
94	6	0	-7.894264	-4.160079	-3.351468
95	1	0	-6.942699	-4.613986	-3.034042
96	1	0	-8.466527	-3.892706	-2.449629
97	1	0	-8.467572	-4.913482	-3.915802
98	6	0	-6.835473	-3.322903	-5.492336
99	1	0	-7.399548	-4.070705	-6.073262
100	1	0	-6.646681	-2.451345	-6.137978
101	1	0	-5.865746	-3.761612	-5.210594
102	6	0	-8.997508	-2.300269	-4.667953
103	1	0	-9.592652	-2.004176	-3.790191
104	1	0	-8.841686	-1.409956	-5.296596
105	1	0	-9.576558	-3.038023	-5.246940
106	6	0	0.438816	0.502309	3.092200
107	6	0	-0.340521	1.141470	2.133345
108	6	0	1.823212	0.658424	2.937841
109	7	0	0.194063	1.837365	1.104999
110	1	0	-1.429055	1.146990	2.146639
111	7	0	2.364932	1.323487	1.923371
112	1	0	2.516403	0.228856	3.668757
113	6	0	1.555333	1.886922	1.016441
114	6	0	2.214125	2.556445	-0.059222
115	6	0	2.930553	3.064921	-0.906674
116	6	0	3.784842	3.687757	-1.928772
117	6	0	4.168401	5.109217	-1.450677
118	1	0	4.717637	5.070971	-0.497491
119	1	0	4.813934	5.585821	-2.205867
120	1	0	3.273978	5.736147	-1.312936

121	6	0	5.060227	2.829858	-2.102185
122	1	0	4.810149	1.812177	-2.439433
123	1	0	5.715620	3.294923	-2.856242
124	1	0	5.616960	2.752521	-1.155816
125	6	0	3.016288	3.771655	-3.267066
126	1	0	2.729411	2.770296	-3.622955
127	1	0	2.104413	4.379577	-3.164649
128	1	0	3.659494	4.238476	-4.030287

9b(*R*)

Sum of electronic and zero-point Energies= -2584.234723
Sum of electronic and thermal Energies= -2584.164393
Sum of electronic and thermal Enthalpies= -2584.163448
Sum of electronic and thermal Free Energies= -2584.357617

Standard orientation:

Center Number	Atomic Number	Atomic Type	Coordinates (Angstroms) X	Y	Z
1	8	0	-3.617668	1.791842	0.955405
2	6	0	-5.015746	1.686646	0.817142
3	5	0	-2.854577	2.769412	0.147224
4	6	0	-3.251765	4.325660	0.525155
5	1	0	-2.815831	5.046533	-0.184560
6	1	0	-4.349306	4.375878	0.373019
7	6	0	-2.934966	4.730588	1.930035
8	6	0	-2.130751	5.736676	2.311794
9	1	0	-3.386875	4.112889	2.718520
10	1	0	-1.930304	5.941502	3.368783
11	1	0	-1.644913	6.396011	1.582893
12	6	0	-2.946299	2.457989	-1.469791
13	1	0	-2.498391	3.268073	-2.066906
14	6	0	-2.383879	1.137361	-1.890503
15	6	0	-1.358324	0.936085	-2.735237
16	1	0	-2.845837	0.252629	-1.432137
17	1	0	-0.994725	-0.071592	-2.962796
18	1	0	-0.849602	1.768030	-3.236541
19	1	0	-4.032109	2.477724	-1.693820
20	8	0	1.594173	-0.624661	2.809413
21	6	0	0.426268	0.132167	3.120393
22	5	0	1.851470	-1.412307	1.710772
23	6	0	0.858976	-1.663130	0.484358
24	1	0	0.019573	-0.948780	0.534731
25	1	0	1.385379	-1.445075	-0.462622
26	6	0	0.297981	-3.062852	0.441583
27	6	0	0.438474	-3.937731	-0.564337
28	1	0	-0.279768	-3.375314	1.321567
29	1	0	-0.007813	-4.935739	-0.516530
30	1	0	0.989705	-3.686289	-1.477732
31	6	0	5.509426	-5.914260	0.512726
32	6	0	4.352657	-5.245413	0.428237
33	6	0	3.871595	-4.220288	1.414571
34	1	0	3.681955	-5.446174	-0.417724
35	1	0	2.873079	-4.511051	1.785108
36	1	0	4.548042	-4.159503	2.281867
37	6	0	-5.663680	1.834371	2.220788
38	1	0	-5.236500	1.042578	2.862643
39	1	0	-5.337685	2.803401	2.629032
40	1	0	-5.414669	2.499854	0.183329
41	6	0	-7.163970	1.749820	2.231052
42	6	0	-7.985033	2.773282	2.501027
43	1	0	-7.602233	0.772213	1.992296
44	1	0	-9.072759	2.652328	2.490920
45	1	0	-7.601041	3.769590	2.748712
46	8	0	3.102024	-1.971166	1.790592
47	6	0	3.708187	-2.805215	0.808905
48	1	0	0.696540	0.675432	4.042097
49	6	0	-0.782131	-0.757637	3.460865
50	1	0	-1.612056	-0.107966	3.780414
51	1	0	-1.110713	-1.285976	2.548361
52	6	0	-0.483204	-1.752928	4.549147
53	6	0	-1.134360	-1.819801	5.716505
54	1	0	0.327681	-2.463239	4.349373

55	1	0	-0.878206	-2.570727	6.470032
56	1	0	-1.950694	-1.131341	5.962941
57	6	0	5.032924	-2.195833	0.402386
58	6	0	5.822277	-1.447182	1.287558
59	6	0	5.572147	-2.367946	-0.879781
60	7	0	7.010586	-0.943883	0.948162
61	1	0	5.470452	-1.241280	2.303533
62	7	0	6.757184	-1.873157	-1.241653
63	1	0	5.019580	-2.921353	-1.648044
64	6	0	7.436528	-1.184142	-0.306355
65	6	0	8.722024	-0.656600	-0.679802
66	6	0	9.813566	-0.213354	-0.997298
67	6	0	11.131874	0.322312	-1.377103
68	6	0	11.413071	-0.016543	-2.858357
69	1	0	12.399040	0.379806	-3.150584
70	1	0	11.415028	-1.105545	-3.020620
71	1	0	10.651152	0.428921	-3.516632
72	6	0	11.128119	1.856052	-1.178795
73	1	0	10.926533	2.118049	-0.128644
74	1	0	12.111873	2.267474	-1.457512
75	1	0	10.361513	2.333888	-1.808249
76	6	0	12.212982	-0.318818	-0.475775
77	1	0	12.028180	-0.091603	0.585498
78	1	0	12.230993	-1.412906	-0.597809
79	1	0	13.204557	0.077227	-0.748960
80	6	0	-5.397036	0.369124	0.162053
81	6	0	-6.416108	0.266371	-0.794283
82	6	0	-4.765869	-0.841101	0.485418
83	7	0	-6.776802	-0.890176	-1.356340
84	1	0	-6.960418	1.159985	-1.122870
85	7	0	-5.107592	-2.009968	-0.059320
86	1	0	-3.943404	-0.861032	1.208287
87	6	0	-6.108394	-1.988451	-0.961106
88	6	0	-6.491021	-3.243684	-1.552016
89	6	0	-6.818762	-4.307272	-2.052119
90	6	0	-7.210836	-5.590416	-2.659795
91	6	0	-7.163825	-6.697597	-1.581806
92	1	0	-6.150613	-6.797867	-1.162816
93	1	0	-7.858622	-6.475526	-0.756998
94	1	0	-7.452669	-7.663019	-2.028171
95	6	0	-6.220587	-5.927220	-3.799490
96	1	0	-6.502962	-6.886594	-4.262819
97	1	0	-6.234352	-5.148507	-4.577650
98	1	0	-5.192356	-6.015843	-3.416052
99	6	0	-8.642828	-5.468242	-3.228315
100	1	0	-9.363881	-5.224864	-2.432583
101	1	0	-8.696845	-4.680670	-3.995742
102	1	0	-8.943406	-6.423487	-3.688595
103	6	0	0.167742	1.192477	2.057971
104	6	0	-1.097711	1.526495	1.584203
105	6	0	1.228262	1.931304	1.512021
106	7	0	-1.297355	2.484132	0.654220
107	1	0	-2.015298	1.046662	1.919900
108	7	0	1.043403	2.884889	0.604592
109	1	0	2.262003	1.743713	1.819656
110	6	0	-0.201262	3.150305	0.186007
111	6	0	-0.315893	4.187294	-0.787997
112	6	0	-0.244949	5.090704	-1.605931
113	6	0	-0.172658	6.174079	-2.597539
114	6	0	0.020165	5.547432	-3.999869
115	1	0	-0.825818	4.893986	-4.262552
116	1	0	0.084987	6.350704	-4.751228
117	1	0	0.946353	4.954255	-4.044057
118	6	0	-1.481451	6.995806	-2.562830
119	1	0	-1.641565	7.444936	-1.570647
120	1	0	-1.422683	7.808355	-3.304637

121	1	0	-2.352103	6.368010	-2.806256
122	6	0	1.032539	7.081666	-2.257774
123	1	0	0.917486	7.534322	-1.260855
124	1	0	1.973745	6.511107	-2.273478
125	1	0	1.103291	7.891840	-3.001102
126	1	0	6.210946	-5.749148	1.338040
127	1	0	5.796148	-6.656938	-0.238084
128	1	0	3.067638	-2.879920	-0.084206
